# Methodological tools and sensitivity analysis for assessing quality or risk of bias used in systematic reviews published in the high-impact anesthesiology journals

**DOI:** 10.1186/s12874-020-00966-4

**Published:** 2020-05-18

**Authors:** Marija Franka Marušić, Mahir Fidahić, Cristina Mihaela Cepeha, Loredana Gabriela Farcaș, Alexandra Tseke, Livia Puljak

**Affiliations:** 1 School of Medicine, University of Split, Split, Croatia; 2 Medical Faculty, University of Tuzla, Tuzla, Canton Tuzla, Bosnia and Herzegovina; 3 The Victor Babes, University of Medicine and Pharmacy, Timisoara, Romania; 4 Iuliu Hațieganu University of Medicine and Pharmacy, Faculty of Medicine, Cluj-Napoca, Romania; 5 Medical School, National and Kapodistrian University of Athens, Athens, Greece; 6 Center for Evidence-Based Medicine and Health Care, Catholic University of Croatia, Ilica 242, 10000 Zagreb, Croatia

**Keywords:** Sensitivity analysis, Systematic review, Quality assessment, Bias

## Abstract

**Background:**

A crucial element in the systematic review (SR) methodology is the appraisal of included primary studies, using tools for assessment of methodological quality or risk of bias (RoB). SR authors can conduct sensitivity analyses to explore whether their results are sensitive to exclusion of low quality studies or a high RoB. However, it is unknown which tools do SR authors use for assessing quality/RoB, and how they set threshold for quality/RoB in sensitivity analyses. The aim of this study was to assess quality/RoB assessment tools, the types of sensitivity analyses and quality/RoB thresholds for sensitivity analyses used within SRs published in high-impact pain/anesthesiology journals.

**Methods:**

This was a methodological study. We analyzed SRs published from January 2005 to June 2018 in the 25% highest-ranking journals within the Journal Citation Reports (JCR) “Anesthesiology” category. We retrieved the SRs from PubMed. Two authors independently screened records, full texts, and extracted data on quality/RoB tools and sensitivity analyses. We extracted data about quality/RoB tools, types of sensitivity analyses and the thresholds for quality/RoB used in them.

**Results:**

Out of 678 analyzed SRs, 513 (76%) reported the use of quality/RoB assessments. The most commonly reported tools for assessing quality/RoB in the studies were the Cochrane tool for risk of bias assessment (*N* = 251; 37%) and Jadad scale (*N* = 99; 15%). Meta-analysis was conducted in 451 (66%) of SRs and sensitivity analysis in 219/451 (49%). Most commonly, sensitivity analysis was conducted to explore the influence of study quality/RoB (90/219; 41%) on the results. Quality/RoB thresholds used for sensitivity analysis for those studies were clearly reported in 47 (52%) articles that used them. The quality/RoB thresholds used for sensitivity analyses were highly heterogeneous and inconsistent, even when the same tool was used.

**Conclusions:**

A quarter of SRs reported using quality/RoB assessments, and some of them cited tools that are not meant for assessing quality/RoB. Authors who use quality/RoB to explore the robustness of their results in meta-analyses use highly heterogeneous quality/RoB thresholds in sensitivity analyses. Better methodological consistency for quality/RoB sensitivity analyses is needed.

## Background

Systematic reviews (SRs) combine and appraise the available evidence to answer a specific research question. SRs vary in their methods and scope, but they most often follow a systematic methodology, including the following: pre-defined inclusion criteria, a suitable search strategy, quantitative analytical methods if applicable, and a systematic approach to minimizing biases and random errors, all of which is documented in a methods section [1].

Bias is defined as a systematic error, or deviation from the truth, in results or inferences. Biases can vary in magnitude, from small to substantial, and can lead to an underestimation or overestimation of the true intervention effect [2].

A crucial element in the systematic review process is the judgment of included primary studies, using risk of bias (RoB) or methodological quality assessment tools. This enables a judgment whether the results of primary studies can be trusted and whether they should contribute to meta-analyses. To ensure that SRs take quality/RoB into account, it is not sufficient to simply assess the methodological characteristics of the studies or describe those characteristics in a table or text. Reviewers should also use their critical appraisals to inform subsequent review stages, notably that of synthesis/results and the conclusion-drawing [2, 3].

An additional concern are variations regarding the threshold level of primary study quality or RoB above which primary studies are considered eligible for inclusion in a quantitative synthesis. The Cochrane handbook advocates that several strategies can be used to incorporate RoB assessment into analysis when RoB varies across studies in a meta-analysis [4]. This can be addressed in a sensitivity analysis to see how conclusions might be affected if the studies at high risk of bias would be excluded [2].

Paying particular attention to the methodological/reporting quality of SRs in the high-impact journals published in the field of pain is important because pain is the symptom that most commonly brings patients to see a physician. A pain-free life and access to pain treatment is considered a basic human right [5, 6]. However, inadequate pain management is frequent, even in developed countries. This is caused both by insufficient attention devoted to pain measurement and treatment, as well as the fact that, for some painful conditions such as neuropathic pain, there are inadequate treatment options available [5]. A pain-free state is very important for patients. Therefore, interventions for the treatment of pain are of a major public health importance. We have already shown that methodological and reporting quality of SRs published in the highest-ranking journals in the field of pain needs to be improved [7], and therefore further methodological work in this field can help journal editors, reviewers, and authors to improve future studies.

Detweiler et al. have earlier analyzed a sample of studies from the field of anesthesiology and pain, and reported that, although 84% of those studies assessed quality/RoB, many authors applied questionable methods [8]. They reported that Jadad tool was used most commonly [8], but this tool is nowadays less used, in favor of the Cochrane RoB tool [9]. It is unclear how the usage of different quality/RoB tools is changing over time, and how authors of SRs use sensitivity analysis when they want to check robustness of their result following quality/RoB indicators.

The aim of this study was to assess quality/RoB assessment tools, the types of sensitivity analyses and quality/RoB thresholds for sensitivity analyses used within SRs published in the high-impact pain/anesthesiology journals.

## Methods

### Data sources and study eligibility

We conducted a methodological study, i.e. a research-on-research study. We used an a priori defined research protocol; this protocol is available in Supplementary file 1. We analyzed systematic reviews and meta-analyses published in the 25% highest-ranking journals within the Journal Citation Reports (JCR) category “Anesthesiology”. We limited our analysis to systematic reviews and meta-analyses published between January 2005 and June 2018. We did not include reviews published before 2005, since risk of bias assessment methodology is relatively recent, and the initial version of the Cochrane’s risk of bias tool was published in 2008 [9].

We performed the search on July 3, 2018.

The following 7 journals were analyzed: *Anaesthesia*, *Anesthesia and Analgesia*, *Anesthesiology*, *British Journal of Anaesthesia*, *Pain*, *Pain Physician*, *Regional Anesthesia & Pain Medicine*. We did not use any language restrictions, as all the targeted journals publish articles in English.

Systematic reviews of both randomized and non-randomized studies were eligible. We excluded systematic reviews and meta-analyses of diagnostic accuracy or of individual patient data, as well as overviews of systematic reviews and guidelines. We also excluded systematic reviews published in a short form as a correspondence, and Cochrane reviews published as secondary articles in the analyzed journals. We did not include Cochrane reviews because for them use of Cochrane RoB tool is mandatory.

### Definitions

For the purpose of this study, a systematic review was defined as an overview of scientific studies using explicit and systematic methods to locate, select, appraise, and synthesize relevant and reliable evidence. While meta-analysis is a statistical method used to pool results from more than one study, sometimes the terms “systematic review” and “meta-analysis” are used interchangeably, so we also included studies that were described by authors as a meta-analysis, if they fitted the definition of a systematic review.

While the Cochrane recommends using its RoB tool to assess the quality of individual studies included in their SRs, many systematic reviews use various quality assessment tools for appraising studies. Sometimes authors use the terms “quality” and “bias” interchangeably. Therefore, in this study we analyzed any quality/RoB tool used by the SR authors, regardless of whether the authors called it a quality assessment tool, or a risk of bias assessment tool.

### Search

We searched PubMed by using the advanced search with a journal name, a filter for systematic reviews and meta-analyses, and a filter for publication dates from January 2005 to June 2018. Search results were then exported and saved. The chosen publication dates and the included sample size were considered sufficient based on a previous similar publication [10].

### Screening of records

A calibration exercise was performed on hundred first records to ensure compliance with eligibility criteria. Two authors independently performed each step in screening all the studies. The first step was the screening of titles and abstracts; the second step was the screening of full texts that were retained as eligible or potentially eligible in the first screening step. Disagreements about inclusion of full texts were resolved via discussion or discussion with the third author.

### Data extraction

Two authors independently conducted data extraction, using a standardized data extraction form created for this study. Disagreements were resolved by a discussion with the third author.

Following the initial piloting on 10 reviews, two authors extracted data independently from each eligible study using the standardized extraction form. A third author compared two data sets and identified any possible discrepancies that were resolved by discussion with a third author and resulted in a final consensus.

The following data were extracted: i) the country of authors’ affiliations (the whole count method was used, in which each country gets one mention when it appears in the address of an author, regardless of the number of times it was used for other authors), ii) the number of authors, iii) whether the involvement of a methodologist or statistician was mentioned in the Methods section, iv) whether a meta-analysis was performed, v) whether quality or RoB assessment was performed, vi) the name of the specific quality/RoB tool (extracted verbatim, in the way the authors reported it), and vii) the name of the journal. We also recorded whether a threshold level of quality/RoB was set by the authors.

Apart from analyzing the quality/RoB assessment tools, we also analyzed whether the authors used or planned to use a sensitivity analysis. We analyzed whether the study mentioned sensitivity analysis in the Methods section, regardless of whether it was actually conducted or not, because sensitivity analyses may be planned, but not conducted if they are not feasible subsequently. We also analyzed the frequency of use of sensitivity analyses, and which issues were explored in sensitivity analyses. If sensitivity analysis was done for quality/ RoB, we analyzed how did the authors define quality/RoB threshold (for example, authors may report “sensitivity analysis was conducted by excluding trials at high risk of bias”, but if they do not define what did they consider a study at high risk of bias, a reader cannot know which quality threshold was used for such analysis). We did not have an a priori definition of what a sensitivity analysis is or should be; instead, we extracted all the information that study authors reported as a method of sensitivity analysis.

### Data analysis

A descriptive statistical analysis was performed, including frequencies and percentages, using GraphPad Prism (GraphPad Software, La Jolla, CA, USA).

## Results

We retrieved 1413 results via a database search. After screening, we included 678 studies that were eligible as systematic reviews/meta-analyses. List of included studies is available in Supplementary file 2. The authors’ affiliations originated from 48 countries, most commonly from the USA (*N* = 230; 34%), Canada (*N* = 124; 18%), UK (*N* = 120; 18%), Australia (*N* = 56; 8%) and Germany (*N* = 56; 8%). The median number of authors was 5 (range: 1 to 16). In 35 (5.1%) articles it was stated that a methodologist/statistician was involved in the study. In our sample of 678 SRs, 382 (56%) included only RCTs, 181 (27%) included both RCT and non-randomized studies, 72 (11%) included non-randomized studies, while the remaining 43 (6%) did not report which types of studies were eligible.

### Quality/risk of bias assessment tools

Authors reported that they assessed “quality” or “risk of bias” in 513 (76%) of the included studies. Some articles (*N* = 75; 11%) reported using more than one tool for assessing quality/RoB (range: 2–4). The most commonly reported quality/RoB tools used were the Cochrane tool for RoB assessment (37%) and the Jadad tool (15%), either as a non-modified or a modified version (Table 1). Among studies that reported that only non-randomized studies were eligible, none of the studies reported using “Cochrane risk of bias tool” (with only that expression); two of those reviews reported using modified Cochrane risk of bias tool for observational studies, and one reported use of “ACROBAT-NRSI: Cochrane RoB tool for nonrandomized studies”.

**Table 1:** Tools reported by the systematic review/meta-analysis authors that were used for the assessment of quality or risk of bias of the included studies more than once (*N* = 678)

Tool	***N*** (%)
Cochrane tool for RoB assessment	251 (37)
Non-modified version	241 (36)
Modified version	10 (1.4)
Jadad tool	99 (15)
Non-modified version	92 (14)
Modified version	7 (1.0)
Newcastle-Ottawa scale or its adapted version	30 (4.4)
Oxford scale	29 (4.3)
Non-modified version	10 (1.5)
Modified version	19 (2.7)
Criteria of Agency for Healthcare Research and Quality (AHRQ)	24 (3.5)
Grading of Recommendations Assessment, Development and Evaluation (GRADE)	18 (2.7)
Quality of Reporting of Meta-analyses (QUOROM)	14 (2.0)
Preferred reporting items for systematic review and meta-analysis (PRISMA)	10 (1.5)
Quality Assessment of Diagnostic Accuracy Studies (QUADAS) or QUADAS-2	7 (1.0)
Criteria of the U.S. Preventive Services Task Force (USPSTF)	5 (0.7)
Consolidated Standards of Reporting Trials (CONSORT)	4 (0.6)
The Scottish Intercollegiate Guidelines Network (SIGN) checklist for RCTs	4 (0.6)
Quality in prognosis studies (QUIPS) tool	3 (0.4)
Downs and Black	3 (0.4)
Meta-Analysis of Observational Studies in Epidemiology (MOOSE) checklist	2 (0.3)
Physiotherapy Evidence Database (PEDro) evaluation scale	2 (0.3)
Strengthening of the Reporting of Observational Studies in Epidemiology (STROBE)	2 (0.3)
Centre for Reviews and Dissemination (CRD) recommendations checklist	2 (0.3)

**Fig. 1:**
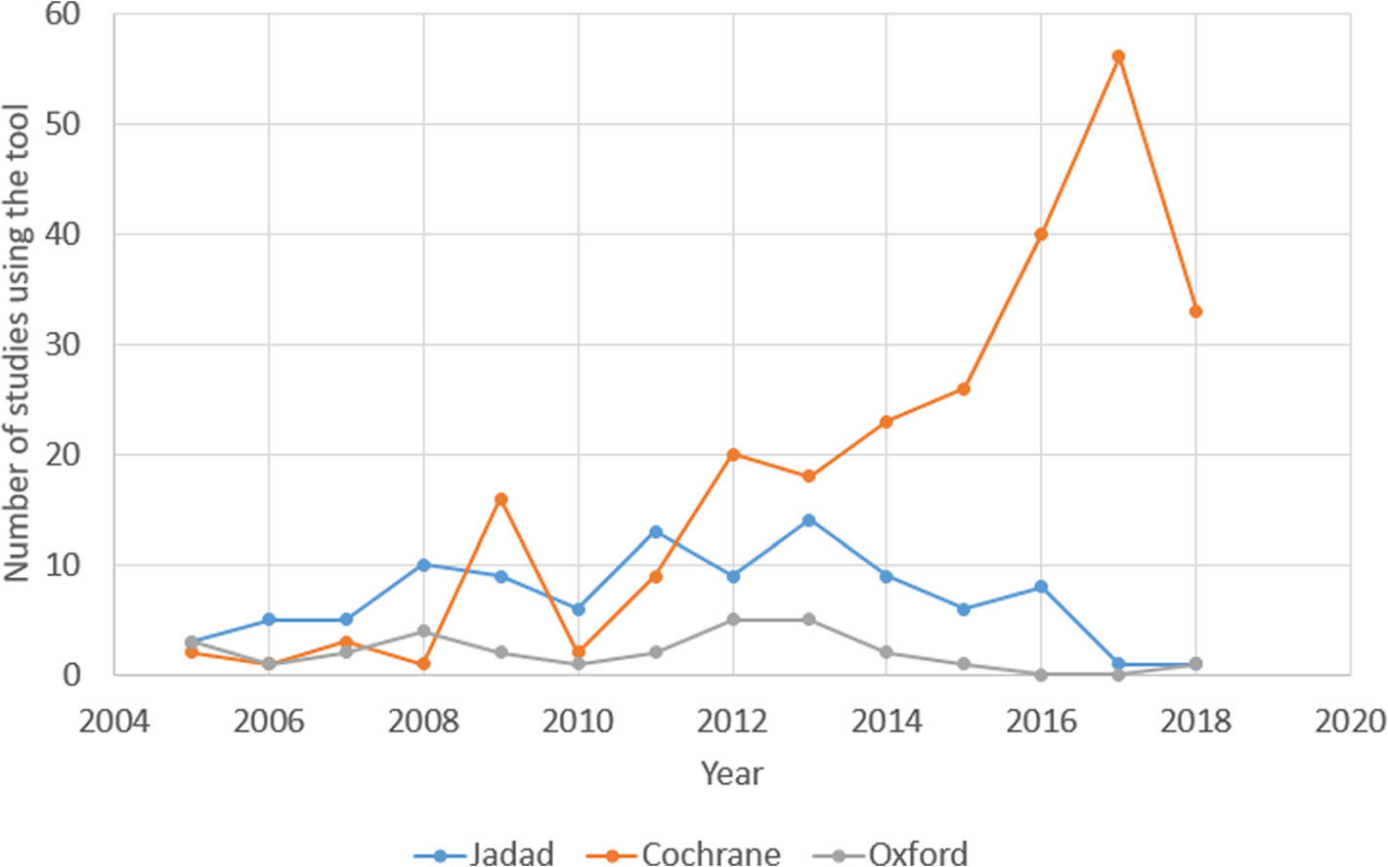
The time trend of using quality/risk of bias tools in the analyzed articles. The three most commonly used quality/risk of bias tools in articles analyzed within this study were Cochrane, Jadad, and Oxford tools. The figure indicates that the usage of Cochrane’s tool is increasing, while the use of Jadad and Oxford tool is decreasing over time. Drop in the use of Cochrane’s tool for RoB assessment in year 2018 is explained by our inclusion criteria – unlike other analyzed years, we included only articles published in the first half of 2018

Some of the tools that authors reported were actually reporting checklists, or were intended for grading of overall evidence, such as QUOROM, PRISMA and GRADE (Table 1). Since we analyzed articles published over the span of 14 years, we noticed a trend of a decrease in the use of the Jadad and Oxford scales, and increased use of the Cochrane tool for RoB assessment (Fig. 1). In 44 (6.5%) articles, the authors reported that they analyzed the quality or RoB of their included studies, but they did not report the name of the tool they used, or provided a reference for the tool they used.

Among 165 reviews that did not report use of quality/RoB tools, 56 (34%) reported that only RCTs were eligible, 47 (28%) included both RCTs and non-randomized studies, 35 (21%) included only non-randomized studies, while 27 (16%) reviews did not report which study designs were eligible.

### Sensitivity analyses

The majority of included articles (*N* = 451; 66%) reported at least one meta-analysis. In 219 (48%) of those 451 articles, the methods for sensitivity analysis were reported. There were 120 of the 219 (55%) studies that performed only one type of sensitivity analysis, while others performed from 2 to 9 various types of sensitivity analyses. Sensitivity analysis was most commonly conducted to explore various aspects of study quality/RoB (90/219; 41%), intervention variations, and various statistical aspects (Table 2).

**Table 2:** Variables analyzed in the sensitivity analyses, used more than 5 times in the analyzed sample (*N* = 678)

Variables	***N*** (%)
Various aspects of quality/risk of bias	90 (13)
Statistics and effect sizes (heterogeneity, effect sizes, imprecise effect estimates, intention-to-treat analysis, different methods for effect size calculations, different results, correlation coefficients, meta-regression, imputation of data, different analysis methods, event rate, standard deviation calculated from standard error)	58 (8.6)
Intervention variations	52 (7.7)
Impact of each individual study (sequential exclusion of single studies)	31 (4.6)
Patients’ characteristics (such as smoking, gender, weight)	24 (3.5)
Type of outcomes (such as different pain scales)	23 (3.4)
Type of included studies (crossover studies, randomized controlled trials, non-randomized studies, non-blinded studies, data from retracted studies, mixed data, peer-reviewed manuscripts)	13 (1.9)
Trial size	7 (1.0)
Publication bias	6 (0.9)
Comparator	6 (0.9)
Covariates	5 (0.7)
Type of funding	5 (0.7)

### Sensitivity analyses for study quality/risk of bias

Among 90 studies that conducted sensitivity analysis based on study quality/RoB, 47 (52%) clearly specified the threshold for defining different levels of study quality/RoB (Supplementary file 3). Those 47 studies provided clear descriptions of what they considered high or low quality studies, or the difference between a low, unclear, or high risk of bias.

However, thresholds for quality/RoB used in those articles were highly heterogeneous. The most common approach in those 47 studies was to use a certain number of points on the Jadad, Oxford or Newcastle-Ottawa scales to define what was considered high or low-quality study (*N* = 19; 40%). The authors did not use consistent cut-off points for labelling high-quality studies (Table 3).

**Table 3:** Specific quality threshold for sensitivity analysis used for different tools

Study	Tool and threshold
Wong, 2013 [11]	Chorti et al. criteria: The maximum score of the checklist is 26; 50% of maximum score is cut-off for high-quality study
Grant, 2016 [12]	Jadad: high risk of bias Jadad score < 4
Johnson, 2007 [13]	Jadad: limiting the analysis to those studies with a Jadad score of at least 4
Raiman, 2016 [14]	Jadad: removing high bias studies (Jadad score < 3)
Hamilton, 2011 [15]	Jadad: score 3 classified as a higher quality study
Hauser, 2011 [16]	Jadad: studies with a low (1 to 2) and moderate (3 to 5) Jadad score
Toner, 2017 [17]	Jadad: high-quality trials only (Jadad scale score, 4 to 5).
Aya 2013 [18]	Jadad: score >3 classified as a higher quality study
Morrison, 2013 [19]	Jadad: studies with low quality (Jadad score ≤ 3) vs studies with high quality (Jadad score >3)
Wang, 2009 [20]	Jadad: study quality (Jadad score ≥ 3 vs Jadad score ≤ 3)
Sanfilippo, 2017 [21]	Newcastle-Ottawa Scale tool: Low risk of bias score ranging between 6 and 9
Nagappa, 2017 [22]	Newcastle-Ottawa scale: good quality is score ≥ 8 of 9
Schnabel, 2011 [23]	Oxford scale: low quality study with 2 points
Schnabel, 2010 [24]	Oxford scale: the studies were rated as high (Oxford scale ≥3) or low (Oxford scale >3) quality studies.
Suppan, 2016 [25]	Oxford: lower quality studies (Oxford score < 4)
Schnabel, 2012 [26]	Oxford: ‘high quality’: Oxford scale > 3 versus ‘low quality’: Oxford scale 3 points
Schnabel, 2013 [27]	Oxford: high-quality trials [modified Oxford scale > 4] vs low-quality trials [modified Oxford scale ≤4
Schnabel, 2013 [28]	Oxford: high-quality trials [modified Oxford scale > 4] vs low-quality trials [modified Oxford scale ≤4
Mishriky, 2012 [29]	Oxford: restricting the analysis to studies with a modified Oxford score of 4 or higher

The next most common category used various numbers of individual pre-specified RoB domains (i.e. key domains) for assessing what was a high, unclear, or low RoB. There were 18 such studies and the most commonly used domain for contributing to the assessment of RoB was allocation concealment (used in 7 of 18 articles), followed by the ‘blinding of outcome assessors’ (*N* = 4), the ‘blinding of participants and personnel (*N* = 3), the generation of a randomization sequence (*N* = 3), and attrition bias (*N* = 3). Even the definitions of acceptable attrition varied among those few studies, whereas one article indicated that they used the threshold of 10%, and another one used 20% (Supplementary file 3).

In 10 of 47 (21%) articles, any RoB domain could contribute equally to overall RoB assessment. For example, if any one domain was judged as having a high RoB, the whole study was considered to have a high RoB. Two of those 10 articles used numerical formulas for determining how many domains with high RoB need to be present to qualify the whole study as having a high RoB (e.g. “*A decision to classify “overall bias” as low, unclear, or high was made by the reviewers using the following method: High: any trial with a high risk of bias listed on 3 or more domains*.”) [30].

## Discussion

In a large sample of the systematic reviews and meta-analyses published from 2005 to 2018 in the highest-ranking pain/anesthesiology journals, the authors reported that they assessed quality/RoB in 76% of the articles. The most commonly used tools were the Cochrane RoB tool and Jadad tool, and some of the tools that the authors reported for assessing quality/RoB were not actually tools that are meant to be used for that purpose. A sensitivity analysis based on quality/RoB was performed in less than half of articles that reported using sensitivity analyses, and the thresholds for quality/RoB were highly inconsistent.

In 2016, Detweiler et al. published their report about the usage of RoB and methodological appraisal practices in SRs published in anesthesiology journals, in which they analyzed 207 SRs published from 2007 to 2015. In their analysis, the Jadad tool was the most commonly used for methodological assessment [8]. On the contrary, in our analysis, which included SRs published from 2005 to 2018, with 678 analyzed articles, the Cochrane tool for RoB assessment was overall the most commonly used; our analysis shows that the usage of Cochrane tool for RoB assessment is increasing over time, and that popularity of the Jadad and Oxford scales is decreasing among SR authors. The Cochrane RoB tool 2.0 was announced recently, but none of the reviews included in our analysis have used it.

In most of the studies, a single quality/RoB assessment tool was used, but some studies used multiple tools. In recent years, the Cochrane Risk of Bias tool has become established in the assessment of RoB in randomized controlled trials [2]. However, a significant variation can be observed for RoB assessment in non-randomized trials. It is especially important to assess RoB in observational studies because, unlike controlled experiments or well-planned, experimental randomized clinical trials, observational studies are subject to a number of potential problems that may bias their results [31]. In a 2007 study, 86 tools comprising 53 checklists and 33 scales were found in the literature, following an electronic search performed in March 2005. The majority of those tools included RoB items related to study variables (86%), design-related bias (86%), and confounding (78%), although, for example, assessment of the conflict of interest was under-represented (4%). The number of items ranged from 3 to 36 [32]. An analysis of SRs in the field of epidemiology of chronic disease indicated that only 55% of reviews addressed quality assessment of primary studies [33]. An analysis of interventional SRs within the field of general health research and physical therapy showed that, in addition to the Cochrane RoB tool, 26 quality tools were identified, with an extensive item variation across tools [34].

Although it appears that the majority of the SRs in the highest-ranking pain journals do incorporate some kind of tool for appraising quality of evidence/risk of bias, about half of them then did not determine the level of quality of primary studies as a threshold for conducting numerical analyses and reaching conclusions. This may have directly influenced the conclusions that were derived from the evidence synthesis conducted within the SRs [35]. To prevent biased conclusions based on studies with a flawed methodology, an acceptable threshold of study quality should be clearly specified, preferentially already in the initial SR protocol [36, 37].

In our study, less than half of the analyzed articles reported conducting a sensitivity analysis, and, most commonly, the sensitivity analysis was conducted to test the effect of quality/RoB on the results. Only half of the studies that used sensitivity analyses for quality/RoB have clearly specified a threshold for methodological quality, i.e. what was considered a high or low quality/RoB. Without a clear threshold for methodological quality, it is likely that different studies have different definitions of high and low quality, which may lead to different SR results and conclusions, which is not desirable and does not foster a reproducibility of the results and a consistency of assessment across different systematic reviews. This hypothesis is further confirmed by our findings that the studies where authors reported threshold for quality/RoB had a highly inconsistent approach, even when using the same tool.

Another issue is the diversity of the quality/RoB tools used for methodological quality assessment. These tools can be widely different and the levels of quality may not be comparable if different tools are used. For example, the Jadad scale has faced considerable criticism [4, 38]. Furthermore, the Cochrane Handbook states for the Jadad scale that “the use of this scale is explicitly discouraged” because it suffers from the generic problems of scales, has a strong emphasis on reporting rather than conduct, and does not cover the allocation concealment aspect [39]. Therefore, future SRs should avoid using the Jadad scale for assessing the methodological quality of included studies. As we can see from our results, the usage of both the Jadad and Oxford tools for methodological assessment is decreasing.

Some of the quality assessment tools reported in the SRs we analyzed are actually reporting guidelines/checklists for systematic reviews, such as QUOROM, PRISMA, or MOOSE. This indicates that not all authors of SRs are aware of the proper tools for the methodological assessment of SRs. Our study is, therefore, highlighting the possible lack of knowledge on research methodology among some review authors.

Although we have noted that half of the articles reported clear thresholds for sensitivity analysis related to a methodological assessment, even in those cases the authors rarely provided any more specific information about these thresholds, probably due to the insufficient space and constraining word limits in journals. Namely, even if authors clearly describe that a study will be considered to have a high RoB based on the assessment of RoB in the ‘random sequence generation’ domain, it is still possible that the authors will erroneously assess RoB judgments. Our recent analyses of RoB assessments made by authors of Cochrane reviews showed that many Cochrane reviews have inadequate and inconsistent RoB judgments [40–44].

Our analysis of high-impact anesthesiology journals indicates a considerable inconsistency in the methods used for sensitivity analyses based on quality/RoB. Authors make an assessment of the overall risk of bias on the level of the whole study using different approaches, which may yield widely different conclusions. For example, it is not the same if the authors consider all RoB domains as equally contributing to the overall RoB of a study, or if they define certain key domains.

One solution for improving SRs in terms of their methodological assessments is to provide more detailed journal instructions for authors, where editors can indicate that all SRs need to conduct a methodological quality assessment of included studies and recommend adequate tools. Furthermore, editors and peer-reviewers analyzing submitted SRs should pay attention to adequate quality assessment and whether SRs with an included numerical analysis have conducted sensitivity analyses to account for the effect of study quality/RoB. Editors and peer-reviewers can request clear reporting of the methods that the authors have used. Editors are commonly perceived as gatekeepers protecting from the acceptance of low-quality manuscripts. Most authors will try to comply with editorial suggestions [45].

A limitation of our study is its reliance on reported data. The study authors were not contacted for clarifications regarding analyzed variables. Additionally, our study may be limited by publication bias, i.e. the fact that some results tend to be published in higher ranking journals, independent of the quality of research, just because of the direction of results. By analyzing the highest-ranking 25% of the journals in the chosen field, we may have introduced reporting bias ourselves. Furthermore, we limited our search to studies published from 2005 onwards, because methods for assessing RoB were developed relatively recently. We have searched for studies published in the targeted journals only via PubMed; it is possible that some relevant articles were missed due to erroneous indexing, and that we could have found additional relevant studies by employing additional search sources, such as hand-searching on journal sites, or using another database. We did not include a librarian in designing our search strategy because the search for the targeted articles was simple, using the built-in filters.

Future studies should explore possible interventions for improving systematic review methodology in terms of its analyzing quality, including a sensitivity analysis for study quality, and clearly specifying a threshold. This methodological consistency will ensure a better comparability of the study results.

## Conclusions

Our study indicates that a quarter of the SRs published in the highest-ranking pain journals do not incorporate a methodological assessment of their included primary studies. Among those with meta-analyses, a minority of the SRs had a sensitivity analysis for study quality/RoB performed, and, in only half of those, the methodological quality threshold criteria were clearly defined. Without a consistent quality assessment and clear definitions of quality, untrustworthy evidence is piling up, in whose conclusions one cannot trust, much less safely implement it into clinical practice. Systematic reviews need to appraise their included studies and plan sensitivity analyses because an inclusion of trials with a high RoB has the potential to meaningfully alter the conclusions. The editors and peer-reviewers should act as gatekeepers protecting against the acceptance of systematic reviews that do not account for the quality of their included studies, and do not report their methods adequately, as well as help the authors to become aware of this crucial aspect of systematic review methodology.

## Supplementary information

**Additional file 1.** Study protocol. This file includes the study protocol, which was defined a priori before commencement of the study.

**Additional file 2.** List of included studies. The file includes a list of systematic reviews/meta-analyses analyzed within this study, with their full bibliographic records.

**Additional file 3.** Quality threshold for sensitivity analysis as described in the included studies. Threshold description includes all relevant information for sensitivity analysis from Methods, Results or Discussion, regardless of the part of manuscript where they were described.

## Data Availability

The datasets used and analyzed during the current study are available from the Open Science Framework, at the following link: https://osf.io/739rk/

## Abbreviations

AHRQ: Agency for Healthcare Research and Quality
CONSORT: Consolidated Standards of Reporting Trials
CRD: Centre for Reviews and Dissemination
GRADE: Grading of Recommendations Assessment, Development and Evaluation
JCR: Journal Citation Reports
MOOSE: Meta-Analysis of Observational Studies in Epidemiology
NOS: Newcastle-Ottawa Scale
QUADAS: Quality Assessment of Diagnostic Accuracy Studies
QUADAS-2: Quality Assessment of Diagnostic Accuracy Studies 2
QUIPS: Quality in prognosis studies
QUOROM: Quality of Reporting of Meta-analyses
PEDro: Physiotherapy Evidence Database
PRISMA: Preferred reporting items for systematic review and meta-analysis
RCT: Randomized controlled trial
RoB: Risk of bias
SIGN: Scottish Intercollegiate Guidelines Network
SR: Systematic review
STROBE: Strengthening of the Reporting of Observational Studies in Epidemiology
USPSTF: U.S. Preventive Services Task Force
