# Supplementary material for: Methodological tools and sensitivity analysis for assessing quality or risk of bias used in systematic reviews published in the high-impact anesthesiology journals
Source: BMC Med Res Methodol. 2020 May 18;20:121. doi: 10.1186/s12874-020-00966-4 (PMC7236513; doi:10.1186/s12874-020-00966-4)
Supplement: Supplementary file 1 — Additional file 1. Study protocol. This file includes the study protocol, which was defined a priori before commencement of the study. [file 12874_2020_966_MOESM1_ESM.docx]

**A study protocol:**

**Methodological tools and sensitivity analysis for assessing quality or risk of bias used in systematic reviews published in the high-impact anesthesiology journals**

**Principal investigator:**

Prof. Livia Puljak, MD, PhD

**Study aim**

The aim of this study was to assess quality/RoB assessment tools, the types of sensitivity analyses and quality/RoB thresholds for sensitivity analyses used within SRs published in the high-impact pain/anesthesiology journals.

**Methods**

*Study design*

We will conduct a methodological study, i.e. a research-on-research study.

Study protocol

This study protocol was designed *a priori*, before the study commenced.

*Eligible studies*

Systematic reviews and meta-analyses published between January 2005 and June 2018 in the 25% highest-ranking journals within the Journal Citation Reports (JCR) category “Anesthesiology” will be analyzed.

The following 7 journals will be analyzed: *Anaesthesia*, *Anesthesia and Analgesia*, *Anesthesiology*, *British Journal of Anaesthesia*, *Pain*, *Pain Physician*, *Regional Anesthesia & Pain Medicine*.

We will exclude systematic reviews and meta-analyses of diagnostic accuracy or of individual patient data, as well as overviews of systematic reviews and guidelines. We will also exclude systematic reviews published in a short form as a correspondence, and Cochrane reviews published as secondary articles in the analyzed journals.

*Definitions*

For the purpose of this study, a systematic review is defined as an overview of scientific studies using explicit and systematic methods to locate, select, appraise, and synthesize relevant and reliable evidence. While meta-analysis is a statistical method used to pool results from more than one study, sometimes the terms “systematic review” and “meta-analysis” are used interchangeably, so we will also include studies described by authors as a meta-analysis, if they fitted the definition of a systematic review.

While the Cochrane recommends using risk of bias assessment for the appraisal of included studies, many systematic reviews use various quality assessment tools for appraising studies. Sometimes authors use the terms “quality” and “bias” interchangeably. Therefore, in this study we will analyze any quality/RoB tool used by the SR authors, regardless of whether the authors called it a quality assessment tool, or a risk of bias assessment tool.

*Search*

The MEDLINE database will be searched using the advanced search with a journal name, a filter for systematic reviews and meta-analyses, and a filter for publication dates from January 2005 to June 2018. Search results will then be exported and saved. The chosen publication dates and the included sample size are considered sufficient based on a previous similar publication [[10](#_ENREF_10)].

*Data extraction*

Two authors will independently perform each step in screening all the studies and data extraction, using a standardized data extraction form created for this study. Disagreements will be resolved by a discussion with the third author.

Following the initial piloting on 10 reviews, two authors will extract data independently from each eligible study using the standardized extraction form. A third author will compare two data sets and identify any possible discrepancies that were resolved by discussion with a third author and resulted in a final consensus.

The following data will be extracted: i) the country where the study was conducted (the whole count method was used, in which each country gets one mention when it appears in the address of an author, regardless of the number of times it was used for other authors), ii) the number of authors, iii) whether the involvement of a methodologist or statistician was mentioned in the Methods section, iv) whether a meta-analysis was performed, v) whether quality or RoB assessment was performed, vi) the name of the specific quality/RoB tool, and vii) the name of the journal. We will also record whether a threshold level of quality/RoB was set by the authors.

Apart from analyzing the quality/RoB assessment tools, we will also analyze whether the authors used or planned to use a sensitivity analysis. We will analyze whether the study mentioned sensitivity analysis in the Methods section, regardless of whether it was actually conducted or not, because sensitivity analyses may be planned, but not conducted if they are not feasible subsequently. We will analyze the frequency of use of sensitivity analyses, and which issues were explored in sensitivity analyses. If sensitivity analysis was done for quality/ RoB, we will anlayze how did the authors define quality/RoB threshold (for example, authors may report “sensitivity analysis was conducted by excluding trials at high risk of bias”, but if they do not define what did they consider a study at high risk of bias, a reader cannot know which quality threshold was used for such analysis). We will not have an *a priori* definition of what a sensitivity analysis is or should be; instead, we will extract all the information that study authors reported as a method of sensitivity analysis.

*Data analysis*

A descriptive statistical analysis will be performed, including frequencies and percentages, using GraphPad Prism (GraphPad Software, La Jolla, CA, USA).
