# Supplementary material for: Methodological tools and sensitivity analysis for assessing quality or risk of bias used in systematic reviews published in the high-impact anesthesiology journals
Source: BMC Med Res Methodol. 2020 May 18;20:121. doi: 10.1186/s12874-020-00966-4 (PMC7236513; doi:10.1186/s12874-020-00966-4)
Supplement: Supplementary file 2 — Additional file 2. List of included studies. The file includes a list of systematic reviews/meta-analyses analyzed within this study, with their full bibliographic records. [file 12874_2020_966_MOESM2_ESM.docx]

**Supplementary file 2: List of included systematic reviews [**[**1-182**](#_ENREF_1)**][**[**183-317**](#_ENREF_183)**][**[**318-556**](#_ENREF_318)**][**[**557-676**](#_ENREF_557)**]**[[677](#_ENREF_677), [678](#_ENREF_678)]

1. Abad-Gurumeta A, Ripolles-Melchor J, Casans-Frances R, Espinosa A, Martinez-Hurtado E, Fernandez-Perez C, Ramirez JM, Lopez-Timoneda F, Calvo-Vecino JM, Evidence Anaesthesia Review G: **A systematic review of sugammadex vs neostigmine for reversal of neuromuscular blockade**. *Anaesthesia* 2015, **70**(12):1441-1452.

2. Abbas SM, Hill AG: **Systematic review of the literature for the use of oesophageal Doppler monitor for fluid replacement in major abdominal surgery**. *Anaesthesia* 2008, **63**(1):44-51.

3. Abbott TEF, Ahmad T, Phull MK, Fowler AJ, Hewson R, Biccard BM, Chew MS, Gillies M, Pearse RM, International Surgical Outcomes Study g: **The surgical safety checklist and patient outcomes after surgery: a prospective observational cohort study, systematic review and meta-analysis**. *Br J Anaesth* 2018, **120**(1):146-155.

4. Abbott TEF, Fowler AJ, Pelosi P, Gama de Abreu M, Moller AM, Canet J, Creagh-Brown B, Mythen M, Gin T, Lalu MM *et al*: **A systematic review and consensus definitions for standardised end-points in perioperative medicine: pulmonary complications**. *Br J Anaesth* 2018, **120**(5):1066-1079.

5. Abdallah FW, Abrishami A, Brull R: **The facilitatory effects of intravenous dexmedetomidine on the duration of spinal anesthesia: a systematic review and meta-analysis**. *Anesth Analg* 2013, **117**(1):271-278.

6. Abdallah FW, Brull R: **Is sciatic nerve block advantageous when combined with femoral nerve block for postoperative analgesia following total knee arthroplasty? A systematic review**. *Reg Anesth Pain Med* 2011, **36**(5):493-498.

7. Abdallah FW, Brull R: **Facilitatory effects of perineural dexmedetomidine on neuraxial and peripheral nerve block: a systematic review and meta-analysis**. *Br J Anaesth* 2013, **110**(6):915-925.

8. Abdallah FW, Chan VW, Brull R: **Transversus abdominis plane block: a systematic review**. *Reg Anesth Pain Med* 2012, **37**(2):193-209.

9. Abdallah FW, Halpern SH, Aoyama K, Brull R: **Will the Real Benefits of Single-Shot Interscalene Block Please Stand Up? A Systematic Review and Meta-Analysis**. *Anesth Analg* 2015, **120**(5):1114-1129.

10. Abdallah FW, Halpern SH, Margarido CB: **Transversus abdominis plane block for postoperative analgesia after Caesarean delivery performed under spinal anaesthesia? A systematic review and meta-analysis**. *Br J Anaesth* 2012, **109**(5):679-687.

11. Abdallah FW, Laffey JG, Halpern SH, Brull R: **Duration of analgesic effectiveness after the posterior and lateral transversus abdominis plane block techniques for transverse lower abdominal incisions: a meta-analysis**. *Br J Anaesth* 2013, **111**(5):721-735.

12. Abdi S, Datta S, Trescot AM, Schultz DM, Adlaka R, Atluri SL, Smith HS, Manchikanti L: **Epidural steroids in the management of chronic spinal pain: a systematic review**. *Pain Physician* 2007, **10**(1):185-212.

13. Abdulatif M, Mukhtar A, Obayah G: **Pitfalls in reporting sample size calculation in randomized controlled trials published in leading anaesthesia journals: a systematic review**. *Br J Anaesth* 2015, **115**(5):699-707.

14. Abrahams MS, Aziz MF, Fu RF, Horn JL: **Ultrasound guidance compared with electrical neurostimulation for peripheral nerve block: a systematic review and meta-analysis of randomized controlled trials**. *Br J Anaesth* 2009, **102**(3):408-417.

15. Abrishami A, Chan J, Chung F, Wong J: **Preoperative pain sensitivity and its correlation with postoperative pain and analgesic consumption: a qualitative systematic review**. *Anesthesiology* 2011, **114**(2):445-457.

16. Adams R, Brown GT, Davidson M, Fisher E, Mathisen J, Thomson G, Webster NR: **Efficacy of dexmedetomidine compared with midazolam for sedation in adult intensive care patients: a systematic review**. *Br J Anaesth* 2013, **111**(5):703-710.

17. Adesope O, Ituk U, Habib AS: **Local anaesthetic wound infiltration for postcaesarean section analgesia: A systematic review and meta-analysis**. *Eur J Anaesthesiol* 2016, **33**(10):731-742.

18. Afshari A, Brok J, Moller AM, Wetterslev J: **Inhaled nitric oxide for acute respiratory distress syndrome and acute lung injury in adults and children: a systematic review with meta-analysis and trial sequential analysis**. *Anesth Analg* 2011, **112**(6):1411-1421.

19. Agoliati A, Dexter F, Lok J, Masursky D, Sarwar MF, Stuart SB, Bayman EO, Epstein RH: **Meta-analysis of average and variability of time to extubation comparing isoflurane with desflurane or isoflurane with sevoflurane**. *Anesth Analg* 2010, **110**(5):1433-1439.

20. Ahn EJ, Kang H, Choi GJ, Baek CW, Jung YH, Woo YC: **The Effectiveness of Midazolam for Preventing Postoperative Nausea and Vomiting: A Systematic Review and Meta-Analysis**. *Anesth Analg* 2016, **122**(3):664-676.

21. Aiyer R, Gulati A, Gungor S, Bhatia A, Mehta N: **Treatment of Chronic Pain With Various Buprenorphine Formulations: A Systematic Review of Clinical Studies**. *Anesth Analg* 2018, **127**(2):529-538.

22. Albrecht E, Guyen O, Jacot-Guillarmod A, Kirkham KR: **The analgesic efficacy of local infiltration analgesia vs femoral nerve block after total knee arthroplasty: a systematic review and meta-analysis**. *Br J Anaesth* 2016, **116**(5):597-609.

23. Albrecht E, Kern C, Kirkham KR: **A systematic review and meta-analysis of perineural dexamethasone for peripheral nerve blocks**. *Anaesthesia* 2015, **70**(1):71-83.

24. Albrecht E, Kirkham KR, Liu SS, Brull R: **The analgesic efficacy and safety of neuraxial magnesium sulphate: a quantitative review**. *Anaesthesia* 2013, **68**(2):190-202.

25. Albrecht E, Kirkham KR, Liu SS, Brull R: **Peri-operative intravenous administration of magnesium sulphate and postoperative pain: a meta-analysis**. *Anaesthesia* 2013, **68**(1):79-90.

26. Albrecht E, Mermoud J, Fournier N, Kern C, Kirkham KR: **A systematic review of ultrasound-guided methods for brachial plexus blockade**. *Anaesthesia* 2016, **71**(2):213-227.

27. Aldenkortt M, Lysakowski C, Elia N, Brochard L, Tramer MR: **Ventilation strategies in obese patients undergoing surgery: a quantitative systematic review and meta-analysis**. *Br J Anaesth* 2012, **109**(4):493-502.

28. Allen TK, Habib AS: **P6 stimulation for the prevention of nausea and vomiting associated with cesarean delivery under neuraxial anesthesia: a systematic review of randomized controlled trials**. *Anesth Analg* 2008, **107**(4):1308-1312.

29. Allen TK, Jones CA, Habib AS: **Dexamethasone for the prophylaxis of postoperative nausea and vomiting associated with neuraxial morphine administration: a systematic review and meta-analysis**. *Anesth Analg* 2012, **114**(4):813-822.

30. Allen TK, Mishriky BM, Klinger RY, Habib AS: **The impact of neuraxial clonidine on postoperative analgesia and perioperative adverse effects in women having elective Caesarean section-a systematic review and meta-analysis**. *Br J Anaesth* 2018, **120**(2):228-240.

31. Alphonsus CS, Rodseth RN: **The endothelial glycocalyx: a review of the vascular barrier**. *Anaesthesia* 2014, **69**(7):777-784.

32. Amanzio M, Corazzini LL, Vase L, Benedetti F: **A systematic review of adverse events in placebo groups of anti-migraine clinical trials**. *Pain* 2009, **146**(3):261-269.

33. Andersen LO, Kehlet H: **Analgesic efficacy of local infiltration analgesia in hip and knee arthroplasty: a systematic review**. *Br J Anaesth* 2014, **113**(3):360-374.

34. Andersen LP, Werner MU, Rosenberg J, Gogenur I: **A systematic review of peri-operative melatonin**. *Anaesthesia* 2014, **69**(10):1163-1171.

35. Andersson GB, Mekhail NA, Block JE: **Treatment of intractable discogenic low back pain. A systematic review of spinal fusion and intradiscal electrothermal therapy (IDET)**. *Pain Physician* 2006, **9**(3):237-248.

36. Andreae MH, Andreae DA: **Regional anaesthesia to prevent chronic pain after surgery: a Cochrane systematic review and meta-analysis**. *Br J Anaesth* 2013, **111**(5):711-720.

37. Angst MS, Clark JD: **Opioid-induced hyperalgesia: a qualitative systematic review**. *Anesthesiology* 2006, **104**(3):570-587.

38. Aoki Y, Aoshima Y, Atsumi K, Kaminaka R, Nakau R, Yanagida K, Kora M, Fujii S, Yokoyama J: **Perioperative Amino Acid Infusion for Preventing Hypothermia and Improving Clinical Outcomes During Surgery Under General Anesthesia: A Systematic Review and Meta-analysis**. *Anesth Analg* 2017, **125**(3):793-802.

39. Apfel CC, Heidrich FM, Jukar-Rao S, Jalota L, Hornuss C, Whelan RP, Zhang K, Cakmakkaya OS: **Evidence-based analysis of risk factors for postoperative nausea and vomiting**. *Br J Anaesth* 2012, **109**(5):742-753.

40. Apfel CC, Saxena A, Cakmakkaya OS, Gaiser R, George E, Radke O: **Prevention of postdural puncture headache after accidental dural puncture: a quantitative systematic review**. *Br J Anaesth* 2010, **105**(3):255-263.

41. Apfel CC, Turan A, Souza K, Pergolizzi J, Hornuss C: **Intravenous acetaminophen reduces postoperative nausea and vomiting: a systematic review and meta-analysis**. *Pain* 2013, **154**(5):677-689.

42. Aprili D, Bandschapp O, Rochlitz C, Urwyler A, Ruppen W: **Serious complications associated with external intrathecal catheters used in cancer pain patients: a systematic review and meta-analysis**. *Anesthesiology* 2009, **111**(6):1346-1355.

43. Arnold DM, Fergusson DA, Chan AK, Cook RJ, Fraser GA, Lim W, Blajchman MA, Cook DJ: **Avoiding transfusions in children undergoing cardiac surgery: a meta-analysis of randomized trials of aprotinin**. *Anesth Analg* 2006, **102**(3):731-737.

44. Arulkumaran N, Annear NM, Singer M: **Patients with end-stage renal disease admitted to the intensive care unit: systematic review**. *Br J Anaesth* 2013, **110**(1):13-20.

45. Arulkumaran N, Corredor C, Hamilton MA, Ball J, Grounds RM, Rhodes A, Cecconi M: **Cardiac complications associated with goal-directed therapy in high-risk surgical patients: a meta-analysis**. *Br J Anaesth* 2014, **112**(4):648-659.

46. Arzola C, Wieczorek PM: **Efficacy of low-dose bupivacaine in spinal anaesthesia for Caesarean delivery: systematic review and meta-analysis**. *Br J Anaesth* 2011, **107**(3):308-318.

47. Assouline B, Tramer MR, Kreienbuhl L, Elia N: **Benefit and harm of adding ketamine to an opioid in a patient-controlled analgesia device for the control of postoperative pain: systematic review and meta-analyses of randomized controlled trials with trial sequential analyses**. *Pain* 2016, **157**(12):2854-2864.

48. Aya HD, Cecconi M, Hamilton M, Rhodes A: **Goal-directed therapy in cardiac surgery: a systematic review and meta-analysis**. *Br J Anaesth* 2013, **110**(4):510-517.

49. Badenes R, Bilotta F: **Neurocritical care for intracranial haemorrhage: a systematic review of recent studies**. *Br J Anaesth* 2015, **115 Suppl 2**:ii68-74.

50. Badgett RG, Lawrence VA, Cohn SL: **Variations in pharmacology of beta-blockers may contribute to heterogeneous results in trials of perioperative beta-blockade**. *Anesthesiology* 2010, **113**(3):585-592.

51. Baekgaard JS, Eskesen TG, Sillesen M, Rasmussen LS, Steinmetz J: **Ketamine as a Rapid Sequence Induction Agent in the Trauma Population: A Systematic Review**. *Anesth Analg* 2019, **128**(3):504-510.

52. Baeriswyl M, Kirkham KR, Jacot-Guillarmod A, Albrecht E: **Efficacy of perineural vs systemic dexamethasone to prolong analgesia after peripheral nerve block: a systematic review and meta-analysis**. *Br J Anaesth* 2017, **119**(2):183-191.

53. Baeriswyl M, Kirkham KR, Kern C, Albrecht E: **The Analgesic Efficacy of Ultrasound-Guided Transversus Abdominis Plane Block in Adult Patients: A Meta-Analysis**. *Anesth Analg* 2015, **121**(6):1640-1654.

54. Baidya DK, Chandralekha, Darlong V, Pandey R, Maitra S, Khanna P: **Comparative efficacy and safety of the Ambu((R)) AuraOnce() laryngeal mask airway during general anaesthesia in adults: a systematic review and meta-analysis**. *Anaesthesia* 2014, **69**(9):1023-1032.

55. Bailey M, Corcoran T, Schug S, Toner A: **Perioperative lidocaine infusions for the prevention of chronic postsurgical pain: a systematic review and meta-analysis of efficacy and safety**. *Pain* 2018, **159**(9):1696-1704.

56. Barile L, Fominskiy E, Di Tomasso N, Alpizar Castro LE, Landoni G, De Luca M, Bignami E, Sala A, Zangrillo A, Monaco F: **Acute Normovolemic Hemodilution Reduces Allogeneic Red Blood Cell Transfusion in Cardiac Surgery: A Systematic Review and Meta-analysis of Randomized Trials**. *Anesth Analg* 2017, **124**(3):743-752.

57. Barnett SF, Alagar RK, Grocott MP, Giannaris S, Dick JR, Moonesinghe SR: **Patient-satisfaction measures in anesthesia: qualitative systematic review**. *Anesthesiology* 2013, **119**(2):452-478.

58. Barreveld A, Witte J, Chahal H, Durieux ME, Strichartz G: **Preventive analgesia by local anesthetics: the reduction of postoperative pain by peripheral nerve blocks and intravenous drugs**. *Anesth Analg* 2013, **116**(5):1141-1161.

59. Barry AE, Chaney MA, London MJ: **Anesthetic management during cardiopulmonary bypass: a systematic review**. *Anesth Analg* 2015, **120**(4):749-769.

60. Basu SM, Chung FF, AbdelHakim SF, Wong J: **Anesthetic Considerations for Patients With Congenital Central Hypoventilation Syndrome: A Systematic Review of the Literature**. *Anesth Analg* 2017, **124**(1):169-178.

61. Beattie WS, Wijeysundera DN, Karkouti K, McCluskey S, Tait G: **Does tight heart rate control improve beta-blocker efficacy? An updated analysis of the noncardiac surgical randomized trials**. *Anesth Analg* 2008, **106**(4):1039-1048, table of contents.

62. Becker WC, Fraenkel L, Edelman EJ, Holt SR, Glover J, Kerns RD, Fiellin DA: **Instruments to assess patient-reported safety, efficacy, or misuse of current opioid therapy for chronic pain: a systematic review**. *Pain* 2013, **154**(6):905-916.

63. Beerthuizen A, van 't Spijker A, Huygen FJ, Klein J, de Wit R: **Is there an association between psychological factors and the Complex Regional Pain Syndrome type 1 (CRPS1) in adults? A systematic review**. *Pain* 2009, **145**(1-2):52-59.

64. Belletti A, Castro ML, Silvetti S, Greco T, Biondi-Zoccai G, Pasin L, Zangrillo A, Landoni G: **The Effect of inotropes and vasopressors on mortality: a meta-analysis of randomized clinical trials**. *Br J Anaesth* 2015, **115**(5):656-675.

65. Bellis JR, Pirmohamed M, Nunn AJ, Loke YK, De S, Golder S, Kirkham JJ: **Dexamethasone and haemorrhage risk in paediatric tonsillectomy: a systematic review and meta-analysis**. *Br J Anaesth* 2014, **113**(1):23-42.

66. Bender JL, Radhakrishnan A, Diorio C, Englesakis M, Jadad AR: **Can pain be managed through the Internet? A systematic review of randomized controlled trials**. *Pain* 2011, **152**(8):1740-1750.

67. Bennett MI, Bagnall AM, Jose Closs S: **How effective are patient-based educational interventions in the management of cancer pain? Systematic review and meta-analysis**. *Pain* 2009, **143**(3):192-199.

68. Bennett MI, Rayment C, Hjermstad M, Aass N, Caraceni A, Kaasa S: **Prevalence and aetiology of neuropathic pain in cancer patients: a systematic review**. *Pain* 2012, **153**(2):359-365.

69. Benyamin RM, Manchikanti L, Parr AT, Diwan S, Singh V, Falco FJ, Datta S, Abdi S, Hirsch JA: **The effectiveness of lumbar interlaminar epidural injections in managing chronic low back and lower extremity pain**. *Pain Physician* 2012, **15**(4):E363-404.

70. Benyamin RM, Singh V, Parr AT, Conn A, Diwan S, Abdi S: **Systematic review of the effectiveness of cervical epidurals in the management of chronic neck pain**. *Pain Physician* 2009, **12**(1):137-157.

71. Benyamin RM, Wang VC, Vallejo R, Singh V, Helm Ii S: **A systematic evaluation of thoracic interlaminar epidural injections**. *Pain Physician* 2012, **15**(4):E497-514.

72. Berryman C, Stanton TR, Jane Bowering K, Tabor A, McFarlane A, Lorimer Moseley G: **Evidence for working memory deficits in chronic pain: a systematic review and meta-analysis**. *Pain* 2013, **154**(8):1181-1196.

73. Bhatia A, Brull R: **Review article: is ultrasound guidance advantageous for interventional pain management? A systematic review of chronic pain outcomes**. *Anesth Analg* 2013, **117**(1):236-251.

74. Bhatia A, Flamer D, Shah PS, Cohen SP: **Transforaminal Epidural Steroid Injections for Treating Lumbosacral Radicular Pain from Herniated Intervertebral Discs: A Systematic Review and Meta-Analysis**. *Anesth Analg* 2016, **122**(3):857-870.

75. Biccard BM, Goga S, de Beurs J: **Dexmedetomidine and cardiac protection for non-cardiac surgery: a meta-analysis of randomised controlled trials**. *Anaesthesia* 2008, **63**(1):4-14.

76. Biccard BM, Rodseth RN: **A meta-analysis of the prospective randomised trials of coronary revascularisation before noncardiac vascular surgery with attention to the type of coronary revascularisation performed**. *Anaesthesia* 2009, **64**(10):1105-1113.

77. Biccard BM, Sear JW, Foex P: **Meta-analysis of the effect of heart rate achieved by perioperative beta-adrenergic blockade on cardiovascular outcomes**. *Br J Anaesth* 2008, **100**(1):23-28.

78. Bicket MC, Gupta A, Brown CHt, Cohen SP: **Epidural injections for spinal pain: a systematic review and meta-analysis evaluating the "control" injections in randomized controlled trials**. *Anesthesiology* 2013, **119**(4):907-931.

79. Biddiss E, Knibbe TJ, McPherson A: **The effectiveness of interventions aimed at reducing anxiety in health care waiting spaces: a systematic review of randomized and nonrandomized trials**. *Anesth Analg* 2014, **119**(2):433-448.

80. Bilotta F, Gelb AW, Stazi E, Titi L, Paoloni FP, Rosa G: **Pharmacological perioperative brain neuroprotection: a qualitative review of randomized clinical trials**. *Br J Anaesth* 2013, **110 Suppl 1**:i113-120.

81. Bingham AE, Fu R, Horn JL, Abrahams MS: **Continuous peripheral nerve block compared with single-injection peripheral nerve block: a systematic review and meta-analysis of randomized controlled trials**. *Reg Anesth Pain Med* 2012, **37**(6):583-594.

82. Bishop A, Thomas E, Foster NE: **Health care practitioners' attitudes and beliefs about low back pain: a systematic search and critical review of available measurement tools**. *Pain* 2007, **132**(1-2):91-101.

83. Blaudszun G, Lysakowski C, Elia N, Tramer MR: **Effect of perioperative systemic alpha2 agonists on postoperative morphine consumption and pain intensity: systematic review and meta-analysis of randomized controlled trials**. *Anesthesiology* 2012, **116**(6):1312-1322.

84. Boddy AP, Mehta S, Rhodes M: **The effect of intraperitoneal local anesthesia in laparoscopic cholecystectomy: a systematic review and meta-analysis**. *Anesth Analg* 2006, **103**(3):682-688.

85. Boerner KE, Birnie KA, Caes L, Schinkel M, Chambers CT: **Sex differences in experimental pain among healthy children: a systematic review and meta-analysis**. *Pain* 2014, **155**(5):983-993.

86. Boerner KE, Eccleston C, Chambers CT, Keogh E: **Sex differences in the efficacy of psychological therapies for the management of chronic and recurrent pain in children and adolescents: A systematic review and meta-analysis**. *Pain* 2017, **258**(4):569-582.

87. Boesch E, Bellan V, Moseley GL, Stanton TR: **The effect of bodily illusions on clinical pain: a systematic review and meta-analysis**. *Pain* 2016, **157**(3):516-529.

88. Boland JW, Ziegler L, Boland EG, McDermid K, Bennett MI: **Is regular systemic opioid analgesia associated with shorter survival in adult patients with cancer? A systematic literature review**. *Pain* 2015, **156**(11):2152-2163.

89. Bolton CM, Myles PS, Nolan T, Sterne JA: **Prophylaxis of postoperative vomiting in children undergoing tonsillectomy: a systematic review and meta-analysis**. *Br J Anaesth* 2006, **97**(5):593-604.

90. Bonnet MP, Marret E, Josserand J, Mercier FJ: **Effect of prophylactic 5-HT3 receptor antagonists on pruritus induced by neuraxial opioids: a quantitative systematic review**. *Br J Anaesth* 2008, **101**(3):311-319.

91. Borjesson M, Andrell P, Lundberg D, Mannheimer C: **Spinal cord stimulation in severe angina pectoris--a systematic review based on the Swedish Council on Technology assessment in health care report on long-standing pain**. *Pain* 2008, **140**(3):501-508.

92. Boswell MV, Colson JD, Sehgal N, Dunbar EE, Epter R: **A systematic review of therapeutic facet joint interventions in chronic spinal pain**. *Pain Physician* 2007, **10**(1):229-253.

93. Braz JRC, Braz MG, Hayashi Y, Martins RHG, Betini M, Braz LG, El Dib R: **Effects of different fresh gas flows with or without a heat and moisture exchanger on inhaled gas humidity in adults undergoing general anaesthesia: A systematic review and meta-analysis of randomised controlled trials**. *Eur J Anaesthesiol* 2017, **34**(8):515-525.

94. Brogi E, Cyr S, Kazan R, Giunta F, Hemmerling TM: **Clinical Performance and Safety of Closed-Loop Systems: A Systematic Review and Meta-analysis of Randomized Controlled Trials**. *Anesth Analg* 2017, **124**(2):446-455.

95. Bruintjes MH, van Helden EV, Braat AE, Dahan A, Scheffer GJ, van Laarhoven CJ, Warle MC: **Deep neuromuscular block to optimize surgical space conditions during laparoscopic surgery: a systematic review and meta-analysis**. *Br J Anaesth* 2017, **118**(6):834-842.

96. Bruls VE, Bastiaenen CH, de Bie RA: **Prognostic factors of complaints of arm, neck, and/or shoulder: a systematic review of prospective cohort studies**. *Pain* 2015, **156**(5):765-788.

97. Buenaventura RM, Datta S, Abdi S, Smith HS: **Systematic review of therapeutic lumbar transforaminal epidural steroid injections**. *Pain Physician* 2009, **12**(1):233-251.

98. Buggy DJ, Freeman J, Johnson MZ, Leslie K, Riedel B, Sessler DI, Kurz A, Gottumukkala V, Short T, Pace N *et al*: **Systematic review and consensus definitions for standardised endpoints in perioperative medicine: postoperative cancer outcomes**. *Br J Anaesth* 2018, **121**(1):38-44.

99. Cabrini L, Nobile L, Plumari VP, Landoni G, Borghi G, Mucchetti M, Zangrillo A: **Intraoperative prophylactic and therapeutic non-invasive ventilation: a systematic review**. *Br J Anaesth* 2014, **112**(4):638-647.

100. Cai GH, Huang J, Zhao Y, Chen J, Wu HH, Dong YL, Smith HS, Li YQ, Wang W, Wu SX: **Antioxidant therapy for pain relief in patients with chronic pancreatitis: systematic review and meta-analysis**. *Pain Physician* 2013, **16**(6):521-532.

101. Cao Y, Zheng OJ: **Tonabersat for migraine prophylaxis: a systematic review**. *Pain Physician* 2014, **17**(1):1-8.

102. Carlisle JB: **A meta-analysis of prevention of postoperative nausea and vomiting: randomised controlled trials by Fujii et al. compared with other authors**. *Anaesthesia* 2012, **67**(10):1076-1090.

103. Cata JP, Guerra CE, Chang GJ, Gottumukkala V, Joshi GP: **Non-steroidal anti-inflammatory drugs in the oncological surgical population: beneficial or harmful? A systematic review of the literature**. *Br J Anaesth* 2017, **119**(4):750-764.

104. Chambers D, Paulden M, Paton F, Heirs M, Duffy S, Hunter JM, Sculpher M, Woolacott N: **Sugammadex for reversal of neuromuscular block after rapid sequence intubation: a systematic review and economic assessment**. *Br J Anaesth* 2010, **105**(5):568-575.

105. Chamorro C, Borrallo JM, Romera MA, Silva JA, Balandin B: **Anesthesia and analgesia protocol during therapeutic hypothermia after cardiac arrest: a systematic review**. *Anesth Analg* 2010, **110**(5):1328-1335.

106. Chang-Chien GC, Knezevic NN, McCormick Z, Chu SK, Trescot AM, Candido KD: **Transforaminal versus interlaminar approaches to epidural steroid injections: a systematic review of comparative studies for lumbosacral radicular pain**. *Pain Physician* 2014, **17**(4):E509-524.

107. Chanthong P, Abrishami A, Wong J, Herrera F, Chung F: **Systematic review of questionnaires measuring patient satisfaction in ambulatory anesthesia**. *Anesthesiology* 2009, **110**(5):1061-1067.

108. Chao I, Young J, Coles-Black J, Chuen J, Weinberg L, Rachbuch C: **The application of three-dimensional printing technology in anaesthesia: a systematic review**. *Anaesthesia* 2017, **72**(5):641-650.

109. Chazapis M, Gilhooly D, Smith AF, Myles PS, Haller G, Grocott MPW, Moonesinghe SR: **Perioperative structure and process quality and safety indicators: a systematic review**. *Br J Anaesth* 2018, **120**(1):51-66.

110. Chebbout R, Heywood EG, Drake TM, Wild JRL, Lee J, Wilson M, Lee MJ: **A systematic review of the incidence of and risk factors for postoperative atrial fibrillation following general surgery**. *Anaesthesia* 2018, **73**(4):490-498.

111. Chen XX, Trivedi V, AlSaflan AA, Todd SC, Tricco AC, McCartney CJL, Boet S: **Ultrasound-Guided Regional Anesthesia Simulation Training: A Systematic Review**. *Reg Anesth Pain Med* 2017, **42**(6):741-750.

112. Cheng CR, Sessler DI, Apfel CC: **Does neostigmine administration produce a clinically important increase in postoperative nausea and vomiting?** *Anesth Analg* 2005, **101**(5):1349-1355.

113. Choi GJ, Kang H, Baek CW, Jung YH, Woo YC, Cha YJ: **A systematic review and meta-analysis of the i-gel(R) vs laryngeal mask airway in children**. *Anaesthesia* 2014, **69**(11):1258-1265.

114. Choi GJ, Kang H, Baek CW, Jung YH, Woo YC, Kim SH, Kim JG: **Comparison of streamlined liner of the pharynx airway (SLIPA ) and laryngeal mask airway: a systematic review and meta-analysis**. *Anaesthesia* 2015, **70**(5):613-622.

115. Choi S, Brull R: **Neuraxial techniques in obstetric and non-obstetric patients with common bleeding diatheses**. *Anesth Analg* 2009, **109**(2):648-660.

116. Choi S, Brull R: **Is ultrasound guidance advantageous for interventional pain management? A review of acute pain outcomes**. *Anesth Analg* 2011, **113**(3):596-604.

117. Choi S, Rodseth R, McCartney CJ: **Effects of dexamethasone as a local anaesthetic adjuvant for brachial plexus block: a systematic review and meta-analysis of randomized trials**. *Br J Anaesth* 2014, **112**(3):427-439.

118. Chong MA, Berbenetz NM, Lin C, Singh S: **Perineural Versus Intravenous Dexamethasone as an Adjuvant for Peripheral Nerve Blocks: A Systematic Review and Meta-Analysis**. *Reg Anesth Pain Med* 2017, **42**(3):319-326.

119. Chong MA, Szoke DJ, Berbenetz NM, Lin C: **Dexamethasone as an Adjuvant for Caudal Blockade in Pediatric Surgical Patients: A Systematic Review and Meta-analysis**. *Anesth Analg* 2018, **127**(2):520-528.

120. Chong MA, Wang Y, Berbenetz NM, McConachie I: **Does goal-directed haemodynamic and fluid therapy improve peri-operative outcomes?: A systematic review and meta-analysis**. *Eur J Anaesthesiol* 2018, **35**(7):469-483.

121. Chong SW, Peyton PJ: **A meta-analysis of the accuracy and precision of the ultrasonic cardiac output monitor (USCOM)**. *Anaesthesia* 2012, **67**(11):1266-1271.

122. Chung JW, Zeng Y, Wong TK: **Drug therapy for the treatment of chronic nonspecific low back pain: systematic review and meta-analysis**. *Pain Physician* 2013, **16**(6):E685-704.

123. Chung SA, Yuan H, Chung F: **A systemic review of obstructive sleep apnea and its implications for anesthesiologists**. *Anesth Analg* 2008, **107**(5):1543-1563.

124. Clark K, Lam LT, Gibson S, Currow D: **The effect of ranitidine versus proton pump inhibitors on gastric secretions: a meta-analysis of randomised control trials**. *Anaesthesia* 2009, **64**(6):652-657.

125. Clarke H, Bonin RP, Orser BA, Englesakis M, Wijeysundera DN, Katz J: **The prevention of chronic postsurgical pain using gabapentin and pregabalin: a combined systematic review and meta-analysis**. *Anesth Analg* 2012, **115**(2):428-442.

126. Claudius C, Viby-Mogensen J: **Acceleromyography for use in scientific and clinical practice: a systematic review of the evidence**. *Anesthesiology* 2008, **108**(6):1117-1140.

127. Clausen NG, Kahler S, Hansen TG: **Systematic review of the neurocognitive outcomes used in studies of paediatric anaesthesia neurotoxicity**. *Br J Anaesth* 2018, **120**(6):1255-1273.

128. Cohen B, Schacham YN, Ruetzler K, Ahuja S, Yang D, Mascha EJ, Barclay AB, Hung MH, Sessler DI: **Effect of intraoperative hyperoxia on the incidence of surgical site infections: a meta-analysis**. *Br J Anaesth* 2018, **120**(6):1176-1186.

129. Cohen SP, Kapoor SG, Rathmell JP: **Intravenous infusion tests have limited utility for selecting long-term drug therapy in patients with chronic pain: a systematic review**. *Anesthesiology* 2009, **111**(2):416-431.

130. Colebourn CL, Barber V, Young JD: **Use of helium-oxygen mixture in adult patients presenting with exacerbations of asthma and chronic obstructive pulmonary disease: a systematic review**. *Anaesthesia* 2007, **62**(1):34-42.

131. Colson J, Koyyalagunta D, Falco FJ, Manchikanti L: **A systematic review of observational studies on the effectiveness of opioid therapy for cancer pain**. *Pain Physician* 2011, **14**(2):E85-102.

132. Conn A, Buenaventura RM, Datta S, Abdi S, Diwan S: **Systematic review of caudal epidural injections in the management of chronic low back pain**. *Pain Physician* 2009, **12**(1):109-135.

133. Conway A, Douglas C, Sutherland JR: **A systematic review of capnography for sedation**. *Anaesthesia* 2016, **71**(4):450-454.

134. Cooke K, Sharvill R, Sondergaard S, Aneman A: **Volume responsiveness assessed by passive leg raising and a fluid challenge: a critical review focused on mean systemic filling pressure**. *Anaesthesia* 2018, **73**(3):313-322.

135. Corcoran T, Rhodes JE, Clarke S, Myles PS, Ho KM: **Perioperative fluid management strategies in major surgery: a stratified meta-analysis**. *Anesth Analg* 2012, **114**(3):640-651.

136. Cornelius VR, Sauzet O, Williams JE, Ayis S, Farquhar-Smith P, Ross JR, Branford RA, Peacock JL: **Adverse event reporting in randomised controlled trials of neuropathic pain: considerations for future practice**. *Pain* 2013, **154**(2):213-220.

137. Correa D, Farney RJ, Chung F, Prasad A, Lam D, Wong J: **Chronic opioid use and central sleep apnea: a review of the prevalence, mechanisms, and perioperative considerations**. *Anesth Analg* 2015, **120**(6):1273-1285.

138. Corredor C, Wasowicz M, Karkouti K, Sharma V: **The role of point-of-care platelet function testing in predicting postoperative bleeding following cardiac surgery: a systematic review and meta-analysis**. *Anaesthesia* 2015, **70**(6):715-731.

139. Cozowicz C, Chung F, Doufas AG, Nagappa M, Memtsoudis SG: **Opioids for Acute Pain Management in Patients With Obstructive Sleep Apnea: A Systematic Review**. *Anesth Analg* 2018, **127**(4):988-1001.

140. Cragg JJ, Warner FM, Finnerup NB, Jensen MP, Mercier C, Richards JS, Wrigley P, Soler D, Kramer JL: **Meta-analysis of placebo responses in central neuropathic pain: impact of subject, study, and pain characteristics**. *Pain* 2016, **157**(3):530-540.

141. Crawford CC, Huynh MT, Kepple A, Jonas WB: **Systematic assessment of the quality of research studies of conventional and alternative treatment(s) of primary headache**. *Pain Physician* 2009, **12**(2):461-470.

142. Crellin DJ, Harrison D, Santamaria N, Babl FE: **Systematic review of the Face, Legs, Activity, Cry and Consolability scale for assessing pain in infants and children: is it reliable, valid, and feasible for use?** *Pain* 2015, **156**(11):2132-2151.

143. Crescenzi G, Landoni G, Biondi-Zoccai G, Pappalardo F, Nuzzi M, Bignami E, Fochi O, Maj G, Calabro MG, Ranucci M *et al*: **Desmopressin reduces transfusion needs after surgery: a meta-analysis of randomized clinical trials**. *Anesthesiology* 2008, **109**(6):1063-1076.

144. Crombez G, Beirens K, Van Damme S, Eccleston C, Fontaine J: **The unbearable lightness of somatisation: a systematic review of the concept of somatisation in empirical studies of pain**. *Pain* 2009, **145**(1-2):31-35.

145. Crombez G, Van Ryckeghem DM, Eccleston C, Van Damme S: **Attentional bias to pain-related information: a meta-analysis**. *Pain* 2013, **154**(4):497-510.

146. Currie GL, Delaney A, Bennett MI, Dickenson AH, Egan KJ, Vesterinen HM, Sena ES, Macleod MR, Colvin LA, Fallon MT: **Animal models of bone cancer pain: systematic review and meta-analyses**. *Pain* 2013, **154**(6):917-926.

147. da Silva PS, Fonseca MC: **Unplanned endotracheal extubations in the intensive care unit: systematic review, critical appraisal, and evidence-based recommendations**. *Anesth Analg* 2012, **114**(5):1003-1014.

148. Dahmani S, Stany I, Brasher C, Lejeune C, Bruneau B, Wood C, Nivoche Y, Constant I, Murat I: **Pharmacological prevention of sevoflurane- and desflurane-related emergence agitation in children: a meta-analysis of published studies**. *Br J Anaesth* 2010, **104**(2):216-223.

149. Dai Y, Lee A, Critchley LA, White PF: **Does thromboelastography predict postoperative thromboembolic events? A systematic review of the literature**. *Anesth Analg* 2009, **108**(3):734-742.

150. Dale O, Somogyi AA, Li Y, Sullivan T, Shavit Y: **Does intraoperative ketamine attenuate inflammatory reactivity following surgery? A systematic review and meta-analysis**. *Anesth Analg* 2012, **115**(4):934-943.

151. Davidson F, Snow S, Hayden JA, Chorney J: **Psychological interventions in managing postoperative pain in children: a systematic review**. *Pain* 2016, **157**(9):1872-1886.

152. Davies RG, Myles PS, Graham JM: **A comparison of the analgesic efficacy and side-effects of paravertebral vs epidural blockade for thoracotomy--a systematic review and meta-analysis of randomized trials**. *Br J Anaesth* 2006, **96**(4):418-426.

153. de Montblanc J, Ruscio L, Mazoit JX, Benhamou D: **A systematic review and meta-analysis of the i-gel((R)) vs laryngeal mask airway in adults**. *Anaesthesia* 2014, **69**(10):1151-1162.

154. De Oliveira GS, Jr., Agarwal D, Benzon HT: **Perioperative single dose ketorolac to prevent postoperative pain: a meta-analysis of randomized trials**. *Anesth Analg* 2012, **114**(2):424-433.

155. De Oliveira GS, Jr., Almeida MD, Benzon HT, McCarthy RJ: **Perioperative single dose systemic dexamethasone for postoperative pain: a meta-analysis of randomized controlled trials**. *Anesthesiology* 2011, **115**(3):575-588.

156. de Oliveira GS, Jr., Balliu B, Nader A, McCarthy RJ: **Dose-ranging effects of intrathecal epinephrine on anesthesia/analgesia: a meta-analysis and metaregression of randomized controlled trials**. *Reg Anesth Pain Med* 2012, **37**(4):423-432.

157. De Oliveira GS, Jr., Castro-Alves LJ, Ahmad S, Kendall MC, McCarthy RJ: **Dexamethasone to prevent postoperative nausea and vomiting: an updated meta-analysis of randomized controlled trials**. *Anesth Analg* 2013, **116**(1):58-74.

158. De Oliveira GS, Jr., Castro-Alves LJ, Chang R, Yaghmour E, McCarthy RJ: **Systemic metoclopramide to prevent postoperative nausea and vomiting: a meta-analysis without Fujii's studies**. *Br J Anaesth* 2012, **109**(5):688-697.

159. De Oliveira GS, Jr., Castro-Alves LJ, Khan JH, McCarthy RJ: **Perioperative systemic magnesium to minimize postoperative pain: a meta-analysis of randomized controlled trials**. *Anesthesiology* 2013, **119**(1):178-190.

160. De Oliveira GS, Jr., Castro-Alves LJ, Nader A, Kendall MC, McCarthy RJ: **Transversus abdominis plane block to ameliorate postoperative pain outcomes after laparoscopic surgery: a meta-analysis of randomized controlled trials**. *Anesth Analg* 2014, **118**(2):454-463.

161. de Waal BA, Buise MP, van Zundert AA: **Perioperative statin therapy in patients at high risk for cardiovascular morbidity undergoing surgery: a review**. *Br J Anaesth* 2015, **114**(1):44-52.

162. Devulder J, Jacobs A, Richarz U, Wiggett H: **Impact of opioid rescue medication for breakthrough pain on the efficacy and tolerability of long-acting opioids in patients with chronic non-malignant pain**. *Br J Anaesth* 2009, **103**(4):576-585.

163. Dexter F, Bayman EO, Epstein RH: **Statistical modeling of average and variability of time to extubation for meta-analysis comparing desflurane to sevoflurane**. *Anesth Analg* 2010, **110**(2):570-580.

164. Dexter F, Dexter EU, Masursky D, Nussmeier NA: **Systematic review of general thoracic surgery articles to identify predictors of operating room case durations**. *Anesth Analg* 2008, **106**(4):1232-1241, table of contents.

165. Dieleman JP, Kerklaan J, Huygen FJ, Bouma PA, Sturkenboom MC: **Incidence rates and treatment of neuropathic pain conditions in the general population**. *Pain* 2008, **137**(3):681-688.

166. Ditre JW, Heckman BW, Zale EL, Kosiba JD, Maisto SA: **Acute analgesic effects of nicotine and tobacco in humans: a meta-analysis**. *Pain* 2016, **157**(7):1373-1381.

167. Diwan S, Manchikanti L, Benyamin RM, Bryce DA, Geffert S, Hameed H, Sharma ML, Abdi S, Falco FJ: **Effectiveness of cervical epidural injections in the management of chronic neck and upper extremity pain**. *Pain Physician* 2012, **15**(4):E405-434.

168. Doleman B, Heinink TP, Read DJ, Faleiro RJ, Lund JN, Williams JP: **A systematic review and meta-regression analysis of prophylactic gabapentin for postoperative pain**. *Anaesthesia* 2015, **70**(10):1186-1204.

169. Doleman B, Read D, Lund JN, Williams JP: **Preventive Acetaminophen Reduces Postoperative Opioid Consumption, Vomiting, and Pain Scores After Surgery: Systematic Review and Meta-Analysis**. *Reg Anesth Pain Med* 2015, **40**(6):706-712.

170. Doleman B, Sutton AJ, Sherwin M, Lund JN, Williams JP: **Baseline Morphine Consumption May Explain Between-Study Heterogeneity in Meta-analyses of Adjuvant Analgesics and Improve Precision and Accuracy of Effect Estimates**. *Anesth Analg* 2018, **126**(2):648-660.

171. Doth AH, Hansson PT, Jensen MP, Taylor RS: **The burden of neuropathic pain: a systematic review and meta-analysis of health utilities**. *Pain* 2010, **149**(2):338-344.

172. Ducasse D, Courtet P, Olie E: **Burning mouth syndrome: current clinical, physiopathologic, and therapeutic data**. *Reg Anesth Pain Med* 2013, **38**(5):380-390.

173. Duggan LV, Ballantyne Scott B, Law JA, Morris IR, Murphy MF, Griesdale DE: **Transtracheal jet ventilation in the 'can't intubate can't oxygenate' emergency: a systematic review**. *Br J Anaesth* 2016, **117 Suppl 1**:i28-i38.

174. Dupuis S, Amiel JA, Desgroseilliers M, Williamson DR, Thiboutot Z, Serri K, Perreault MM, Marsolais P, Frenette AJ: **Corticosteroids in the management of brain-dead potential organ donors: a systematic review**. *Br J Anaesth* 2014, **113**(3):346-359.

175. Dvirnik N, Belley-Cote EP, Hanif H, Devereaux PJ, Lamy A, Dieleman JM, Vincent J, Whitlock RP: **Steroids in cardiac surgery: a systematic review and meta-analysis**. *Br J Anaesth* 2018, **120**(4):657-667.

176. Dworkin RH, O'Connor AB, Backonja M, Farrar JT, Finnerup NB, Jensen TS, Kalso EA, Loeser JD, Miaskowski C, Nurmikko TJ *et al*: **Pharmacologic management of neuropathic pain: evidence-based recommendations**. *Pain* 2007, **132**(3):237-251.

177. Dworkin RH, O'Connor AB, Kent J, Mackey SC, Raja SN, Stacey BR, Levy RM, Backonja M, Baron R, Harke H *et al*: **Interventional management of neuropathic pain: NeuPSIG recommendations**. *Pain* 2013, **154**(11):2249-2261.

178. Eccleston C, Morley SJ, Williams AC: **Psychological approaches to chronic pain management: evidence and challenges**. *Br J Anaesth* 2013, **111**(1):59-63.

179. Egal M, de Geus HR, van Bommel J, Groeneveld AB: **Targeting oliguria reversal in perioperative restrictive fluid management does not influence the occurrence of renal dysfunction: A systematic review and meta-analysis**. *Eur J Anaesthesiol* 2016, **33**(6):425-435.

180. Egal M, Erler NS, de Geus HR, van Bommel J, Groeneveld AB: **Targeting Oliguria Reversal in Goal-Directed Hemodynamic Management Does Not Reduce Renal Dysfunction in Perioperative and Critically Ill Patients: A Systematic Review and Meta-Analysis**. *Anesth Analg* 2016, **122**(1):173-185.

181. Eichen PM, Achilles N, Konig V, Mosges R, Hellmich M, Himpe B, Kirchner R: **Nucleoplasty, a minimally invasive procedure for disc decompression: a systematic review and meta-analysis of published clinical studies**. *Pain Physician* 2014, **17**(2):E149-173.

182. Eipe N, Penning J, Yazdi F, Mallick R, Turner L, Ahmadzai N, Ansari MT: **Perioperative use of pregabalin for acute pain-a systematic review and meta-analysis**. *Pain* 2015, **156**(7):1284-1300.

183. El-Boghdadly K, Bailey CR, Wiles MD: **Postoperative sore throat: a systematic review**. *Anaesthesia* 2016, **71**(6):706-717.

184. El-Boghdadly K, Brull R, Sehmbi H, Abdallah FW: **Perineural Dexmedetomidine Is More Effective Than Clonidine When Added to Local Anesthetic for Supraclavicular Brachial Plexus Block: A Systematic Review and Meta-analysis**. *Anesth Analg* 2017, **124**(6):2008-2020.

185. El-Boghdadly K, Madjdpour C, Chin KJ: **Thoracic paravertebral blocks in abdominal surgery - a systematic review of randomized controlled trials**. *Br J Anaesth* 2016, **117**(3):297-308.

186. Elia N, Culebras X, Mazza C, Schiffer E, Tramer MR: **Clonidine as an adjuvant to intrathecal local anesthetics for surgery: systematic review of randomized trials**. *Reg Anesth Pain Med* 2008, **33**(2):159-167.

187. Elia N, Lysakowski C, Tramer MR: **Does multimodal analgesia with acetaminophen, nonsteroidal antiinflammatory drugs, or selective cyclooxygenase-2 inhibitors and patient-controlled analgesia morphine offer advantages over morphine alone? Meta-analyses of randomized trials**. *Anesthesiology* 2005, **103**(6):1296-1304.

188. Elmi-Sarabi M, Deschamps A, Delisle S, Ased H, Haddad F, Lamarche Y, Perrault LP, Lambert J, Turgeon AF, Denault AY: **Aerosolized Vasodilators for the Treatment of Pulmonary Hypertension in Cardiac Surgical Patients: A Systematic Review and Meta-analysis**. *Anesth Analg* 2017, **125**(2):393-402.

189. Engelman E, Marsala C: **Efficacy of adding clonidine to intrathecal morphine in acute postoperative pain: meta-analysis**. *Br J Anaesth* 2013, **110**(1):21-27.

190. Engelman E, Salengros JC, Barvais L: **How much does pharmacologic prophylaxis reduce postoperative vomiting in children? Calculation of prophylaxis effectiveness and expected incidence of vomiting under treatment using Bayesian meta-analysis**. *Anesthesiology* 2008, **109**(6):1023-1035.

191. Epter RS, Helm S, 2nd, Hayek SM, Benyamin RM, Smith HS, Abdi S: **Systematic review of percutaneous adhesiolysis and management of chronic low back pain in post lumbar surgery syndrome**. *Pain Physician* 2009, **12**(2):361-378.

192. Ernst E, Lee MS, Choi TY: **Acupuncture: does it alleviate pain and are there serious risks? A review of reviews**. *Pain* 2011, **152**(4):755-764.

193. Evans MS, Lysakowski C, Tramer MR: **Nefopam for the prevention of postoperative pain: quantitative systematic review**. *Br J Anaesth* 2008, **101**(5):610-617.

194. Fabritius ML, Strom C, Koyuncu S, Jaeger P, Petersen PL, Geisler A, Wetterslev J, Dahl JB, Mathiesen O: **Benefit and harm of pregabalin in acute pain treatment: a systematic review with meta-analyses and trial sequential analyses**. *Br J Anaesth* 2017, **119**(4):775-791.

195. Falco FJ, Manchikanti L, Datta S, Sehgal N, Geffert S, Onyewu O, Zhu J, Coubarous S, Hameed M, Ward SP *et al*: **An update of the effectiveness of therapeutic lumbar facet joint interventions**. *Pain Physician* 2012, **15**(6):E909-953.

196. Falco FJ, Manchikanti L, Datta S, Wargo BW, Geffert S, Bryce DA, Atluri S, Singh V, Benyamin RM, Sehgal N *et al*: **Systematic review of the therapeutic effectiveness of cervical facet joint interventions: an update**. *Pain Physician* 2012, **15**(6):E839-868.

197. Falco FJ, Patel VB, Hayek SM, Deer TR, Geffert S, Zhu J, Onyewu O, Coubarous S, Smith HS, Manchikanti L: **Intrathecal infusion systems for long-term management of chronic non-cancer pain: an update of assessment of evidence**. *Pain Physician* 2013, **16**(2 Suppl):SE185-216.

198. Faraoni D, Goobie SM: **The efficacy of antifibrinolytic drugs in children undergoing noncardiac surgery: a systematic review of the literature**. *Anesth Analg* 2014, **118**(3):628-636.

199. Fathi AR, Eshtehardi P, Meier B: **Patent foramen ovale and neurosurgery in sitting position: a systematic review**. *Br J Anaesth* 2009, **102**(5):588-596.

200. Fayad A, Ansari MT, Yang H, Ruddy T, Wells GA: **Perioperative Diastolic Dysfunction in Patients Undergoing Noncardiac Surgery Is an Independent Risk Factor for Cardiovascular Events: A Systematic Review and Meta-analysis**. *Anesthesiology* 2016, **125**(1):72-91.

201. Felden L, Walter C, Harder S, Treede RD, Kayser H, Drover D, Geisslinger G, Lotsch J: **Comparative clinical effects of hydromorphone and morphine: a meta-analysis**. *Br J Anaesth* 2011, **107**(3):319-328.

202. Fenton G, Morley S: **A tale of two RCTs: using randomized controlled trials to benchmark routine clinical (psychological) treatments for chronic pain**. *Pain* 2013, **154**(10):2108-2119.

203. Fernandez-Guisasola J, Gomez-Arnau JI, Cabrera Y, del Valle SG: **Association between nitrous oxide and the incidence of postoperative nausea and vomiting in adults: a systematic review and meta-analysis**. *Anaesthesia* 2010, **65**(4):379-387.

204. Finnerup NB, Otto M, McQuay HJ, Jensen TS, Sindrup SH: **Algorithm for neuropathic pain treatment: an evidence based proposal**. *Pain* 2005, **118**(3):289-305.

205. Fischer HB, Simanski CJ: **A procedure-specific systematic review and consensus recommendations for analgesia after total hip replacement**. *Anaesthesia* 2005, **60**(12):1189-1202.

206. Fischer HB, Simanski CJ, Sharp C, Bonnet F, Camu F, Neugebauer EA, Rawal N, Joshi GP, Schug SA, Kehlet H *et al*: **A procedure-specific systematic review and consensus recommendations for postoperative analgesia following total knee arthroplasty**. *Anaesthesia* 2008, **63**(10):1105-1123.

207. Fitzsimmons D, Phillips CJ, Bennett H, Jones M, Williams N, Lewis R, Sutton A, Matar HE, Din N, Burton K *et al*: **Cost-effectiveness of different strategies to manage patients with sciatica**. *Pain* 2014, **155**(7):1318-1327.

208. Fletcher D, Martinez V: **Opioid-induced hyperalgesia in patients after surgery: a systematic review and a meta-analysis**. *Br J Anaesth* 2014, **112**(6):991-1004.

209. Foley PL, Vesterinen HM, Laird BJ, Sena ES, Colvin LA, Chandran S, MacLeod MR, Fallon MT: **Prevalence and natural history of pain in adults with multiple sclerosis: systematic review and meta-analysis**. *Pain* 2013, **154**(5):632-642.

210. Fominskiy E, Putzu A, Monaco F, Scandroglio AM, Karaskov A, Galas FR, Hajjar LA, Zangrillo A, Landoni G: **Liberal transfusion strategy improves survival in perioperative but not in critically ill patients. A meta-analysis of randomised trials**. *Br J Anaesth* 2015, **115**(4):511-519.

211. Fong HK, Sands LP, Leung JM: **The role of postoperative analgesia in delirium and cognitive decline in elderly patients: a systematic review**. *Anesth Analg* 2006, **102**(4):1255-1266.

212. Forbes HJ, Thomas SL, Smeeth L, Clayton T, Farmer R, Bhaskaran K, Langan SM: **A systematic review and meta-analysis of risk factors for postherpetic neuralgia**. *Pain* 2016, **157**(1):30-54.

213. Fouladpour N, Jesudoss R, Bolden N, Shaman Z, Auckley D: **Perioperative Complications in Obstructive Sleep Apnea Patients Undergoing Surgery: A Review of the Legal Literature**. *Anesth Analg* 2016, **122**(1):145-151.

214. Fowler SJ, Symons J, Sabato S, Myles PS: **Epidural analgesia compared with peripheral nerve blockade after major knee surgery: a systematic review and meta-analysis of randomized trials**. *Br J Anaesth* 2008, **100**(2):154-164.

215. Frey ME, Manchikanti L, Benyamin RM, Schultz DM, Smith HS, Cohen SP: **Spinal cord stimulation for patients with failed back surgery syndrome: a systematic review**. *Pain Physician* 2009, **12**(2):379-397.

216. Fullen BM, Baxter GD, O'Donovan BG, Doody C, Daly L, Hurley DA: **Doctors' attitudes and beliefs regarding acute low back pain management: A systematic review**. *Pain* 2008, **136**(3):388-396.

217. Gan H, Cannesson M, Chandler JR, Ansermino JM: **Predicting fluid responsiveness in children: a systematic review**. *Anesth Analg* 2013, **117**(6):1380-1392.

218. Garcia JB, Hernandez-Castro JJ, Nunez RG, Pazos MA, Aguirre JO, Jreige A, Delgado W, Serpentegui M, Berenguel M, Cantemir C: **Prevalence of low back pain in Latin America: a systematic literature review**. *Pain Physician* 2014, **17**(5):379-391.

219. Garvey GP, Wasade VS, Murphy KE, Balki M: **Anesthetic and Obstetric Management of Syringomyelia During Labor and Delivery: A Case Series and Systematic Review**. *Anesth Analg* 2017, **125**(3):913-924.

220. Gattas DJ, Dan A, Myburgh J, Billot L, Lo S, Finfer S, Committee CM: **Fluid resuscitation with 6% hydroxyethyl starch (130/0.4) in acutely ill patients: an updated systematic review and meta-analysis**. *Anesth Analg* 2012, **114**(1):159-169.

221. Gehling M, Tryba M: **Risks and side-effects of intrathecal morphine combined with spinal anaesthesia: a meta-analysis**. *Anaesthesia* 2009, **64**(6):643-651.

222. Gelineau AM, King MR, Ladha KS, Burns SM, Houle T, Anderson TA: **Intraoperative Esmolol as an Adjunct for Perioperative Opioid and Postoperative Pain Reduction: A Systematic Review, Meta-analysis, and Meta-regression**. *Anesth Analg* 2018, **126**(3):1035-1049.

223. George RB, Allen TK, Habib AS: **Serotonin receptor antagonists for the prevention and treatment of pruritus, nausea, and vomiting in women undergoing cesarean delivery with intrathecal morphine: a systematic review and meta-analysis**. *Anesth Analg* 2009, **109**(1):174-182.

224. George RB, Allen TK, Habib AS: **Intermittent epidural bolus compared with continuous epidural infusions for labor analgesia: a systematic review and meta-analysis**. *Anesth Analg* 2013, **116**(1):133-144.

225. Georgiou AP, Manara AR: **Role of therapeutic hypothermia in improving outcome after traumatic brain injury: a systematic review**. *Br J Anaesth* 2013, **110**(3):357-367.

226. Gerges FJ, Lipsitz SR, Nedeljkovic SS: **A systematic review on the effectiveness of the Nucleoplasty procedure for discogenic pain**. *Pain Physician* 2010, **13**(2):117-132.

227. Giglio MT, Marucci M, Testini M, Brienza N: **Goal-directed haemodynamic therapy and gastrointestinal complications in major surgery: a meta-analysis of randomized controlled trials**. *Br J Anaesth* 2009, **103**(5):637-646.

228. Gill JB, Kuper M, Chin PC, Zhang Y, Schutt R, Jr.: **Comparing pain reduction following kyphoplasty and vertebroplasty for osteoporotic vertebral compression fractures**. *Pain Physician* 2007, **10**(4):583-590.

229. Gillespie BM, Chaboyer W, Thalib L, John M, Fairweather N, Slater K: **Effect of using a safety checklist on patient complications after surgery: a systematic review and meta-analysis**. *Anesthesiology* 2014, **120**(6):1380-1389.

230. Gillies MA, Habicher M, Jhanji S, Sander M, Mythen M, Hamilton M, Pearse RM: **Incidence of postoperative death and acute kidney injury associated with i.v. 6% hydroxyethyl starch use: systematic review and meta-analysis**. *Br J Anaesth* 2014, **112**(1):25-34.

231. Glombiewski JA, Sawyer AT, Gutermann J, Koenig K, Rief W, Hofmann SG: **Psychological treatments for fibromyalgia: a meta-analysis**. *Pain* 2010, **151**(2):280-295.

232. Glossop AJ, Shephard N, Bryden DC, Mills GH: **Non-invasive ventilation for weaning, avoiding reintubation after extubation and in the postoperative period: a meta-analysis**. *Br J Anaesth* 2012, **109**(3):305-314.

233. Goldberg RJ, Katz J: **A meta-analysis of the analgesic effects of omega-3 polyunsaturated fatty acid supplementation for inflammatory joint pain**. *Pain* 2007, **129**(1-2):210-223.

234. Gopal S, Jayakumar D, Nelson PN: **Meta-analysis on the effect of dopexamine on in-hospital mortality**. *Anaesthesia* 2009, **64**(6):589-594.

235. Gornall BF, Myles PS, Smith CL, Burke JA, Leslie K, Pereira MJ, Bost JE, Kluivers KB, Nilsson UG, Tanaka Y *et al*: **Measurement of quality of recovery using the QoR-40: a quantitative systematic review**. *Br J Anaesth* 2013, **111**(2):161-169.

236. Grant MC, Betz M, Hulse M, Zorrilla-Vaca A, Hobson D, Wick E, Wu CL: **The Effect of Preoperative Pregabalin on Postoperative Nausea and Vomiting: A Meta-analysis**. *Anesth Analg* 2016, **123**(5):1100-1107.

237. Grant MC, Kim J, Page AJ, Hobson D, Wick E, Wu CL: **The Effect of Intravenous Midazolam on Postoperative Nausea and Vomiting: A Meta-Analysis**. *Anesth Analg* 2016, **122**(3):656-663.

238. Grant MC, Lee H, Page AJ, Hobson D, Wick E, Wu CL: **The Effect of Preoperative Gabapentin on Postoperative Nausea and Vomiting: A Meta-Analysis**. *Anesth Analg* 2016, **122**(4):976-985.

239. Grape S, Kirkham KR, Baeriswyl M, Albrecht E: **The analgesic efficacy of sciatic nerve block in addition to femoral nerve block in patients undergoing total knee arthroplasty: a systematic review and meta-analysis**. *Anaesthesia* 2016, **71**(10):1198-1209.

240. Grape S, Usmanova I, Kirkham KR, Albrecht E: **Intravenous dexamethasone for prophylaxis of postoperative nausea and vomiting after administration of long-acting neuraxial opioids: a systematic review and meta-analysis**. *Anaesthesia* 2018, **73**(4):480-489.

241. Greco T, Calabro MG, Covello RD, Greco M, Pasin L, Morelli A, Landoni G, Zangrillo A: **A Bayesian network meta-analysis on the effect of inodilatory agents on mortality**. *Br J Anaesth* 2015, **114**(5):746-756.

242. Green SM, Mason KP, Krauss BS: **Pulmonary aspiration during procedural sedation: a comprehensive systematic review**. *Br J Anaesth* 2017, **118**(3):344-354.

243. Grocott MP, Dushianthan A, Hamilton MA, Mythen MG, Harrison D, Rowan K, Optimisation Systematic Review Steering G: **Perioperative increase in global blood flow to explicit defined goals and outcomes after surgery: a Cochrane Systematic Review**. *Br J Anaesth* 2013, **111**(4):535-548.

244. Guilfoyle MR, Helmy A, Duane D, Hutchinson PJ: **Regional scalp block for postcraniotomy analgesia: a systematic review and meta-analysis**. *Anesth Analg* 2013, **116**(5):1093-1102.

245. Guimaraes-Pereira L, Reis P, Abelha F, Azevedo LF, Castro-Lopes JM: **Persistent postoperative pain after cardiac surgery: a systematic review with meta-analysis regarding incidence and pain intensity**. *Pain* 2017, **158**(10):1869-1885.

246. Gurgel ST, do Nascimento P, Jr.: **Maintaining tissue perfusion in high-risk surgical patients: a systematic review of randomized clinical trials**. *Anesth Analg* 2011, **112**(6):1384-1391.

247. Hajibandeh S, Hajibandeh S, Antoniou SA, Torella F, Antoniou GA: **Effect of beta-blockers on perioperative outcomes in vascular and endovascular surgery: a systematic review and meta-analysis**. *Br J Anaesth* 2017, **118**(1):11-21.

248. Halladin NL, Zahle FV, Rosenberg J, Gogenur I: **Interventions to reduce tourniquet-related ischaemic damage in orthopaedic surgery: a qualitative systematic review of randomised trials**. *Anaesthesia* 2014, **69**(9):1033-1050.

249. Haller G, Stoelwinder J, Myles PS, McNeil J: **Quality and safety indicators in anesthesia: a systematic review**. *Anesthesiology* 2009, **110**(5):1158-1175.

250. Hamberg-van Reenen HH, Ariens GA, Blatter BM, van Mechelen W, Bongers PM: **A systematic review of the relation between physical capacity and future low back and neck/shoulder pain**. *Pain* 2007, **130**(1-2):93-107.

251. Hamill JK, Lyndon M, Liley A, Hill AG: **Where it hurts: a systematic review of pain-location tools for children**. *Pain* 2014, **155**(5):851-858.

252. Hamilton GM, Wheeler K, Di Michele J, Lalu MM, McIsaac DI: **A Systematic Review and Meta-analysis Examining the Impact of Incident Postoperative Delirium on Mortality**. *Anesthesiology* 2017, **127**(1):78-88.

253. Hamilton MA, Cecconi M, Rhodes A: **A systematic review and meta-analysis on the use of preemptive hemodynamic intervention to improve postoperative outcomes in moderate and high-risk surgical patients**. *Anesth Analg* 2011, **112**(6):1392-1402.

254. Hamunen K, Kontinen V: **Systematic review on analgesics given for pain following tonsillectomy in children**. *Pain* 2005, **117**(1-2):40-50.

255. Hanna MN, Elhassan A, Veloso PM, Lesley M, Lissauer J, Richman JM, Wu CL: **Efficacy of bicarbonate in decreasing pain on intradermal injection of local anesthetics: a meta-analysis**. *Reg Anesth Pain Med* 2009, **34**(2):122-125.

256. Hansen H, Manchikanti L, Simopoulos TT, Christo PJ, Gupta S, Smith HS, Hameed H, Cohen SP: **A systematic evaluation of the therapeutic effectiveness of sacroiliac joint interventions**. *Pain Physician* 2012, **15**(3):E247-278.

257. Hansen HC, McKenzie-Brown AM, Cohen SP, Swicegood JR, Colson JD, Manchikanti L: **Sacroiliac joint interventions: a systematic review**. *Pain Physician* 2007, **10**(1):165-184.

258. Hansen MS, Brennum J, Moltke FB, Dahl JB: **Pain treatment after craniotomy: where is the (procedure-specific) evidence? A qualitative systematic review**. *Eur J Anaesthesiol* 2011, **28**(12):821-829.

259. Haroutiunian S, Nikolajsen L, Finnerup NB, Jensen TS: **The neuropathic component in persistent postsurgical pain: a systematic literature review**. *Pain* 2013, **154**(1):95-102.

260. Hartog CS, Kohl M, Reinhart K: **A systematic review of third-generation hydroxyethyl starch (HES 130/0.4) in resuscitation: safety not adequately addressed**. *Anesth Analg* 2011, **112**(3):635-645.

261. Hastings S, Myles P, McIlroy D: **Aspirin and coronary artery surgery: a systematic review and meta-analysis**. *Br J Anaesth* 2015, **115**(3):376-385.

262. Hattler J, Klimek M, Rossaint R, Heesen M: **The Effect of Combined Spinal-Epidural Versus Epidural Analgesia in Laboring Women on Nonreassuring Fetal Heart Rate Tracings: Systematic Review and Meta-analysis**. *Anesth Analg* 2016, **123**(4):955-964.

263. Hauser W, Bartram-Wunn E, Bartram C, Reinecke H, Tolle T: **Systematic review: Placebo response in drug trials of fibromyalgia syndrome and painful peripheral diabetic neuropathy-magnitude and patient-related predictors**. *Pain* 2011, **152**(8):1709-1717.

264. Hauser W, Bernardy K, Uceyler N, Sommer C: **Treatment of fibromyalgia syndrome with gabapentin and pregabalin--a meta-analysis of randomized controlled trials**. *Pain* 2009, **145**(1-2):69-81.

265. Hayek SM, Deer TR, Pope JE, Panchal SJ, Patel VB: **Intrathecal therapy for cancer and non-cancer pain**. *Pain Physician* 2011, **14**(3):219-248.

266. Hayek SM, Helm S, Benyamin RM, Singh V, Bryce DA, Smith HS: **Effectiveness of spinal endoscopic adhesiolysis in post lumbar surgery syndrome: a systematic review**. *Pain Physician* 2009, **12**(2):419-435.

267. Hayes J, Dowling JJ, Peliowski A, Crawford MW, Johnston B: **Patient-Controlled Analgesia Plus Background Opioid Infusion for Postoperative Pain in Children: A Systematic Review and Meta-Analysis of Randomized Trials**. *Anesth Analg* 2016, **123**(4):991-1003.

268. Heesen M, Bohmer J, Klohr S, Hofmann T, Rossaint R, Straube S: **The effect of adding a background infusion to patient-controlled epidural labor analgesia on labor, maternal, and neonatal outcomes: a systematic review and meta-analysis**. *Anesth Analg* 2015, **121**(1):149-158.

269. Heesen M, Klimek M, Hoeks SE, Rossaint R: **Prevention of Spinal Anesthesia-Induced Hypotension During Cesarean Delivery by 5-Hydroxytryptamine-3 Receptor Antagonists: A Systematic Review and Meta-analysis and Meta-regression**. *Anesth Analg* 2016, **123**(4):977-988.

270. Heesen M, Klimek M, Imberger G, Hoeks SE, Rossaint R, Straube S: **Co-administration of dexamethasone with peripheral nerve block: intravenous vs perineural application: systematic review, meta-analysis, meta-regression and trial-sequential analysis**. *Br J Anaesth* 2018, **120**(2):212-227.

271. Heesen M, Klimek M, Rossaint R, Imberger G, Straube S: **Paravertebral block and persistent postoperative pain after breast surgery: meta-analysis and trial sequential analysis**. *Anaesthesia* 2016, **71**(12):1471-1481.

272. Heesen M, Kolhr S, Rossaint R, Straube S: **Prophylactic phenylephrine for caesarean section under spinal anaesthesia: systematic review and meta-analysis**. *Anaesthesia* 2014, **69**(2):143-165.

273. Heesen M, Van de Velde M, Klohr S, Lehberger J, Rossaint R, Straube S: **Meta-analysis of the success of block following combined spinal-epidural vs epidural analgesia during labour**. *Anaesthesia* 2014, **69**(1):64-71.

274. Heesen M, Weibel S, Klimek M, Rossaint R, Arends LR, Kranke P: **Effects of epidural volume extension by saline injection on the efficacy and safety of intrathecal local anaesthetics: systematic review with meta-analysis, meta-regression and trial sequential analysis**. *Anaesthesia* 2017, **72**(11):1398-1411.

275. Helm Ii S, Benyamin RM, Chopra P, Deer TR, Justiz R: **Percutaneous adhesiolysis in the management of chronic low back pain in post lumbar surgery syndrome and spinal stenosis: a systematic review**. *Pain Physician* 2012, **15**(4):E435-462.

276. Helm Ii S, Deer TR, Manchikanti L, Datta S, Chopra P, Singh V, Hirsch JA: **Effectiveness of thermal annular procedures in treating discogenic low back pain**. *Pain Physician* 2012, **15**(3):E279-304.

277. Helm S, Hayek SM, Benyamin RM, Manchikanti L: **Systematic review of the effectiveness of thermal annular procedures in treating discogenic low back pain**. *Pain Physician* 2009, **12**(1):207-232.

278. Helm S, Hayek SM, Colson J, Chopra P, Deer TR, Justiz R, Hameed M, Falco FJ: **Spinal endoscopic adhesiolysis in post lumbar surgery syndrome: an update of assessment of the evidence**. *Pain Physician* 2013, **16**(2 Suppl):SE125-150.

279. Heng Sia AT, Tan KH, Sng BL, Lim Y, Chan ES, Siddiqui FJ: **Hyperbaric versus plain bupivacaine for spinal anesthesia for cesarean delivery**. *Anesth Analg* 2015, **120**(1):132-140.

280. Herrera FJ, Wong J, Chung F: **A systematic review of postoperative recovery outcomes measurements after ambulatory surgery**. *Anesth Analg* 2007, **105**(1):63-69.

281. Higgins C, Smith BH, Matthews K: **Incidence of iatrogenic opioid dependence or abuse in patients with pain who were exposed to opioid analgesic therapy: a systematic review and meta-analysis**. *Br J Anaesth* 2018, **120**(6):1335-1344.

282. Higgins KS, Birnie KA, Chambers CT, Wilson AC, Caes L, Clark AJ, Lynch M, Stinson J, Campbell-Yeo M: **Offspring of parents with chronic pain: a systematic review and meta-analysis of pain, health, psychological, and family outcomes**. *Pain* 2015, **156**(11):2256-2266.

283. Hillyard SG, Bate TE, Corcoran TB, Paech MJ, O'Sullivan G: **Extending epidural analgesia for emergency Caesarean section: a meta-analysis**. *Br J Anaesth* 2011, **107**(5):668-678.

284. Himmelseher S, Durieux ME: **Revising a dogma: ketamine for patients with neurological injury?** *Anesth Analg* 2005, **101**(2):524-534, table of contents.

285. Hindler K, Shaw AD, Samuels J, Fulton S, Collard CD, Riedel B: **Improved postoperative outcomes associated with preoperative statin therapy**. *Anesthesiology* 2006, **105**(6):1260-1272; quiz 1289-1290.

286. Hirose K, Hirose M, Tanaka K, Kawahito S, Tamaki T, Oshita S: **Perioperative management of severe anorexia nervosa**. *Br J Anaesth* 2014, **112**(2):246-254.

287. Hirsch JA, Singh V, Falco FJ, Benyamin RM, Manchikanti L: **Automated percutaneous lumbar discectomy for the contained herniated lumbar disc: a systematic assessment of evidence**. *Pain Physician* 2009, **12**(3):601-620.

288. Ho KM, Tan JA: **Use of L'Abbe and pooled calibration plots to assess the relationship between severity of illness and effectiveness in studies of corticosteroids for severe sepsis**. *Br J Anaesth* 2011, **106**(4):528-536.

289. Ho KY, Gan TJ, Habib AS: **Gabapentin and postoperative pain--a systematic review of randomized controlled trials**. *Pain* 2006, **126**(1-3):91-101.

290. Hojer Karlsen AP, Geisler A, Petersen PL, Mathiesen O, Dahl JB: **Postoperative pain treatment after total hip arthroplasty: a systematic review**. *Pain* 2015, **156**(1):8-30.

291. Hollmann C, Fernandes NL, Biccard BM: **A Systematic Review of Outcomes Associated With Withholding or Continuing Angiotensin-Converting Enzyme Inhibitors and Angiotensin Receptor Blockers Before Noncardiac Surgery**. *Anesth Analg* 2018, **127**(3):678-687.

292. Hooijmans CR, Geessink FJ, Ritskes-Hoitinga M, Scheffer GJ: **A systematic review and meta-analysis of the ability of analgesic drugs to reduce metastasis in experimental cancer models**. *Pain* 2015, **156**(10):1835-1844.

293. Horsten S, Reinke L, Absalom AR, Tulleken JE: **Systematic review of the effects of intensive-care-unit noise on sleep of healthy subjects and the critically ill**. *Br J Anaesth* 2018, **120**(3):443-452.

294. Hoshijima H, Kuratani N, Hirabayashi Y, Takeuchi R, Shiga T, Masaki E: **Pentax Airway Scope(R) vs Macintosh laryngoscope for tracheal intubation in adult patients: a systematic review and meta-analysis**. *Anaesthesia* 2014, **69**(8):911-918.

295. Hounsome J, Greenhalgh J, Schofield-Robinson OJ, Lewis SR, Cook TM, Smith AF: **Nitrous oxide-based vs. nitrous oxide-free general anaesthesia and accidental awareness in surgical patients: an abridged Cochrane systematic review**. *Anaesthesia* 2018, **73**(3):365-374.

296. Hounsome J, Lee A, Greenhalgh J, Lewis SR, Schofield-Robinson OJ, Coldwell CH, Smith AF: **A systematic review of information format and timing before scheduled adult surgery for peri-operative anxiety**. *Anaesthesia* 2017, **72**(10):1265-1272.

297. Hovaguimian F, Lysakowski C, Elia N, Tramer MR: **Effect of intraoperative high inspired oxygen fraction on surgical site infection, postoperative nausea and vomiting, and pulmonary function: systematic review and meta-analysis of randomized controlled trials**. *Anesthesiology* 2013, **119**(2):303-316.

298. Hovaguimian F, Myles PS: **Restrictive versus Liberal Transfusion Strategy in the Perioperative and Acute Care Settings: A Context-specific Systematic Review and Meta-analysis of Randomized Controlled Trials**. *Anesthesiology* 2016, **125**(1):46-61.

299. Hristovska AM, Duch P, Allingstrup M, Afshari A: **The comparative efficacy and safety of sugammadex and neostigmine in reversing neuromuscular blockade in adults. A Cochrane systematic review with meta-analysis and trial sequential analysis**. *Anaesthesia* 2018, **73**(5):631-641.

300. Hu S, Singh M, Wong J, Auckley D, Hershner S, Kakkar R, Thorpy MJ, Chung F: **Anesthetic Management of Narcolepsy Patients During Surgery: A Systematic Review**. *Anesth Analg* 2018, **126**(1):233-246.

301. Hubscher M, Moloney N, Leaver A, Rebbeck T, McAuley JH, Refshauge KM: **Relationship between quantitative sensory testing and pain or disability in people with spinal pain-a systematic review and meta-analysis**. *Pain* 2013, **154**(9):1497-1504.

302. Huguet A, Tougas ME, Hayden J, McGrath PJ, Stinson JN, Chambers CT: **Systematic review with meta-analysis of childhood and adolescent risk and prognostic factors for musculoskeletal pain**. *Pain* 2016, **157**(12):2640-2656.

303. Hurley RW, Cohen SP, Williams KA, Rowlingson AJ, Wu CL: **The analgesic effects of perioperative gabapentin on postoperative pain: a meta-analysis**. *Reg Anesth Pain Med* 2006, **31**(3):237-247.

304. Hurley RW, Lesley MR, Adams MC, Brummett CM, Wu CL: **Pregabalin as a treatment for painful diabetic peripheral neuropathy: a meta-analysis**. *Reg Anesth Pain Med* 2008, **33**(5):389-394.

305. Hussain N, Ferreri TG, Prusick PJ, Banfield L, Long B, Prusick VR, Bhandari M: **Adductor Canal Block Versus Femoral Canal Block for Total Knee Arthroplasty: A Meta-Analysis: What Does the Evidence Suggest?** *Reg Anesth Pain Med* 2016, **41**(3):314-320.

306. Hussain N, Goldar G, Ragina N, Banfield L, Laffey JG, Abdallah FW: **Suprascapular and Interscalene Nerve Block for Shoulder Surgery: A Systematic Review and Meta-analysis**. *Anesthesiology* 2017, **127**(6):998-1013.

307. Hussain N, Grzywacz VP, Ferreri CA, Atrey A, Banfield L, Shaparin N, Vydyanathan A: **Investigating the Efficacy of Dexmedetomidine as an Adjuvant to Local Anesthesia in Brachial Plexus Block: A Systematic Review and Meta-Analysis of 18 Randomized Controlled Trials**. *Reg Anesth Pain Med* 2017, **42**(2):184-196.

308. Hussain N, Shastri U, McCartney CJL, Gilron I, Fillingim RB, Clarke H, Katz J, Juni P, Laupacis A, Wijeysundera D *et al*: **Should thoracic paravertebral blocks be used to prevent chronic postsurgical pain after breast cancer surgery? A systematic analysis of evidence in light of IMMPACT recommendations**. *Pain* 2018, **159**(10):1955-1971.

309. Huynh TM, Marret E, Bonnet F: **Combination of dexamethasone and local anaesthetic solution in peripheral nerve blocks: A meta-analysis of randomised controlled trials**. *Eur J Anaesthesiol* 2015, **32**(11):751-758.

310. Hwang IC, Park JY, Myung SK, Ahn HY, Fukuda K, Liao Q: **OPRM1 A118G gene variant and postoperative opioid requirement: a systematic review and meta-analysis**. *Anesthesiology* 2014, **121**(4):825-834.

311. Ilhan E, Chee E, Hush J, Moloney N: **The prevalence of neuropathic pain is high after treatment for breast cancer: a systematic review**. *Pain* 2017, **158**(11):2082-2091.

312. Imberger G, Orr A, Thorlund K, Wetterslev J, Myles P, Moller AM: **Does anaesthesia with nitrous oxide affect mortality or cardiovascular morbidity? A systematic review with meta-analysis and trial sequential analysis**. *Br J Anaesth* 2014, **112**(3):410-426.

313. Ip HY, Abrishami A, Peng PW, Wong J, Chung F: **Predictors of postoperative pain and analgesic consumption: a qualitative systematic review**. *Anesthesiology* 2009, **111**(3):657-677.

314. Jackson T, Thomas S, Stabile V, Shotwell M, Han X, McQueen K: **A Systematic Review and Meta-Analysis of the Global Burden of Chronic Pain Without Clear Etiology in Low- and Middle-Income Countries: Trends in Heterogeneous Data and a Proposal for New Assessment Methods**. *Anesth Analg* 2016, **123**(3):739-748.

315. Jafari H, Courtois I, Van den Bergh O, Vlaeyen JWS, Van Diest I: **Pain and respiration: a systematic review**. *Pain* 2017, **158**(6):995-1006.

316. Jain G, Mahendra V, Singhal S, Dzara K, Pilla TR, Manworren R, Kaye AD: **Long-term neuropsychological effects of opioid use in children: a descriptive literature review**. *Pain Physician* 2014, **17**(2):109-118.

317. Jasper JF, Hayek SM: **Implanted occipital nerve stimulators**. *Pain Physician* 2008, **11**(2):187-200.

318. Jelting Y, Weibel S, Afshari A, Pace NL, Jokinen J, Artmann T, Eberhart LHJ, Kranke P: **Patient-controlled analgesia with remifentanil vs. alternative parenteral methods for pain management in labour: a Cochrane systematic review**. *Anaesthesia* 2017, **72**(8):1016-1028.

319. Jerath A, Panckhurst J, Parotto M, Lightfoot N, Wasowicz M, Ferguson ND, Steel A, Beattie WS: **Safety and Efficacy of Volatile Anesthetic Agents Compared With Standard Intravenous Midazolam/Propofol Sedation in Ventilated Critical Care Patients: A Meta-analysis and Systematic Review of Prospective Trials**. *Anesth Analg* 2017, **124**(4):1190-1199.

320. Johansson T, Fritsch G, Flamm M, Hansbauer B, Bachofner N, Mann E, Bock M, Sonnichsen AC: **Effectiveness of non-cardiac preoperative testing in non-cardiac elective surgery: a systematic review**. *Br J Anaesth* 2013, **110**(6):926-939.

321. Johnson M, Martinson M: **Efficacy of electrical nerve stimulation for chronic musculoskeletal pain: a meta-analysis of randomized controlled trials**. *Pain* 2007, **130**(1-2):157-165.

322. Johnson RL, Cannon EK, Mantilla CB, Cook DA: **Cricoid pressure training using simulation: a systematic review and meta-analysis**. *Br J Anaesth* 2013, **111**(3):338-346.

323. Johnson RL, Kopp SL, Burkle CM, Duncan CM, Jacob AK, Erwin PJ, Murad MH, Mantilla CB: **Neuraxial vs general anaesthesia for total hip and total knee arthroplasty: a systematic review of comparative-effectiveness research**. *Br J Anaesth* 2016, **116**(2):163-176.

324. Johnson RL, Kopp SL, Hebl JR, Erwin PJ, Mantilla CB: **Falls and major orthopaedic surgery with peripheral nerve blockade: a systematic review and meta-analysis**. *Br J Anaesth* 2013, **110**(4):518-528.

325. Johnston J, Pal S, Nagele P: **Perioperative torsade de pointes: a systematic review of published case reports**. *Anesth Analg* 2013, **117**(3):559-564.

326. Jones CPL, Fawker-Corbett J, Groom P, Morton B, Lister C, Mercer SJ: **Human factors in preventing complications in anaesthesia: a systematic review**. *Anaesthesia* 2018, **73 Suppl 1**:12-24.

327. Joshi GP, Ahmad S, Riad W, Eckert S, Chung F: **Selection of obese patients undergoing ambulatory surgery: a systematic review of the literature**. *Anesth Analg* 2013, **117**(5):1082-1091.

328. Joshi GP, Ankichetty SP, Gan TJ, Chung F: **Society for Ambulatory Anesthesia consensus statement on preoperative selection of adult patients with obstructive sleep apnea scheduled for ambulatory surgery**. *Anesth Analg* 2012, **115**(5):1060-1068.

329. Joshi GP, Bonnet F, Shah R, Wilkinson RC, Camu F, Fischer B, Neugebauer EA, Rawal N, Schug SA, Simanski C *et al*: **A systematic review of randomized trials evaluating regional techniques for postthoracotomy analgesia**. *Anesth Analg* 2008, **107**(3):1026-1040.

330. Joshi GP, Chung F, Vann MA, Ahmad S, Gan TJ, Goulson DT, Merrill DG, Twersky R, Society for Ambulatory A: **Society for Ambulatory Anesthesia consensus statement on perioperative blood glucose management in diabetic patients undergoing ambulatory surgery**. *Anesth Analg* 2010, **111**(6):1378-1387.

331. Julien-Marsollier F, Michelet D, Bellon M, Horlin AL, Devys JM, Dahmani S: **Muscle relaxation for tracheal intubation during paediatric anaesthesia: A meta-analysis and trial sequential analysis**. *Eur J Anaesthesiol* 2017, **34**(8):550-561.

332. Kalra R, Arora G, Patel N, Doshi R, Berra L, Arora P, Bajaj NS: **Targeted Temperature Management After Cardiac Arrest: Systematic Review and Meta-analyses**. *Anesth Analg* 2018, **126**(3):867-875.

333. Kamper SJ, Rebbeck TJ, Maher CG, McAuley JH, Sterling M: **Course and prognostic factors of whiplash: a systematic review and meta-analysis**. *Pain* 2008, **138**(3):617-629.

334. Karam O, Gebistorf F, Wetterslev J, Afshari A: **The effect of inhaled nitric oxide in acute respiratory distress syndrome in children and adults: a Cochrane Systematic Review with trial sequential analysis**. *Anaesthesia* 2017, **72**(1):106-117.

335. Katz NP, Paillard FC, Edwards RR: **Review of the performance of quantitative sensory testing methods to detect hyperalgesia in chronic pain patients on long-term opioids**. *Anesthesiology* 2015, **122**(3):677-685.

336. Kaw R, Chung F, Pasupuleti V, Mehta J, Gay PC, Hernandez AV: **Meta-analysis of the association between obstructive sleep apnoea and postoperative outcome**. *Br J Anaesth* 2012, **109**(6):897-906.

337. Kawakami H, Mihara T, Nakamura N, Ka K, Goto T: **Effect of an Intravenous Dexamethasone Added to Caudal Local Anesthetics to Improve Postoperative Pain: A Systematic Review and Meta-analysis With Trial Sequential Analysis**. *Anesth Analg* 2017, **125**(6):2072-2080.

338. Kawano-Dourado L, Zampieri FG, Azevedo LCP, Correa TD, Figueiro M, Semler MW, Kellum JA, Cavalcanti AB: **Low- Versus High-Chloride Content Intravenous Solutions for Critically Ill and Perioperative Adult Patients: A Systematic Review and Meta-analysis**. *Anesth Analg* 2018, **126**(2):513-521.

339. Kaya S, Hermans L, Willems T, Roussel N, Meeus M: **Central sensitization in urogynecological chronic pelvic pain: a systematic literature review**. *Pain Physician* 2013, **16**(4):291-308.

340. Kekecs Z, Nagy T, Varga K: **The effectiveness of suggestive techniques in reducing postoperative side effects: a meta-analysis of randomized controlled trials**. *Anesth Analg* 2014, **119**(6):1407-1419.

341. Kelly FE, Nolan JP: **The effects of mild induced hypothermia on the myocardium: a systematic review**. *Anaesthesia* 2010, **65**(5):505-515.

342. Kessler J, Marhofer P, Hopkins PM, Hollmann MW: **Peripheral regional anaesthesia and outcome: lessons learned from the last 10 years**. *Br J Anaesth* 2015, **114**(5):728-745.

343. Khan JS, Margarido C, Devereaux PJ, Clarke H, McLellan A, Choi S: **Preoperative celecoxib in noncardiac surgery: A systematic review and meta-analysis of randomised controlled trials**. *Eur J Anaesthesiol* 2016, **33**(3):204-214.

344. Kim HJ, Yang GS, Greenspan JD, Downton KD, Griffith KA, Renn CL, Johantgen M, Dorsey SG: **Racial and ethnic differences in experimental pain sensitivity: systematic review and meta-analysis**. *Pain* 2017, **158**(2):194-211.

345. Kim SH, Lilot M, Murphy LS, Sidhu KS, Yu Z, Rinehart J, Cannesson M: **Accuracy of continuous noninvasive hemoglobin monitoring: a systematic review and meta-analysis**. *Anesth Analg* 2014, **119**(2):332-346.

346. Kim SH, Lilot M, Sidhu KS, Rinehart J, Yu Z, Canales C, Cannesson M: **Accuracy and precision of continuous noninvasive arterial pressure monitoring compared with invasive arterial pressure: a systematic review and meta-analysis**. *Anesthesiology* 2014, **120**(5):1080-1097.

347. Kim WH, Hur M, Park SK, Jung DE, Kang P, Yoo S, Bahk JH: **Pharmacological interventions for protecting renal function after cardiac surgery: a Bayesian network meta-analysis of comparative effectiveness**. *Anaesthesia* 2018, **73**(8):1019-1031.

348. King MR, Ladha KS, Gelineau AM, Anderson TA: **Perioperative Dextromethorphan as an Adjunct for Postoperative Pain: A Meta-analysis of Randomized Controlled Trials**. *Anesthesiology* 2016, **124**(3):696-705.

349. King S, Chambers CT, Huguet A, MacNevin RC, McGrath PJ, Parker L, MacDonald AJ: **The epidemiology of chronic pain in children and adolescents revisited: a systematic review**. *Pain* 2011, **152**(12):2729-2738.

350. Kirkham KR, Grape S, Martin R, Albrecht E: **Analgesic efficacy of local infiltration analgesia vs. femoral nerve block after anterior cruciate ligament reconstruction: a systematic review and meta-analysis**. *Anaesthesia* 2017, **72**(12):1542-1553.

351. Kirkham KR, Jacot-Guillarmod A, Albrecht E: **Optimal Dose of Perineural Dexamethasone to Prolong Analgesia After Brachial Plexus Blockade: A Systematic Review and Meta-analysis**. *Anesth Analg* 2018, **126**(1):270-279.

352. Klimek M, Rossaint R, van de Velde M, Heesen M: **Combined spinal-epidural vs. spinal anaesthesia for caesarean section: meta-analysis and trial-sequential analysis**. *Anaesthesia* 2018, **73**(7):875-888.

353. Knezevic NN, Anantamongkol U, Candido KD: **Perineural dexamethasone added to local anesthesia for brachial plexus block improves pain but delays block onset and motor blockade recovery**. *Pain Physician* 2015, **18**(1):1-14.

354. Koers L, Janjatovic D, Stevens MF, Preckel B: **The emergency paediatric surgical airway: A systematic review**. *Eur J Anaesthesiol* 2018, **35**(8):558-565.

355. Komatsu R, Turan AM, Orhan-Sungur M, McGuire J, Radke OC, Apfel CC: **Remifentanil for general anaesthesia: a systematic review**. *Anaesthesia* 2007, **62**(12):1266-1280.

356. Kossowsky J, Donado C, Berde CB: **Immediate rescue designs in pediatric analgesic trials: a systematic review and meta-analysis**. *Anesthesiology* 2015, **122**(1):150-171.

357. Kotze A, Scally A, Howell S: **Efficacy and safety of different techniques of paravertebral block for analgesia after thoracotomy: a systematic review and metaregression**. *Br J Anaesth* 2009, **103**(5):626-636.

358. Koyyalagunta D, Bruera E, Solanki DR, Nouri KH, Burton AW, Toro MP, Bruel BM, Manchikanti L: **A systematic review of randomized trials on the effectiveness of opioids for cancer pain**. *Pain Physician* 2012, **15**(3 Suppl):ES39-58.

359. Kreienbuhl L, Elia N, Pfeil-Beun E, Walder B, Tramer MR: **Patient-Controlled Versus Clinician-Controlled Sedation With Propofol: Systematic Review and Meta-analysis With Trial Sequential Analyses**. *Anesth Analg* 2018, **127**(4):873-880.

360. Kumar B, Kalita J, Kumar G, Misra UK: **Central poststroke pain: a review of pathophysiology and treatment**. *Anesth Analg* 2009, **108**(5):1645-1657.

361. Kumar G, Stendall C, Mistry R, Gurusamy K, Walker D: **A comparison of total intravenous anaesthesia using propofol with sevoflurane or desflurane in ambulatory surgery: systematic review and meta-analysis**. *Anaesthesia* 2014, **69**(10):1138-1150.

362. Kuratani N, Oi Y: **Greater incidence of emergence agitation in children after sevoflurane anesthesia as compared with halothane: a meta-analysis of randomized controlled trials**. *Anesthesiology* 2008, **109**(2):225-232.

363. Kuriyama A, Aga M, Maeda H: **Topical benzydamine hydrochloride for prevention of postoperative sore throat in adults undergoing tracheal intubation for elective surgery: a systematic review and meta-analysis**. *Anaesthesia* 2018, **73**(7):889-900.

364. Kuriyama A, Maeda H, Sun R, Aga M: **Topical application of corticosteroids to tracheal tubes to prevent postoperative sore throat in adults undergoing tracheal intubation: a systematic review and meta-analysis**. *Anaesthesia* 2018, **73**(12):1546-1556.

365. Lam DK, Corry GN, Tsui BC: **Evidence for the Use of Ultrasound Imaging in Pediatric Regional Anesthesia: A Systematic Review**. *Reg Anesth Pain Med* 2016, **41**(2):229-241.

366. Lam EWK, Chung F, Wong J: **Sleep-Disordered Breathing, Postoperative Delirium, and Cognitive Impairment**. *Anesth Analg* 2017, **124**(5):1626-1635.

367. Lam T, Nagappa M, Wong J, Singh M, Wong D, Chung F: **Continuous Pulse Oximetry and Capnography Monitoring for Postoperative Respiratory Depression and Adverse Events: A Systematic Review and Meta-analysis**. *Anesth Analg* 2017, **125**(6):2019-2029.

368. Landoni G, Greco T, Biondi-Zoccai G, Nigro Neto C, Febres D, Pintaudi M, Pasin L, Cabrini L, Finco G, Zangrillo A: **Anaesthetic drugs and survival: a Bayesian network meta-analysis of randomized trials in cardiac surgery**. *Br J Anaesth* 2013, **111**(6):886-896.

369. Landoni G, Isella F, Greco M, Zangrillo A, Royse CF: **Benefits and risks of epidural analgesia in cardiac surgery**. *Br J Anaesth* 2015, **115**(1):25-32.

370. Law LS, Lo EA, Gan TJ: **Xenon Anesthesia: A Systematic Review and Meta-Analysis of Randomized Controlled Trials**. *Anesth Analg* 2016, **122**(3):678-697.

371. Law LS, Tan M, Bai Y, Miller TE, Li YJ, Gan TJ: **Paravertebral Block for Inguinal Herniorrhaphy: A Systematic Review and Meta-Analysis of Randomized Controlled Trials**. *Anesth Analg* 2015, **121**(2):556-569.

372. Lawrence R, Mogford D, Colvin L: **Systematic review to determine which validated measurement tools can be used to assess risk of problematic analgesic use in patients with chronic pain**. *Br J Anaesth* 2017, **119**(6):1092-1109.

373. Lee A, Mu JL, Joynt GM, Chiu CH, Lai VKW, Gin T, Underwood MJ: **Risk prediction models for delirium in the intensive care unit after cardiac surgery: a systematic review and independent external validation**. *Br J Anaesth* 2017, **118**(3):391-399.

374. Lee H, Hubscher M, Moseley GL, Kamper SJ, Traeger AC, Mansell G, McAuley JH: **How does pain lead to disability? A systematic review and meta-analysis of mediation studies in people with back and neck pain**. *Pain* 2015, **156**(6):988-997.

375. Leffert LR, Dubois HM, Butwick AJ, Carvalho B, Houle TT, Landau R: **Neuraxial Anesthesia in Obstetric Patients Receiving Thromboprophylaxis With Unfractionated or Low-Molecular-Weight Heparin: A Systematic Review of Spinal Epidural Hematoma**. *Anesth Analg* 2017, **125**(1):223-231.

376. Leighton BL, Wall MH, Lockhart EM, Phillips LE, Zatta AJ: **Use of recombinant factor VIIa in patients with amniotic fluid embolism: a systematic review of case reports**. *Anesthesiology* 2011, **115**(6):1201-1208.

377. Leite VF, Buehler AM, El Abd O, Benyamin RM, Pimentel DC, Chen J, Hsing WT, Mazloomdoost D, Amadera JE: **Anti-nerve growth factor in the treatment of low back pain and radiculopathy: a systematic review and a meta-analysis**. *Pain Physician* 2014, **17**(1):E45-60.

378. Lemoine A, Mazoit JX, Bonnet F: **Modelling of the optimal bupivacaine dose for spinal anaesthesia in ambulatory surgery based on data from systematic review**. *Eur J Anaesthesiol* 2016, **33**(11):846-852.

379. Leong WL, Sng BL, Sia AT: **A comparison between remifentanil and meperidine for labor analgesia: a systematic review**. *Anesth Analg* 2011, **113**(4):818-825.

380. Levy JH, Grottke O, Fries D, Kozek-Langenecker S: **Therapeutic Plasma Transfusion in Bleeding Patients: A Systematic Review**. *Anesth Analg* 2017, **124**(4):1268-1276.

381. Lewis SR, Butler AR, Parker J, Cook TM, Schofield-Robinson OJ, Smith AF: **Videolaryngoscopy versus direct laryngoscopy for adult patients requiring tracheal intubation: a Cochrane Systematic Review**. *Br J Anaesth* 2017, **119**(3):369-383.

382. Likis FE, Andrews JC, Collins MR, Lewis RM, Seroogy JJ, Starr SA, Walden RR, McPheeters ML: **Nitrous oxide for the management of labor pain: a systematic review**. *Anesth Analg* 2014, **118**(1):153-167.

383. Lin CW, McAuley JH, Macedo L, Barnett DC, Smeets RJ, Verbunt JA: **Relationship between physical activity and disability in low back pain: a systematic review and meta-analysis**. *Pain* 2011, **152**(3):607-613.

384. Lin J, Zhang L, Yang HL: **Unilateral versus bilateral balloon kyphoplasty for osteoporotic vertebral compression fractures**. *Pain Physician* 2013, **16**(5):447-453.

385. Lin YC, Wan L, Jamison RN: **Using Integrative Medicine in Pain Management: An Evaluation of Current Evidence**. *Anesth Analg* 2017, **125**(6):2081-2093.

386. Linassi F, Zanatta P, Tellaroli P, Ori C, Carron M: **Isolated forearm technique: a meta-analysis of connected consciousness during different general anaesthesia regimens**. *Br J Anaesth* 2018, **121**(1):198-209.

387. Liossi C, Failo A, Schoth DE, Williams G, Howard RF: **The effectiveness of online pain resources for health professionals: a systematic review with subset meta-analysis of educational intervention studies**. *Pain* 2018, **159**(4):631-643.

388. Liu J, Li X, Tang D, Cui X, Li X, Yao M, Yu P, Qian X, Wang Y, Jiang H: **Comparing pain reduction following vertebroplasty and conservative treatment for osteoporotic vertebral compression fractures: a meta-analysis of randomized controlled trials**. *Pain Physician* 2013, **16**(5):455-464.

389. Liu J, Rossaint R, Sanders RD, Coburn M: **Toxic and protective effects of inhaled anaesthetics on the developing animal brain: systematic review and update of recent experimental work**. *Eur J Anaesthesiol* 2014, **31**(12):669-677.

390. Liu SS: **Evidence Basis for Ultrasound-Guided Block Characteristics Onset, Quality, and Duration**. *Reg Anesth Pain Med* 2016, **41**(2):205-220.

391. Liu SS, Ngeow JE, Yadeau JT: **Ultrasound-guided regional anesthesia and analgesia: a qualitative systematic review**. *Reg Anesth Pain Med* 2009, **34**(1):47-59.

392. Liu SS, Strodtbeck WM, Richman JM, Wu CL: **A comparison of regional versus general anesthesia for ambulatory anesthesia: a meta-analysis of randomized controlled trials**. *Anesth Analg* 2005, **101**(6):1634-1642.

393. Liu SS, Togioka BM, Hurley RW, Vu CM, Hanna MN, Murphy JD, Wu CL: **Methodological quality of randomized controlled trials of postoperative epidural analgesia: validation of the Epidural Analgesia Trial Checklist as a specific instrument to evaluate methodology**. *Reg Anesth Pain Med* 2010, **35**(6):549-555.

394. Liu SS, Wu CL: **The effect of analgesic technique on postoperative patient-reported outcomes including analgesia: a systematic review**. *Anesth Analg* 2007, **105**(3):789-808.

395. Liu SS, Wu CL: **Effect of postoperative analgesia on major postoperative complications: a systematic update of the evidence**. *Anesth Analg* 2007, **104**(3):689-702.

396. Liu TT, Li L, Wan L, Zhang CH, Yao WL: **Videolaryngoscopy vs. Macintosh laryngoscopy for double-lumen tube intubation in thoracic surgery: a systematic review and meta-analysis**. *Anaesthesia* 2018, **73**(8):997-1007.

397. Liu ZQ, Chen XB, Li HB, Qiu MT, Duan T: **A comparison of remifentanil parturient-controlled intravenous analgesia with epidural analgesia: a meta-analysis of randomized controlled trials**. *Anesth Analg* 2014, **118**(3):598-603.

398. Lorello GR, Cook DA, Johnson RL, Brydges R: **Simulation-based training in anaesthesiology: a systematic review and meta-analysis**. *Br J Anaesth* 2014, **112**(2):231-245.

399. Lu Y, Jiang H, Zhu YS: **Airtraq laryngoscope versus conventional Macintosh laryngoscope: a systematic review and meta-analysis**. *Anaesthesia* 2011, **66**(12):1160-1167.

400. Lundstrom LH, Duez CHV, Norskov AK, Rosenstock CV, Thomsen JL, Moller AM, Strande S, Wetterslev J: **Effects of avoidance or use of neuromuscular blocking agents on outcomes in tracheal intubation: a Cochrane systematic review**. *Br J Anaesth* 2018, **120**(6):1381-1393.

401. Lusted A, Roerecke M, Goldner E, Rehm J, Fischer B: **Prevalence of pain among nonmedical prescription opioid users in substance use treatment populations: systematic review and meta-analyses**. *Pain Physician* 2013, **16**(6):E671-684.

402. Lynch ME, Campbell F, Clark AJ, Dunbar MJ, Goldstein D, Peng P, Stinson J, Tupper H: **A systematic review of the effect of waiting for treatment for chronic pain**. *Pain* 2008, **136**(1-2):97-116.

403. Lysakowski C, Dumont L, Czarnetzki C, Tramer MR: **Magnesium as an adjuvant to postoperative analgesia: a systematic review of randomized trials**. *Anesth Analg* 2007, **104**(6):1532-1539, table of contents.

404. Macfarlane AJ, Prasad GA, Chan VW, Brull R: **Does regional anaesthesia improve outcome after total hip arthroplasty? A systematic review**. *Br J Anaesth* 2009, **103**(3):335-345.

405. MacKenzie KK, Britt-Spells AM, Sands LP, Leung JM: **Processed Electroencephalogram Monitoring and Postoperative Delirium: A Systematic Review and Meta-analysis**. *Anesthesiology* 2018, **129**(3):417-427.

406. MacPherson H, Vertosick EA, Foster NE, Lewith G, Linde K, Sherman KJ, Witt CM, Vickers AJ: **The persistence of the effects of acupuncture after a course of treatment: a meta-analysis of patients with chronic pain**. *Pain* 2017, **158**(5):784-793.

407. Magalhaes FN, Dotta L, Sasse A, Teixera MJ, Fonoff ET: **Ozone therapy as a treatment for low back pain secondary to herniated disc: a systematic review and meta-analysis of randomized controlled trials**. *Pain Physician* 2012, **15**(2):E115-129.

408. Maitra S, Bhattacharjee S, Khanna P, Baidya DK: **High-frequency ventilation does not provide mortality benefit in comparison with conventional lung-protective ventilation in acute respiratory distress syndrome: a meta-analysis of the randomized controlled trials**. *Anesthesiology* 2015, **122**(4):841-851.

409. Maitra S, Khanna P, Baidya DK: **Comparison of laryngeal mask airway Supreme and laryngeal mask airway Pro-Seal for controlled ventilation during general anaesthesia in adult patients: systematic review with meta-analysis**. *Eur J Anaesthesiol* 2014, **31**(5):266-273.

410. Manchikanti KN, Atluri S, Singh V, Geffert S, Sehgal N, Falco FJ: **An update of evaluation of therapeutic thoracic facet joint interventions**. *Pain Physician* 2012, **15**(4):E463-481.

411. Manchikanti L, Ailinani H, Koyyalagunta D, Datta S, Singh V, Eriator I, Sehgal N, Shah R, Benyamin R, Vallejo R *et al*: **A systematic review of randomized trials of long-term opioid management for chronic non-cancer pain**. *Pain Physician* 2011, **14**(2):91-121.

412. Manchikanti L, Benyamin RM, Singh V, Falco FJ, Hameed H, Derby R, Wolfer LR, Helm S, 2nd, Calodney AK, Datta S *et al*: **An update of the systematic appraisal of the accuracy and utility of lumbar discography in chronic low back pain**. *Pain Physician* 2013, **16**(2 Suppl):SE55-95.

413. Manchikanti L, Buenaventura RM, Manchikanti KN, Ruan X, Gupta S, Smith HS, Christo PJ, Ward SP: **Effectiveness of therapeutic lumbar transforaminal epidural steroid injections in managing lumbar spinal pain**. *Pain Physician* 2012, **15**(3):E199-245.

414. Manchikanti L, Derby R, Benyamin RM, Helm S, Hirsch JA: **A systematic review of mechanical lumbar disc decompression with nucleoplasty**. *Pain Physician* 2009, **12**(3):561-572.

415. Manchikanti L, Falco FJ, Benyamin RM, Caraway DL, Deer TR, Singh V, Hameed H, Hirsch JA: **An update of the systematic assessment of mechanical lumbar disc decompression with nucleoplasty**. *Pain Physician* 2013, **16**(2 Suppl):SE25-54.

416. Manchikanti L, Falco FJ, Benyamin RM, Caraway DL, Helm Ii S, Wargo BW, Hansen H, Parr AT, Singh V, Hirsch JA: **Assessment of infection control practices for interventional techniques: a best evidence synthesis of safe injection practices and use of single-dose medication vials**. *Pain Physician* 2012, **15**(5):E573-614.

417. Manchikanti L, Falco FJ, Benyamin RM, Caraway DL, Kaye AD, Helm S, 2nd, Wargo BW, Hansen H, Parr AT, Singh V *et al*: **Assessment of bleeding risk of interventional techniques: a best evidence synthesis of practice patterns and perioperative management of anticoagulant and antithrombotic therapy**. *Pain Physician* 2013, **16**(2 Suppl):SE261-318.

418. Manchikanti L, Nampiaparampil DE, Candido KD, Bakshi S, Grider JS, Falco FJ, Sehgal N, Hirsch JA: **Do cervical epidural injections provide long-term relief in neck and upper extremity pain? A systematic review**. *Pain Physician* 2015, **18**(1):39-60.

419. Manchikanti L, Singh V, Calodney AK, Helm S, 2nd, Deer TR, Benyamin RM, Falco FJ, Hirsch JA: **Percutaneous lumbar mechanical disc decompression utilizing Dekompressor(R): an update of current evidence**. *Pain Physician* 2013, **16**(2 Suppl):SE1-24.

420. Manchikanti L, Singh V, Falco FJ, Calodney AK, Onyewu O, Helm S, 2nd, Benyamin RM, Hirsch JA: **An updated review of automated percutaneous mechanical lumbar discectomy for the contained herniated lumbar disc**. *Pain Physician* 2013, **16**(2 Suppl):SE151-184.

421. Mar GJ, Barrington MJ, McGuirk BR: **Acute compartment syndrome of the lower limb and the effect of postoperative analgesia on diagnosis**. *Br J Anaesth* 2009, **102**(1):3-11.

422. Marcuzzi A, Dean CM, Wrigley PJ, Hush JM: **Early changes in somatosensory function in spinal pain: a systematic review and meta-analysis**. *Pain* 2015, **156**(2):203-214.

423. Martin C, Jacob M, Vicaut E, Guidet B, Van Aken H, Kurz A: **Effect of waxy maize-derived hydroxyethyl starch 130/0.4 on renal function in surgical patients**. *Anesthesiology* 2013, **118**(2):387-394.

424. Martinez V, Beloeil H, Marret E, Fletcher D, Ravaud P, Trinquart L: **Non-opioid analgesics in adults after major surgery: systematic review with network meta-analysis of randomized trials**. *Br J Anaesth* 2017, **118**(1):22-31.

425. Martinez V, Guichard L, Fletcher D: **Effect of combining tramadol and morphine in adult surgical patients: a systematic review and meta-analysis of randomized trials**. *Br J Anaesth* 2015, **114**(3):384-395.

426. Martinez V, Pichard X, Fletcher D: **Perioperative pregabalin administration does not prevent chronic postoperative pain: systematic review with a meta-analysis of randomized trials**. *Pain* 2017, **158**(5):775-783.

427. Marucci M, Cinnella G, Perchiazzi G, Brienza N, Fiore T: **Patient-requested neuraxial analgesia for labor: impact on rates of cesarean and instrumental vaginal delivery**. *Anesthesiology* 2007, **106**(5):1035-1045.

428. Mauermann WJ, Shilling AM, Zuo Z: **A comparison of neuraxial block versus general anesthesia for elective total hip replacement: a meta-analysis**. *Anesth Analg* 2006, **103**(4):1018-1025.

429. Maund E, McDaid C, Rice S, Wright K, Jenkins B, Woolacott N: **Paracetamol and selective and non-selective non-steroidal anti-inflammatory drugs for the reduction in morphine-related side-effects after major surgery: a systematic review**. *Br J Anaesth* 2011, **106**(3):292-297.

430. Mayhew D, Sahgal N, Khirwadkar R, Hunter JM, Banerjee A: **Analgesic efficacy of bilateral superficial cervical plexus block for thyroid surgery: meta-analysis and systematic review**. *Br J Anaesth* 2018, **120**(2):241-251.

431. Mazzinari G, Ball L, Serpa Neto A, Errando CL, Dondorp AM, Bos LD, Gama de Abreu M, Pelosi P, Schultz MJ: **The fragility of statistically significant findings in randomised controlled anaesthesiology trials: systematic review of the medical literature**. *Br J Anaesth* 2018, **120**(5):935-941.

432. McCartney CJ, Duggan E, Apatu E: **Should we add clonidine to local anesthetic for peripheral nerve blockade? A qualitative systematic review of the literature**. *Reg Anesth Pain Med* 2007, **32**(4):330-338.

433. McIlroy DR, Myles PS, Phillips LE, Smith JA: **Antifibrinolytics in cardiac surgical patients receiving aspirin: a systematic review and meta-analysis**. *Br J Anaesth* 2009, **102**(2):168-178.

434. McNicol ED, Tzortzopoulou A, Cepeda MS, Francia MB, Farhat T, Schumann R: **Single-dose intravenous paracetamol or propacetamol for prevention or treatment of postoperative pain: a systematic review and meta-analysis**. *Br J Anaesth* 2011, **106**(6):764-775.

435. McQuay HJ, Poon KH, Derry S, Moore RA: **Acute pain: combination treatments and how we measure their efficacy**. *Br J Anaesth* 2008, **101**(1):69-76.

436. Mekhail N, Visnjevac O, Azer G, Mehanny DS, Agrawal P, Foorsov V: **Spinal Cord Stimulation 50 Years Later: Clinical Outcomes of Spinal Cord Stimulation Based on Randomized Clinical Trials-A Systematic Review**. *Reg Anesth Pain Med* 2018, **43**(4):391-406.

437. Meng H, Johnston B, Englesakis M, Moulin DE, Bhatia A: **Selective Cannabinoids for Chronic Neuropathic Pain: A Systematic Review and Meta-analysis**. *Anesth Analg* 2017, **125**(5):1638-1652.

438. Meng T, Zhong Z, Meng L: **Impact of spinal anaesthesia vs. general anaesthesia on peri-operative outcome in lumbar spine surgery: a systematic review and meta-analysis of randomised, controlled trials**. *Anaesthesia* 2017, **72**(3):391-401.

439. Mercer SJ, Jones CP, Bridge M, Clitheroe E, Morton B, Groom P: **Systematic review of the anaesthetic management of non-iatrogenic acute adult airway trauma**. *Br J Anaesth* 2016, **117 Suppl 1**:i49-i59.

440. Messina A, Longhini F, Coppo C, Pagni A, Lungu R, Ronco C, Cattaneo MA, Dore S, Sotgiu G, Navalesi P: **Use of the Fluid Challenge in Critically Ill Adult Patients: A Systematic Review**. *Anesth Analg* 2017, **125**(5):1532-1543.

441. Meylan N, Elia N, Lysakowski C, Tramer MR: **Benefit and risk of intrathecal morphine without local anaesthetic in patients undergoing major surgery: meta-analysis of randomized trials**. *Br J Anaesth* 2009, **102**(2):156-167.

442. Mhyre JM, Greenfield ML, Tsen LC, Polley LS: **A systematic review of randomized controlled trials that evaluate strategies to avoid epidural vein cannulation during obstetric epidural catheter placement**. *Anesth Analg* 2009, **108**(4):1232-1242.

443. Michelet D, Andreu-Gallien J, Bensalah T, Hilly J, Wood C, Nivoche Y, Mantz J, Dahmani S: **A meta-analysis of the use of nonsteroidal antiinflammatory drugs for pediatric postoperative pain**. *Anesth Analg* 2012, **114**(2):393-406.

444. Mihai R, Blair E, Kay H, Cook TM: **A quantitative review and meta-analysis of performance of non-standard laryngoscopes and rigid fibreoptic intubation aids**. *Anaesthesia* 2008, **63**(7):745-760.

445. Mihara T, Asakura A, Owada G, Yokoi A, Ka K, Goto T: **A network meta-analysis of the clinical properties of various types of supraglottic airway device in children**. *Anaesthesia* 2017, **72**(10):1251-1264.

446. Mihara T, Nakamura N, Ka K, Oba MS, Goto T: **Effects of melatonin premedication to prevent emergence agitation after general anaesthesia in children: A systematic review and meta-analysis with trial sequential analysis**. *Eur J Anaesthesiol* 2015, **32**(12):862-871.

447. Mihara T, Tojo K, Uchimoto K, Morita S, Goto T: **Reevaluation of the effectiveness of ramosetron for preventing postoperative nausea and vomiting: a systematic review and meta-analysis**. *Anesth Analg* 2013, **117**(2):329-339.

448. Mihara T, Uchimoto K, Morita S, Goto T: **The efficacy of lidocaine to prevent laryngospasm in children: a systematic review and meta-analysis**. *Anaesthesia* 2014, **69**(12):1388-1396.

449. Mishriky BM, Habib AS: **Metoclopramide for nausea and vomiting prophylaxis during and after Caesarean delivery: a systematic review and meta-analysis**. *Br J Anaesth* 2012, **108**(3):374-383.

450. Mishriky BM, Habib AS: **Nicotine for postoperative analgesia: a systematic review and meta-analysis**. *Anesth Analg* 2014, **119**(2):268-275.

451. Mishriky BM, Waldron NH, Habib AS: **Impact of pregabalin on acute and persistent postoperative pain: a systematic review and meta-analysis**. *Br J Anaesth* 2015, **114**(1):10-31.

452. Moana-Filho EJ, Herrero Babiloni A, Theis-Mahon NR: **Endogenous pain modulation in chronic orofacial pain: a systematic review and meta-analysis**. *Pain* 2018, **159**(8):1441-1455.

453. Moller TP, Madsen MD, Fuhrmann L, Ostergaard D: **Postoperative handover: characteristics and considerations on improvement: a systematic review**. *Eur J Anaesthesiol* 2013, **30**(5):229-242.

454. Moore RA, Derry S, Wiffen PJ, Banerjee S, Karan R, Glimm E, Wiksten A, Aldington D, Eccleston C: **Estimating relative efficacy in acute postoperative pain: network meta-analysis is consistent with indirect comparison to placebo alone**. *Pain* 2018, **159**(11):2234-2244.

455. Moore RA, Derry S, Wiffen PJ, Straube S, Bendtsen L: **Evidence for efficacy of acute treatment of episodic tension-type headache: methodological critique of randomised trials for oral treatments**. *Pain* 2014, **155**(11):2220-2228.

456. Moran J, Wilson F, Guinan E, McCormick P, Hussey J, Moriarty J: **Role of cardiopulmonary exercise testing as a risk-assessment method in patients undergoing intra-abdominal surgery: a systematic review**. *Br J Anaesth* 2016, **116**(2):177-191.

457. Morasco BJ, Gritzner S, Lewis L, Oldham R, Turk DC, Dobscha SK: **Systematic review of prevalence, correlates, and treatment outcomes for chronic non-cancer pain in patients with comorbid substance use disorder**. *Pain* 2011, **152**(3):488-497.

458. Morin AM, Kranke P, Wulf H, Stienstra R, Eberhart LH: **The effect of stimulating versus nonstimulating catheter techniques for continuous regional anesthesia: a semiquantitative systematic review**. *Reg Anesth Pain Med* 2010, **35**(2):194-199.

459. Morley S, Williams A, Eccleston C: **Examining the evidence about psychological treatments for chronic pain: time for a paradigm shift?** *Pain* 2013, **154**(10):1929-1931.

460. Morrison AP, Hunter JM, Halpern SH, Banerjee A: **Effect of intrathecal magnesium in the presence or absence of local anaesthetic with and without lipophilic opioids: a systematic review and meta-analysis**. *Br J Anaesth* 2013, **110**(5):702-712.

461. Moyce Z, Rodseth RN, Biccard BM: **The efficacy of peri-operative interventions to decrease postoperative delirium in non-cardiac surgery: a systematic review and meta-analysis**. *Anaesthesia* 2014, **69**(3):259-269.

462. Mulvey MR, Boland EG, Bouhassira D, Freynhagen R, Hardy J, Hjermstad MJ, Mercadante S, Perez C, Bennett MI: **Neuropathic pain in cancer: systematic review, performance of screening tools and analysis of symptom profiles**. *Br J Anaesth* 2017, **119**(4):765-774.

463. Munirama S, McLeod G: **A systematic review and meta-analysis of ultrasound versus electrical stimulation for peripheral nerve location and blockade**. *Anaesthesia* 2015, **70**(9):1084-1091.

464. Myles PS, Boney O, Botti M, Cyna AM, Gan TJ, Jensen MP, Kehlet H, Kurz A, De Oliveira GS, Jr., Peyton P *et al*: **Systematic review and consensus definitions for the Standardised Endpoints in Perioperative Medicine (StEP) initiative: patient comfort**. *Br J Anaesth* 2018, **120**(4):705-711.

465. Nagappa M, Ho G, Patra J, Wong J, Singh M, Kaw R, Cheng D, Chung F: **Postoperative Outcomes in Obstructive Sleep Apnea Patients Undergoing Cardiac Surgery: A Systematic Review and Meta-analysis of Comparative Studies**. *Anesth Analg* 2017, **125**(6):2030-2037.

466. Nagappa M, Mokhlesi B, Wong J, Wong DT, Kaw R, Chung F: **The Effects of Continuous Positive Airway Pressure on Postoperative Outcomes in Obstructive Sleep Apnea Patients Undergoing Surgery: A Systematic Review and Meta-analysis**. *Anesth Analg* 2015, **120**(5):1013-1023.

467. Nagappa M, Patra J, Wong J, Subramani Y, Singh M, Ho G, Wong DT, Chung F: **Association of STOP-Bang Questionnaire as a Screening Tool for Sleep Apnea and Postoperative Complications: A Systematic Review and Bayesian Meta-analysis of Prospective and Retrospective Cohort Studies**. *Anesth Analg* 2017, **125**(4):1301-1308.

468. Nagar VR, Birthi P, Grider JS, Asopa A: **Systematic review of radiofrequency ablation and pulsed radiofrequency for management of cervicogenic headache**. *Pain Physician* 2015, **18**(2):109-130.

469. Naguib M, Kopman AF, Ensor JE: **Neuromuscular monitoring and postoperative residual curarisation: a meta-analysis**. *Br J Anaesth* 2007, **98**(3):302-316.

470. Nair GS, Abrishami A, Lermitte J, Chung F: **Systematic review of spinal anaesthesia using bupivacaine for ambulatory knee arthroscopy**. *Br J Anaesth* 2009, **102**(3):307-315.

471. Narouze S, Souzdalnitski D: **Obesity and chronic pain: systematic review of prevalence and implications for pain practice**. *Reg Anesth Pain Med* 2015, **40**(2):91-111.

472. Neal JM: **Ultrasound-guided regional anesthesia and patient safety: An evidence-based analysis**. *Reg Anesth Pain Med* 2010, **35**(2 Suppl):S59-67.

473. Neal JM: **Ultrasound-Guided Regional Anesthesia and Patient Safety: Update of an Evidence-Based Analysis**. *Reg Anesth Pain Med* 2016, **41**(2):195-204.

474. Nestoriuc Y, Martin A: **Efficacy of biofeedback for migraine: a meta-analysis**. *Pain* 2007, **128**(1-2):111-127.

475. Newman S, Stygall J, Hirani S, Shaefi S, Maze M: **Postoperative cognitive dysfunction after noncardiac surgery: a systematic review**. *Anesthesiology* 2007, **106**(3):572-590.

476. Ng K, Grounds R, Haga K, Carter G, Clarke S, Loveless R, Glyde D, McClymont K, Alston RP: **The efficacy and safety of tight blood glucose control during heart surgery: a systematic review and meta-analysis**. *Anaesthesia* 2009, **64**(12):1389.

477. Ng KT, Yap JLL: **Continuous infusion vs. intermittent bolus injection of furosemide in acute decompensated heart failure: systematic review and meta-analysis of randomised controlled trials**. *Anaesthesia* 2018, **73**(2):238-247.

478. Ng SC, Habib AS, Sodha S, Carvalho B, Sultan P: **High-dose versus low-dose local anaesthetic for transversus abdominis plane block post-Caesarean delivery analgesia: a meta-analysis**. *Br J Anaesth* 2018, **120**(2):252-263.

479. Niel-Weise BS, Stijnen T, van den Broek PJ: **Should in-line filters be used in peripheral intravenous catheters to prevent infusion-related phlebitis? A systematic review of randomized controlled trials**. *Anesth Analg* 2010, **110**(6):1624-1629.

480. Niesters M, Dahan A, Kest B, Zacny J, Stijnen T, Aarts L, Sarton E: **Do sex differences exist in opioid analgesia? A systematic review and meta-analysis of human experimental and clinical studies**. *Pain* 2010, **151**(1):61-68.

481. Niesters M, Overdyk F, Smith T, Aarts L, Dahan A: **Opioid-induced respiratory depression in paediatrics: a review of case reports**. *Br J Anaesth* 2013, **110**(2):175-182.

482. O'Connor AB, Schwid SR, Herrmann DN, Markman JD, Dworkin RH: **Pain associated with multiple sclerosis: systematic review and proposed classification**. *Pain* 2008, **137**(1):96-111.

483. O'Doherty AF, West M, Jack S, Grocott MP: **Preoperative aerobic exercise training in elective intra-cavity surgery: a systematic review**. *Br J Anaesth* 2013, **110**(5):679-689.

484. O'Donnell CM, McLoughlin L, Patterson CC, Clarke M, McCourt KC, McBrien ME, McAuley DF, Shields MO: **Perioperative outcomes in the context of mode of anaesthesia for patients undergoing hip fracture surgery: systematic review and meta-analysis**. *Br J Anaesth* 2018, **120**(1):37-50.

485. Oliver CM, Walker E, Giannaris S, Grocott MP, Moonesinghe SR: **Risk assessment tools validated for patients undergoing emergency laparotomy: a systematic review**. *Br J Anaesth* 2015, **115**(6):849-860.

486. Ong CK, Seymour RA, Lirk P, Merry AF: **Combining paracetamol (acetaminophen) with nonsteroidal antiinflammatory drugs: a qualitative systematic review of analgesic efficacy for acute postoperative pain**. *Anesth Analg* 2010, **110**(4):1170-1179.

487. Opperer M, Cozowicz C, Bugada D, Mokhlesi B, Kaw R, Auckley D, Chung F, Memtsoudis SG: **Does Obstructive Sleep Apnea Influence Perioperative Outcome? A Qualitative Systematic Review for the Society of Anesthesia and Sleep Medicine Task Force on Preoperative Preparation of Patients with Sleep-Disordered Breathing**. *Anesth Analg* 2016, **122**(5):1321-1334.

488. Orbegozo Cortes D, Gamarano Barros T, Njimi H, Vincent JL: **Crystalloids versus colloids: exploring differences in fluid requirements by systematic review and meta-regression**. *Anesth Analg* 2015, **120**(2):389-402.

489. Orhan-Sungur M, Kranke P, Sessler D, Apfel CC: **Does supplemental oxygen reduce postoperative nausea and vomiting? A meta-analysis of randomized controlled trials**. *Anesth Analg* 2008, **106**(6):1733-1738.

490. Page EA, Taylor KL: **Paravertebral block in paediatric abdominal surgery-a systematic review and meta-analysis of randomized trials**. *Br J Anaesth* 2017, **118**(2):159-166.

491. Palermo TM, Eccleston C, Lewandowski AS, Williams AC, Morley S: **Randomized controlled trials of psychological therapies for management of chronic pain in children and adolescents: an updated meta-analytic review**. *Pain* 2010, **148**(3):387-397.

492. Pandit JJ, Satya-Krishna R, Gration P: **Superficial or deep cervical plexus block for carotid endarterectomy: a systematic review of complications**. *Br J Anaesth* 2007, **99**(2):159-169.

493. Park SK, Lee SY, Kim WH, Park HS, Lim YJ, Bahk JH: **Comparison of Supraclavicular and Infraclavicular Brachial Plexus Block: A Systemic Review of Randomized Controlled Trials**. *Anesth Analg* 2017, **124**(2):636-644.

494. Parr AT, Diwan S, Abdi S: **Lumbar interlaminar epidural injections in managing chronic low back and lower extremity pain: a systematic review**. *Pain Physician* 2009, **12**(1):163-188.

495. Parr AT, Manchikanti L, Hameed H, Conn A, Manchikanti KN, Benyamin RM, Diwan S, Singh V, Abdi S: **Caudal epidural injections in the management of chronic low back pain: a systematic appraisal of the literature**. *Pain Physician* 2012, **15**(3):E159-198.

496. Partridge JS, Harari D, Martin FC, Dhesi JK: **The impact of pre-operative comprehensive geriatric assessment on postoperative outcomes in older patients undergoing scheduled surgery: a systematic review**. *Anaesthesia* 2014, **69 Suppl 1**:8-16.

497. Pasin L, Nardelli P, Pintaudi M, Greco M, Zambon M, Cabrini L, Zangrillo A: **Closed-Loop Delivery Systems Versus Manually Controlled Administration of Total IV Anesthesia: A Meta-analysis of Randomized Clinical Trials**. *Anesth Analg* 2017, **124**(2):456-464.

498. Passos Mdo C, Duro D, Fregni F: **CNS or classic drugs for the treatment of pain in functional dyspepsia? A systematic review and meta-analysis of the literature**. *Pain Physician* 2008, **11**(5):597-609.

499. Patel SD, Habib AS, Phillips S, Carvalho B, Sultan P: **The Effect of Glycopyrrolate on the Incidence of Hypotension and Vasopressor Requirement During Spinal Anesthesia for Cesarean Delivery: A Meta-analysis**. *Anesth Analg* 2018, **126**(2):552-558.

500. Patel VB, Manchikanti L, Singh V, Schultz DM, Hayek SM, Smith HS: **Systematic review of intrathecal infusion systems for long-term management of chronic non-cancer pain**. *Pain Physician* 2009, **12**(2):345-360.

501. Paton F, Paulden M, Chambers D, Heirs M, Duffy S, Hunter JM, Sculpher M, Woolacott N: **Sugammadex compared with neostigmine/glycopyrrolate for routine reversal of neuromuscular block: a systematic review and economic evaluation**. *Br J Anaesth* 2010, **105**(5):558-567.

502. Paul JE, Arya A, Hurlburt L, Cheng J, Thabane L, Tidy A, Murthy Y: **Femoral nerve block improves analgesia outcomes after total knee arthroplasty: a meta-analysis of randomized controlled trials**. *Anesthesiology* 2010, **113**(5):1144-1162.

503. Peerdeman KJ, van Laarhoven AI, Keij SM, Vase L, Rovers MM, Peters ML, Evers AW: **Relieving patients' pain with expectation interventions: a meta-analysis**. *Pain* 2016, **157**(6):1179-1191.

504. Peng K, Liu HY, Wu SR, Liu H, Zhang ZC, Ji FH: **Does Propofol Anesthesia Lead to Less Postoperative Pain Compared With Inhalational Anesthesia?: A Systematic Review and Meta-analysis**. *Anesth Analg* 2016, **123**(4):846-858.

505. Perez-Gonzalez O, Cuellar-Guzman LF, Navarrete-Pacheco M, Ortiz-Martinez JJ, Williams WH, Cata JP: **Impact of Regional Anesthesia on Gastroesophageal Cancer Surgery Outcomes: A Systematic Review of the Literature**. *Anesth Analg* 2018, **127**(3):753-758.

506. Perez-Gonzalez O, Cuellar-Guzman LF, Soliz J, Cata JP: **Impact of Regional Anesthesia on Recurrence, Metastasis, and Immune Response in Breast Cancer Surgery: A Systematic Review of the Literature**. *Reg Anesth Pain Med* 2017, **42**(6):751-756.

507. Perlas A, Chaparro LE, Chin KJ: **Lumbar Neuraxial Ultrasound for Spinal and Epidural Anesthesia: A Systematic Review and Meta-Analysis**. *Reg Anesth Pain Med* 2016, **41**(2):251-260.

508. Petersen GL, Finnerup NB, Colloca L, Amanzio M, Price DD, Jensen TS, Vase L: **The magnitude of nocebo effects in pain: a meta-analysis**. *Pain* 2014, **155**(8):1426-1434.

509. Peyton PJ, Chong SW: **Minimally invasive measurement of cardiac output during surgery and critical care: a meta-analysis of accuracy and precision**. *Anesthesiology* 2010, **113**(5):1220-1235.

510. Phoenix SI, Paravastu S, Columb M, Vincent JL, Nirmalan M: **Does a higher positive end expiratory pressure decrease mortality in acute respiratory distress syndrome? A systematic review and meta-analysis**. *Anesthesiology* 2009, **110**(5):1098-1105.

511. Pickard A, Davies P, Birnie K, Beringer R: **Systematic review and meta-analysis of the effect of intraoperative alpha(2)-adrenergic agonists on postoperative behaviour in children**. *Br J Anaesth* 2014, **112**(6):982-990.

512. Pieters BMA, Maas EHA, Knape JTA, van Zundert AAJ: **Videolaryngoscopy vs. direct laryngoscopy use by experienced anaesthetists in patients with known difficult airways: a systematic review and meta-analysis**. *Anaesthesia* 2017, **72**(12):1532-1541.

513. Pike A, Hearn L, Williams AC: **Effectiveness of psychological interventions for chronic pain on health care use and work absence: systematic review and meta-analysis**. *Pain* 2016, **157**(4):777-785.

514. Pikwer A, Akeson J, Lindgren S: **Complications associated with peripheral or central routes for central venous cannulation**. *Anaesthesia* 2012, **67**(1):65-71.

515. Pimentel DC, El Abd O, Benyamin RM, Buehler AM, Leite VF, Mazloomdoost D, Chen J, Hsing WT, Amadera JE: **Anti-tumor necrosis factor antagonists in the treatment of low back pain and radiculopathy: a systematic review and meta-analysis**. *Pain Physician* 2014, **17**(1):E27-44.

516. Pincus T, Holt N, Vogel S, Underwood M, Savage R, Walsh DA, Taylor SJ: **Cognitive and affective reassurance and patient outcomes in primary care: a systematic review**. *Pain* 2013, **154**(11):2407-2416.

517. Popping DM, Elia N, Marret E, Wenk M, Tramer MR: **Clonidine as an adjuvant to local anesthetics for peripheral nerve and plexus blocks: a meta-analysis of randomized trials**. *Anesthesiology* 2009, **111**(2):406-415.

518. Popping DM, Elia N, Marret E, Wenk M, Tramer MR: **Opioids added to local anesthetics for single-shot intrathecal anesthesia in patients undergoing minor surgery: a meta-analysis of randomized trials**. *Pain* 2012, **153**(4):784-793.

519. Popping DM, Elia N, Wenk M, Tramer MR: **Combination of a reduced dose of an intrathecal local anesthetic with a small dose of an opioid: a meta-analysis of randomized trials**. *Pain* 2013, **154**(8):1383-1390.

520. Poppler LH, Parikh RP, Bichanich MJ, Rebehn K, Bettlach CR, Mackinnon SE, Moore AM: **Surgical interventions for the treatment of painful neuroma: a comparative meta-analysis**. *Pain* 2018, **159**(2):214-223.

521. Potter LJ, Doleman B, Moppett IK: **A systematic review of pre-operative anaemia and blood transfusion in patients with fractured hips**. *Anaesthesia* 2015, **70**(4):483-500.

522. Potvin S, Marchand S: **Hypoalgesia in schizophrenia is independent of antipsychotic drugs: a systematic quantitative review of experimental studies**. *Pain* 2008, **138**(1):70-78.

523. Prin M, Guglielminotti J, Moitra V, Li G: **Prophylactic Ondansetron for the Prevention of Intrathecal Fentanyl- or Sufentanil-Mediated Pruritus: A Meta-Analysis of Randomized Trials**. *Anesth Analg* 2016, **122**(2):402-409.

524. Qiu Q, Choi SW, Wong SS, Irwin MG, Cheung CW: **Effects of intra-operative maintenance of general anaesthesia with propofol on postoperative pain outcomes - a systematic review and meta-analysis**. *Anaesthesia* 2016, **71**(10):1222-1233.

525. Raiman M, Mitchell CG, Biccard BM, Rodseth RN: **Comparison of hydroxyethyl starch colloids with crystalloids for surgical patients: A systematic review and meta-analysis**. *Eur J Anaesthesiol* 2016, **33**(1):42-48.

526. Rajagopalan S, Mascha E, Na J, Sessler DI: **The effects of mild perioperative hypothermia on blood loss and transfusion requirement**. *Anesthesiology* 2008, **108**(1):71-77.

527. Redfern G, Rodseth RN, Biccard BM: **Outcomes in vascular surgical patients with isolated postoperative troponin leak: a meta-analysis**. *Anaesthesia* 2011, **66**(7):604-610.

528. Ren ZY, Xu XQ, Bao YP, He J, Shi L, Deng JH, Gao XJ, Tang HL, Wang YM, Lu L: **The impact of genetic variation on sensitivity to opioid analgesics in patients with postoperative pain: a systematic review and meta-analysis**. *Pain Physician* 2015, **18**(2):131-152.

529. Richman JM, Liu SS, Courpas G, Wong R, Rowlingson AJ, McGready J, Cohen SR, Wu CL: **Does continuous peripheral nerve block provide superior pain control to opioids? A meta-analysis**. *Anesth Analg* 2006, **102**(1):248-257.

530. Rodrigo C, Samarakoon L, Fernando SD, Rajapakse S: **A meta-analysis of magnesium for tetanus**. *Anaesthesia* 2012, **67**(12):1370-1374.

531. Rodseth RN, Padayachee L, Biccard BM: **A meta-analysis of the utility of pre-operative brain natriuretic peptide in predicting early and intermediate-term mortality and major adverse cardiac events in vascular surgical patients**. *Anaesthesia* 2008, **63**(11):1226-1233.

532. Rong LQ, Kamel MK, Rahouma M, White RS, Lichtman AD, Pryor KO, Girardi LN, Gaudino M: **Cerebrospinal-fluid drain-related complications in patients undergoing open and endovascular repairs of thoracic and thoraco-abdominal aortic pathologies: a systematic review and meta-analysis**. *Br J Anaesth* 2018, **120**(5):904-913.

533. Ross A, Young J, Hedin R, Aran G, Demand A, Stafford A, Worley J, Moore M, Vassar M: **A systematic review of outcomes in postoperative pain studies in paediatric and adolescent patients: towards development of a core outcome set**. *Anaesthesia* 2018, **73**(3):375-383.

534. Roughead T, Sewell D, Ryerson CJ, Fisher JH, Flexman AM: **Internet-Based Resources Frequently Provide Inaccurate and Out-of-Date Recommendations on Preoperative Fasting: A Systematic Review**. *Anesth Analg* 2016, **123**(6):1463-1468.

535. Rudroju N, Bansal D, Talakokkula ST, Gudala K, Hota D, Bhansali A, Ghai B: **Comparative efficacy and safety of six antidepressants and anticonvulsants in painful diabetic neuropathy: a network meta-analysis**. *Pain Physician* 2013, **16**(6):E705-714.

536. Ruppen W, Derry S, McQuay H, Moore RA: **Incidence of epidural hematoma, infection, and neurologic injury in obstetric patients with epidural analgesia/anesthesia**. *Anesthesiology* 2006, **105**(2):394-399.

537. Salinas FV: **Ultrasound and review of evidence for lower extremity peripheral nerve blocks**. *Reg Anesth Pain Med* 2010, **35**(2 Suppl):S16-25.

538. Sanfilippo F, Corredor C, Arcadipane A, Landesberg G, Vieillard-Baron A, Cecconi M, Fletcher N: **Tissue Doppler assessment of diastolic function and relationship with mortality in critically ill septic patients: a systematic review and meta-analysis**. *Br J Anaesth* 2017, **119**(4):583-594.

539. Schaefer MS, Kranke P, Weibel S, Kreysing R, Kienbaum P: **Total intravenous anaesthesia versus single-drug pharmacological antiemetic prophylaxis in adults: A systematic review and meta-analysis**. *Eur J Anaesthesiol* 2016, **33**(10):750-760.

540. Schaub I, Lysakowski C, Elia N, Tramer MR: **Low-dose droperidol (</=1 mg or </=15 mug kg-1) for the prevention of postoperative nausea and vomiting in adults: quantitative systematic review of randomised controlled trials**. *Eur J Anaesthesiol* 2012, **29**(6):286-294.

541. Schier R, Guerra D, Aguilar J, Pratt GF, Hernandez M, Boddu K, Riedel B: **Epidural space identification: a meta-analysis of complications after air versus liquid as the medium for loss of resistance**. *Anesth Analg* 2009, **109**(6):2012-2021.

542. Schmutz J, Manser T: **Do team processes really have an effect on clinical performance? A systematic literature review**. *Br J Anaesth* 2013, **110**(4):529-544.

543. Schnabel A, Eberhart LH, Muellenbach R, Morin AM, Roewer N, Kranke P: **Efficacy of perphenazine to prevent postoperative nausea and vomiting: a quantitative systematic review**. *Eur J Anaesthesiol* 2010, **27**(12):1044-1051.

544. Schnabel A, Hahn N, Broscheit J, Muellenbach RM, Rieger L, Roewer N, Kranke P: **Remifentanil for labour analgesia: a meta-analysis of randomised controlled trials**. *Eur J Anaesthesiol* 2012, **29**(4):177-185.

545. Schnabel A, Meyer-Friessem CH, Reichl SU, Zahn PK, Pogatzki-Zahn EM: **Is intraoperative dexmedetomidine a new option for postoperative pain treatment? A meta-analysis of randomized controlled trials**. *Pain* 2013, **154**(7):1140-1149.

546. Schnabel A, Meyer-Friessem CH, Zahn PK, Pogatzki-Zahn EM: **Ultrasound compared with nerve stimulation guidance for peripheral nerve catheter placement: a meta-analysis of randomized controlled trials**. *Br J Anaesth* 2013, **111**(4):564-572.

547. Schnabel A, Poepping DM, Kranke P, Zahn PK, Pogatzki-Zahn EM: **Efficacy and adverse effects of ketamine as an additive for paediatric caudal anaesthesia: a quantitative systematic review of randomized controlled trials**. *Br J Anaesth* 2011, **107**(4):601-611.

548. Schnabel A, Reichl SU, Kranke P, Pogatzki-Zahn EM, Zahn PK: **Efficacy and safety of paravertebral blocks in breast surgery: a meta-analysis of randomized controlled trials**. *Br J Anaesth* 2010, **105**(6):842-852.

549. Schnabel A, Reichl SU, Zahn PK, Pogatzki-Zahn EM, Meyer-Friessem CH: **Efficacy and safety of buprenorphine in peripheral nerve blocks: A meta-analysis of randomised controlled trials**. *Eur J Anaesthesiol* 2017, **34**(9):576-586.

550. Schouten LR, Schultz MJ, van Kaam AH, Juffermans NP, Bos AP, Wosten-van Asperen RM: **Association between Maturation and Aging and Pulmonary Responses in Animal Models of Lung Injury: A Systematic Review**. *Anesthesiology* 2015, **123**(2):389-408.

551. Schreiber JU, Lysakowski C, Fuchs-Buder T, Tramer MR: **Prevention of succinylcholine-induced fasciculation and myalgia: a meta-analysis of randomized trials**. *Anesthesiology* 2005, **103**(4):877-884.

552. Schwenk ES, Grant AE, Torjman MC, McNulty SE, Baratta JL, Viscusi ER: **The Efficacy of Peripheral Opioid Antagonists in Opioid-Induced Constipation and Postoperative Ileus: A Systematic Review of the Literature**. *Reg Anesth Pain Med* 2017, **42**(6):767-777.

553. Seangleulur A, Vanasbodeekul P, Prapaitrakool S, Worathongchai S, Anothaisintawee T, McEvoy M, Vendittoli PA, Attia J, Thakkinstian A: **The efficacy of local infiltration analgesia in the early postoperative period after total knee arthroplasty: A systematic review and meta-analysis**. *Eur J Anaesthesiol* 2016, **33**(11):816-831.

554. Sehmbi H, Brull R, Shah UJ, El-Boghdadly K, Nguyen D, Joshi GP, Abdallah FW: **Evidence Basis for Regional Anesthesia in Ambulatory Arthroscopic Knee Surgery and Anterior Cruciate Ligament Reconstruction: Part II: Adductor Canal Nerve Block-A Systematic Review and Meta-analysis**. *Anesth Analg* 2019, **128**(2):223-238.

555. Seretny M, Currie GL, Sena ES, Ramnarine S, Grant R, MacLeod MR, Colvin LA, Fallon M: **Incidence, prevalence, and predictors of chemotherapy-induced peripheral neuropathy: A systematic review and meta-analysis**. *Pain* 2014, **155**(12):2461-2470.

556. Serraino GF, Murphy GJ: **Routine use of viscoelastic blood tests for diagnosis and treatment of coagulopathic bleeding in cardiac surgery: updated systematic review and meta-analysis**. *Br J Anaesth* 2017, **118**(6):823-833.

557. Shanthanna H, Mendis N, Goel A: **Cervical epidural analgesia in current anaesthesia practice: systematic review of its clinical utility and rationale, and technical considerations**. *Br J Anaesth* 2016, **116**(2):192-207.

558. Sharma S, Balireddy RK, Vorenkamp KE, Durieux ME: **Beyond opioid patient-controlled analgesia: a systematic review of analgesia after major spine surgery**. *Reg Anesth Pain Med* 2012, **37**(1):79-98.

559. Shiga T, Wajima Z, Inoue T, Sakamoto A: **Aprotinin in major orthopedic surgery: a systematic review of randomized controlled trials**. *Anesth Analg* 2005, **101**(6):1602-1607.

560. Si X, Xu H, Liu Z, Wu J, Cao D, Chen J, Chen M, Liu Y, Guan X: **Does Respiratory Variation in Inferior Vena Cava Diameter Predict Fluid Responsiveness in Mechanically Ventilated Patients? A Systematic Review and Meta-analysis**. *Anesth Analg* 2018, **127**(5):1157-1164.

561. Siempos, II, Vardakas KZ, Falagas ME: **Closed tracheal suction systems for prevention of ventilator-associated pneumonia**. *Br J Anaesth* 2008, **100**(3):299-306.

562. Singh V, Benyamin RM, Datta S, Falco FJ, Helm S, 2nd, Manchikanti L: **Systematic review of percutaneous lumbar mechanical disc decompression utilizing Dekompressor**. *Pain Physician* 2009, **12**(3):589-599.

563. Singh V, Manchikanti L, Benyamin RM, Helm S, Hirsch JA: **Percutaneous lumbar laser disc decompression: a systematic review of current evidence**. *Pain Physician* 2009, **12**(3):573-588.

564. Singh V, Manchikanti L, Calodney AK, Staats PS, Falco FJ, Caraway DL, Hirsch JA, Cohen SP: **Percutaneous lumbar laser disc decompression: an update of current evidence**. *Pain Physician* 2013, **16**(2 Suppl):SE229-260.

565. Slagt C, Malagon I, Groeneveld AB: **Systematic review of uncalibrated arterial pressure waveform analysis to determine cardiac output and stroke volume variation**. *Br J Anaesth* 2014, **112**(4):626-637.

566. Smith HS, Colson J, Sehgal N: **An update of evaluation of intravenous sedation on diagnostic spinal injection procedures**. *Pain Physician* 2013, **16**(2 Suppl):SE217-228.

567. Smith LM, Cozowicz C, Uda Y, Memtsoudis SG, Barrington MJ: **Neuraxial and Combined Neuraxial/General Anesthesia Compared to General Anesthesia for Major Truncal and Lower Limb Surgery: A Systematic Review and Meta-analysis**. *Anesth Analg* 2017, **125**(6):1931-1945.

568. Smith TB, Stonell C, Purkayastha S, Paraskevas P: **Cardiopulmonary exercise testing as a risk assessment method in non cardio-pulmonary surgery: a systematic review**. *Anaesthesia* 2009, **64**(8):883-893.

569. Sng BL, Han NLR, Leong WL, Sultana R, Siddiqui FJ, Assam PN, Chan ES, Tan KH, Sia AT: **Hyperbaric vs. isobaric bupivacaine for spinal anaesthesia for elective caesarean section: a Cochrane systematic review**. *Anaesthesia* 2018, **73**(4):499-511.

570. Sole-Lleonart C, Rouby JJ, Blot S, Poulakou G, Chastre J, Palmer LB, Bassetti M, Luyt CE, Pereira JM, Riera J *et al*: **Nebulization of Antiinfective Agents in Invasively Mechanically Ventilated Adults: A Systematic Review and Meta-analysis**. *Anesthesiology* 2017, **126**(5):890-908.

571. Som A, Bhattacharjee S, Maitra S, Arora MK, Baidya DK: **Combination of 5-HT3 Antagonist and Dexamethasone Is Superior to 5-HT3 Antagonist Alone for PONV Prophylaxis After Laparoscopic Surgeries: A Meta-analysis**. *Anesth Analg* 2016, **123**(6):1418-1426.

572. Sondekoppam RV, Tsui BC: **Factors Associated With Risk of Neurologic Complications After Peripheral Nerve Blocks: A Systematic Review**. *Anesth Analg* 2017, **124**(2):645-660.

573. Spahn DR: **Anemia and patient blood management in hip and knee surgery: a systematic review of the literature**. *Anesthesiology* 2010, **113**(2):482-495.

574. Sparkes E, Raphael JH, Duarte RV, LeMarchand K, Jackson C, Ashford RL: **A systematic literature review of psychological characteristics as determinants of outcome for spinal cord stimulation therapy**. *Pain* 2010, **150**(2):284-289.

575. Spearing NM, Connelly LB, Gargett S, Sterling M: **Does injury compensation lead to worse health after whiplash? A systematic review**. *Pain* 2012, **153**(6):1274-1282.

576. Spence J, Belley-Cote E, Ma HK, Donald S, Centofanti J, Hussain S, Gupta S, Devereaux PJ, Whitlock R: **Efficacy and safety of inhaled anaesthetic for postoperative sedation during mechanical ventilation in adult cardiac surgery patients: a systematic review and meta-analysis**. *Br J Anaesth* 2017, **118**(5):658-669.

577. Srikandarajah S, Gilron I: **Systematic review of movement-evoked pain versus pain at rest in postsurgical clinical trials and meta-analyses: a fundamental distinction requiring standardized measurement**. *Pain* 2011, **152**(8):1734-1739.

578. Steenberg J, Moller AM: **Systematic review of the effects of fascia iliaca compartment block on hip fracture patients before operation**. *Br J Anaesth* 2018, **120**(6):1368-1380.

579. Stevanovic A, Rossaint R, Fritz HG, Froeba G, Heine J, Puehringer FK, Tonner PH, Coburn M: **Airway reactions and emergence times in general laryngeal mask airway anaesthesia: a meta-analysis**. *Eur J Anaesthesiol* 2015, **32**(2):106-116.

580. Stevens AJ, Woodman RJ, Owen H: **The effect of ondansetron on the efficacy of postoperative tramadol: a systematic review and meta-analysis of a drug interaction**. *Anaesthesia* 2015, **70**(2):209-218.

581. Stinson JN, Kavanagh T, Yamada J, Gill N, Stevens B: **Systematic review of the psychometric properties, interpretability and feasibility of self-report pain intensity measures for use in clinical trials in children and adolescents**. *Pain* 2006, **125**(1-2):143-157.

582. Stockings E, Campbell G, Hall WD, Nielsen S, Zagic D, Rahman R, Murnion B, Farrell M, Weier M, Degenhardt L: **Cannabis and cannabinoids for the treatment of people with chronic noncancer pain conditions: a systematic review and meta-analysis of controlled and observational studies**. *Pain* 2018, **159**(10):1932-1954.

583. Straarup TS, Hausenloy DJ, Rolighed Larsen JK: **Cardiac troponins and volatile anaesthetics in coronary artery bypass graft surgery: A systematic review, meta-analysis and trial sequential analysis**. *Eur J Anaesthesiol* 2016, **33**(6):396-407.

584. Straube S, Harden M, Schroder H, Arendacka B, Fan X, Moore RA, Friede T: **Back schools for the treatment of chronic low back pain: possibility of benefit but no convincing evidence after 47 years of research-systematic review and meta-analysis**. *Pain* 2016, **157**(10):2160-2172.

585. Stubbs B, Thompson T, Acaster S, Vancampfort D, Gaughran F, Correll CU: **Decreased pain sensitivity among people with schizophrenia: a meta-analysis of experimental pain induction studies**. *Pain* 2015, **156**(11):2121-2131.

586. Stundner O, Memtsoudis SG: **Regional anesthesia and analgesia in critically ill patients: a systematic review**. *Reg Anesth Pain Med* 2012, **37**(5):537-544.

587. Su YC, Chen CC, Lee YK, Lee JY, Lin KJ: **Comparison of video laryngoscopes with direct laryngoscopy for tracheal intubation: a meta-analysis of randomised trials**. *Eur J Anaesthesiol* 2011, **28**(11):788-795.

588. Sultan P, Habib AS, Cho Y, Carvalho B: **The Effect of patient warming during Caesarean delivery on maternal and neonatal outcomes: a meta-analysis**. *Br J Anaesth* 2015, **115**(4):500-510.

589. Sultan P, Halpern SH, Pushpanathan E, Patel S, Carvalho B: **The Effect of Intrathecal Morphine Dose on Outcomes After Elective Cesarean Delivery: A Meta-Analysis**. *Anesth Analg* 2016, **123**(1):154-164.

590. Sun Y, Gan TJ: **Acupuncture for the management of chronic headache: a systematic review**. *Anesth Analg* 2008, **107**(6):2038-2047.

591. Sun Y, Gan TJ, Dubose JW, Habib AS: **Acupuncture and related techniques for postoperative pain: a systematic review of randomized controlled trials**. *Br J Anaesth* 2008, **101**(2):151-160.

592. Sun Y, Li T, Gan TJ: **The Effects of Perioperative Regional Anesthesia and Analgesia on Cancer Recurrence and Survival After Oncology Surgery: A Systematic Review and Meta-Analysis**. *Reg Anesth Pain Med* 2015, **40**(5):589-598.

593. Suppan L, Tramer MR, Niquille M, Grosgurin O, Marti C: **Alternative intubation techniques vs Macintosh laryngoscopy in patients with cervical spine immobilization: systematic review and meta-analysis of randomized controlled trials**. *Br J Anaesth* 2016, **116**(1):27-36.

594. Suresh S, Schaldenbrand K, Wallis B, De Oliveira GS, Jr.: **Regional anaesthesia to improve pain outcomes in paediatric surgical patients: a qualitative systematic review of randomized controlled trials**. *Br J Anaesth* 2014, **113**(3):375-390.

595. Svircevic V, van Dijk D, Nierich AP, Passier MP, Kalkman CJ, van der Heijden GJ, Bax L: **Meta-analysis of thoracic epidural anesthesia versus general anesthesia for cardiac surgery**. *Anesthesiology* 2011, **114**(2):271-282.

596. Symons JA, Myles PS: **Myocardial protection with volatile anaesthetic agents during coronary artery bypass surgery: a meta-analysis**. *Br J Anaesth* 2006, **97**(2):127-136.

597. Szakmany T, Russell P, Wilkes AR, Hall JE: **Effect of early tracheostomy on resource utilization and clinical outcomes in critically ill patients: meta-analysis of randomized controlled trials**. *Br J Anaesth* 2015, **114**(3):396-405.

598. Tajaate N, Schreiber JU, Fuchs-Buder T, Jelting Y, Kranke P: **Neostigmine-based reversal of intermediate acting neuromuscular blocking agents to prevent postoperative residual paralysis: A systematic review**. *Eur J Anaesthesiol* 2018, **35**(3):184-192.

599. Tan JA, Ho KM: **Use of remifentanil as a sedative agent in critically ill adult patients: a meta-analysis**. *Anaesthesia* 2009, **64**(12):1342-1352.

600. Terkawi AS, Mavridis D, Flood P, Wetterslev J, Terkawi RS, Bin Abdulhak AA, Nunemaker MS, Tiouririne M: **Does Ondansetron Modify Sympathectomy Due to Subarachnoid Anesthesia?: Meta-analysis, Meta-regression, and Trial Sequential Analysis**. *Anesthesiology* 2016, **124**(4):846-869.

601. Terkawi AS, Mavridis D, Sessler DI, Nunemaker MS, Doais KS, Terkawi RS, Terkawi YS, Petropoulou M, Nemergut EC: **Pain Management Modalities after Total Knee Arthroplasty: A Network Meta-analysis of 170 Randomized Controlled Trials**. *Anesthesiology* 2017, **126**(5):923-937.

602. Tesarz J, Schuster AK, Hartmann M, Gerhardt A, Eich W: **Pain perception in athletes compared to normally active controls: a systematic review with meta-analysis**. *Pain* 2012, **153**(6):1253-1262.

603. Thavaneswaran P, Rudkin GE, Cooter RD, Moyes DG, Perera CL, Maddern GJ: **Brief reports: paravertebral block for anesthesia: a systematic review**. *Anesth Analg* 2010, **110**(6):1740-1744.

604. Thiruvenkatarajan V, Van Wijk RM, Rajbhoj A: **Cranial nerve injuries with supraglottic airway devices: a systematic review of published case reports and series**. *Anaesthesia* 2015, **70**(3):344-359.

605. Thy M, Montmayeur J, Julien-Marsollier F, Michelet D, Brasher C, Dahmani S, Orliaguet G: **Safety and efficacy of peri-operative administration of hydroxyethyl starch in children undergoing surgery: A systematic review and meta-analysis**. *Eur J Anaesthesiol* 2018, **35**(7):484-495.

606. Tiippana EM, Hamunen K, Kontinen VK, Kalso E: **Do surgical patients benefit from perioperative gabapentin/pregabalin? A systematic review of efficacy and safety**. *Anesth Analg* 2007, **104**(6):1545-1556, table of contents.

607. Togioka B, Galvagno S, Sumida S, Murphy J, Ouanes JP, Wu C: **The role of perioperative high inspired oxygen therapy in reducing surgical site infection: a meta-analysis**. *Anesth Analg* 2012, **114**(2):334-342.

608. Toner AJ, Ganeshanathan V, Chan MT, Ho KM, Corcoran TB: **Safety of Perioperative Glucocorticoids in Elective Noncardiac Surgery: A Systematic Review and Meta-analysis**. *Anesthesiology* 2017, **126**(2):234-248.

609. Touray ST, de Leeuw MA, Zuurmond WW, Perez RS: **Psoas compartment block for lower extremity surgery: a meta-analysis**. *Br J Anaesth* 2008, **101**(6):750-760.

610. Tracy LM, Ioannou L, Baker KS, Gibson SJ, Georgiou-Karistianis N, Giummarra MJ: **Meta-analytic evidence for decreased heart rate variability in chronic pain implicating parasympathetic nervous system dysregulation**. *Pain* 2016, **157**(1):7-29.

611. Tran DTT, Newton EK, Mount VAH, Lee JS, Mansour C, Wells GA, Perry JJ: **Rocuronium vs. succinylcholine for rapid sequence intubation: a Cochrane systematic review**. *Anaesthesia* 2017, **72**(6):765-777.

612. Tremont-Lukats IW, Challapalli V, McNicol ED, Lau J, Carr DB: **Systemic administration of local anesthetics to relieve neuropathic pain: a systematic review and meta-analysis**. *Anesth Analg* 2005, **101**(6):1738-1749.

613. Trescot AM, Chopra P, Abdi S, Datta S, Schultz DM: **Systematic review of effectiveness and complications of adhesiolysis in the management of chronic spinal pain: an update**. *Pain Physician* 2007, **10**(1):129-146.

614. Tschopp C, Tramer MR, Schneider A, Zaarour M, Elia N: **Benefit and Harm of Adding Epinephrine to a Local Anesthetic for Neuraxial and Locoregional Anesthesia: A Meta-analysis of Randomized Controlled Trials With Trial Sequential Analyses**. *Anesth Analg* 2018, **127**(1):228-239.

615. Tsur A, Kalansky A: **Hypersensitivity associated with sugammadex administration: a systematic review**. *Anaesthesia* 2014, **69**(11):1251-1257.

616. Uhlig C, Bluth T, Schwarz K, Deckert S, Heinrich L, De Hert S, Landoni G, Serpa Neto A, Schultz MJ, Pelosi P *et al*: **Effects of Volatile Anesthetics on Mortality and Postoperative Pulmonary and Other Complications in Patients Undergoing Surgery: A Systematic Review and Meta-analysis**. *Anesthesiology* 2016, **124**(6):1230-1245.

617. Uppal V, Retter S, Shanthanna H, Prabhakar C, McKeen DM: **Hyperbaric Versus Isobaric Bupivacaine for Spinal Anesthesia: Systematic Review and Meta-analysis for Adult Patients Undergoing Noncesarean Delivery Surgery**. *Anesth Analg* 2017, **125**(5):1627-1637.

618. Usichenko TI, Lehmann C, Ernst E: **Auricular acupuncture for postoperative pain control: a systematic review of randomised clinical trials**. *Anaesthesia* 2008, **63**(12):1343-1348.

619. van Middelkoop M, Rubinstein SM, Ostelo R, van Tulder MW, Peul W, Koes BW, Verhagen AP: **No additional value of fusion techniques on anterior discectomy for neck pain: a systematic review**. *Pain* 2012, **153**(11):2167-2173.

620. Vanlinthout LE, Mesfin SH, Hens N, Vanacker BF, Robertson EN, Booij LH: **A systematic review and meta-regression analysis of mivacurium for tracheal intubation**. *Anaesthesia* 2014, **69**(12):1377-1387.

621. Varela-Lema L, Lopez-Garcia M, Maceira-Rozas M, Munoz-Garzon V: **Linear accelerator stereotactic radiosurgery for trigeminal neuralgia**. *Pain Physician* 2015, **18**(1):15-27.

622. Veehof MM, Oskam MJ, Schreurs KM, Bohlmeijer ET: **Acceptance-based interventions for the treatment of chronic pain: a systematic review and meta-analysis**. *Pain* 2011, **152**(3):533-542.

623. Veldeman M, Hollig A, Clusmann H, Stevanovic A, Rossaint R, Coburn M: **Delayed cerebral ischaemia prevention and treatment after aneurysmal subarachnoid haemorrhage: a systematic review**. *Br J Anaesth* 2016, **117**(1):17-40.

624. Verhagen AP, van Middelkoop M, Rubinstein SM, Ostelo R, Jacobs W, Peul W, Koes BW, van Tulder MW: **Effect of various kinds of cervical spinal surgery on clinical outcomes: a systematic review and meta-analysis**. *Pain* 2013, **154**(11):2388-2396.

625. Vlassakov KV, Narang S, Kissin I: **Local anesthetic blockade of peripheral nerves for treatment of neuralgias: systematic analysis**. *Anesth Analg* 2011, **112**(6):1487-1493.

626. von Baeyer CL, Spagrud LJ: **Systematic review of observational (behavioral) measures of pain for children and adolescents aged 3 to 18 years**. *Pain* 2007, **127**(1-2):140-150.

627. von Elm E, Schoettker P, Henzi I, Osterwalder J, Walder B: **Pre-hospital tracheal intubation in patients with traumatic brain injury: systematic review of current evidence**. *Br J Anaesth* 2009, **103**(3):371-386.

628. Vorobeichik L, Brull R, Abdallah FW: **Evidence basis for using perineural dexmedetomidine to enhance the quality of brachial plexus nerve blocks: a systematic review and meta-analysis of randomized controlled trials**. *Br J Anaesth* 2017, **118**(2):167-181.

629. Vorobeichik L, Brull R, Bowry R, Laffey JG, Abdallah FW: **Should continuous rather than single-injection interscalene block be routinely offered for major shoulder surgery? A meta-analysis of the analgesic and side-effects profiles**. *Br J Anaesth* 2018, **120**(4):679-692.

630. Vowles KE, McEntee ML, Julnes PS, Frohe T, Ney JP, van der Goes DN: **Rates of opioid misuse, abuse, and addiction in chronic pain: a systematic review and data synthesis**. *Pain* 2015, **156**(4):569-576.

631. Waldron NH, Jones CA, Gan TJ, Allen TK, Habib AS: **Impact of perioperative dexamethasone on postoperative analgesia and side-effects: systematic review and meta-analysis**. *Br J Anaesth* 2013, **110**(2):191-200.

632. Walter C, Lotsch J: **Meta-analysis of the relevance of the OPRM1 118A>G genetic variant for pain treatment**. *Pain* 2009, **146**(3):270-275.

633. Wang C, Sun J, Zheng J, Guo L, Ma H, Zhang Y, Zhang F, Li E: **Low-dose hydrocortisone therapy attenuates septic shock in adult patients but does not reduce 28-day mortality: a meta-analysis of randomized controlled trials**. *Anesth Analg* 2014, **118**(2):346-357.

634. Wang G, Bainbridge D, Martin J, Cheng D: **The efficacy of an intraoperative cell saver during cardiac surgery: a meta-analysis of randomized trials**. *Anesth Analg* 2009, **109**(2):320-330.

635. Wang H, Sribastav SS, Ye F, Yang C, Wang J, Liu H, Zheng Z: **Comparison of Percutaneous Vertebroplasty and Balloon Kyphoplasty for the Treatment of Single Level Vertebral Compression Fractures: A Meta-analysis of the Literature**. *Pain Physician* 2015, **18**(3):209-222.

636. Wang L, Chang Y, Kennedy SA, Hong PJ, Chow N, Couban RJ, McCabe RE, Bieling PJ, Busse JW: **Perioperative psychotherapy for persistent post-surgical pain and physical impairment: a meta-analysis of randomised trials**. *Br J Anaesth* 2018, **120**(6):1304-1314.

637. Wang TT, Sun S, Huang SQ: **Effects of Epidural Labor Analgesia With Low Concentrations of Local Anesthetics on Obstetric Outcomes: A Systematic Review and Meta-analysis of Randomized Controlled Trials**. *Anesth Analg* 2017, **124**(5):1571-1580.

638. Wang W, Sun YH, Wang YY, Wang YT, Wang W, Li YQ, Wu SX: **Treatment of functional chest pain with antidepressants: a meta-analysis**. *Pain Physician* 2012, **15**(2):E131-142.

639. Warnell I, Chincholkar M, Eccles M: **Predicting perioperative mortality after oesophagectomy: a systematic review of performance and methods of multivariate models**. *Br J Anaesth* 2015, **114**(1):32-43.

640. Waterschoot FP, Dijkstra PU, Hollak N, de Vries HJ, Geertzen JH, Reneman MF: **Dose or content? Effectiveness of pain rehabilitation programs for patients with chronic low back pain: a systematic review**. *Pain* 2014, **155**(1):179-189.

641. Weibel S, Jokinen J, Pace NL, Schnabel A, Hollmann MW, Hahnenkamp K, Eberhart LH, Poepping DM, Afshari A, Kranke P: **Efficacy and safety of intravenous lidocaine for postoperative analgesia and recovery after surgery: a systematic review with trial sequential analysis**. *Br J Anaesth* 2016, **116**(6):770-783.

642. Weibel S, Neubert K, Jelting Y, Meissner W, Wockel A, Roewer N, Kranke P: **Incidence and severity of chronic pain after caesarean section: A systematic review with meta-analysis**. *Eur J Anaesthesiol* 2016, **33**(11):853-865.

643. Weijs TJ, Dieleman JM, Ruurda JP, Kroese AC, Knape HJ, van Hillegersberg R: **The effect of perioperative administration of glucocorticoids on pulmonary complications after transthoracic oesophagectomy: a systematic review and meta-analysis**. *Eur J Anaesthesiol* 2014, **31**(12):685-694.

644. Werner MU, Bischoff JM, Rathmell JP, Kehlet H: **Pulsed radiofrequency in the treatment of persistent pain after inguinal herniotomy: a systematic review**. *Reg Anesth Pain Med* 2012, **37**(3):340-343.

645. Werner MU, Mjobo HN, Nielsen PR, Rudin A: **Prediction of postoperative pain: a systematic review of predictive experimental pain studies**. *Anesthesiology* 2010, **112**(6):1494-1502.

646. Wersocki E, Bedson J, Chen Y, LeResche L, Dunn KM: **Comprehensive systematic review of long-term opioids in women with chronic noncancer pain and associated reproductive dysfunction (hypothalamic-pituitary-gonadal axis disruption)**. *Pain* 2017, **158**(1):8-16.

647. White L, Halpin A, Turner M, Wallace L: **Ultrasound-guided radial artery cannulation in adult and paediatric populations: a systematic review and meta-analysis**. *Br J Anaesth* 2016, **116**(5):610-617.

648. White LD, Hodge A, Vlok R, Hurtado G, Eastern K, Melhuish TM: **Efficacy and adverse effects of buprenorphine in acute pain management: systematic review and meta-analysis of randomised controlled trials**. *Br J Anaesth* 2018, **120**(4):668-678.

649. Wiesbauer F, Schlager O, Domanovits H, Wildner B, Maurer G, Muellner M, Blessberger H, Schillinger M: **Perioperative beta-blockers for preventing surgery-related mortality and morbidity: a systematic review and meta-analysis**. *Anesth Analg* 2007, **104**(1):27-41.

650. Wijayasinghe N, Andersen KG, Kehlet H: **Neural blockade for persistent pain after breast cancer surgery**. *Reg Anesth Pain Med* 2014, **39**(4):272-278.

651. Wikkelso A, Wetterslev J, Moller AM, Afshari A: **Thromboelastography (TEG) or rotational thromboelastometry (ROTEM) to monitor haemostatic treatment in bleeding patients: a systematic review with meta-analysis and trial sequential analysis**. *Anaesthesia* 2017, **72**(4):519-531.

652. Williams MR, McKeown A, Dexter F, Miner JR, Sessler DI, Vargo J, Turk DC, Dworkin RH: **Efficacy Outcome Measures for Procedural Sedation Clinical Trials in Adults: An ACTTION Systematic Review**. *Anesth Analg* 2016, **122**(1):152-170.

653. Williams MR, Nayshtut M, Hoefnagel A, McKeown A, Carlson DW, Cravero J, Lightdale J, Mason KP, Wilson S, Turk DC *et al*: **Efficacy Outcome Measures for Pediatric Procedural Sedation Clinical Trials: An ACTTION Systematic Review**. *Anesth Analg* 2018, **126**(3):956-967.

654. Willner D, Spennati V, Stohl S, Tosti G, Aloisio S, Bilotta F: **Spine Surgery and Blood Loss: Systematic Review of Clinical Evidence**. *Anesth Analg* 2016, **123**(5):1307-1315.

655. Wong AY, Parent EC, Funabashi M, Stanton TR, Kawchuk GN: **Do various baseline characteristics of transversus abdominis and lumbar multifidus predict clinical outcomes in nonspecific low back pain? A systematic review**. *Pain* 2013, **154**(12):2589-2602.

656. Wong K, Phelan R, Kalso E, Galvin I, Goldstein D, Raja S, Gilron I: **Antidepressant drugs for prevention of acute and chronic postsurgical pain: early evidence and recommended future directions**. *Anesthesiology* 2014, **121**(3):591-608.

657. Wu CL, Cohen SR, Richman JM, Rowlingson AJ, Courpas GE, Cheung K, Lin EE, Liu SS: **Efficacy of postoperative patient-controlled and continuous infusion epidural analgesia versus intravenous patient-controlled analgesia with opioids: a meta-analysis**. *Anesthesiology* 2005, **103**(5):1079-1088; quiz 1109-1010.

658. Wu CL, Rowlingson AJ, Cohen SR, Michaels RK, Courpas GE, Joe EM, Liu SS: **Gender and post-dural puncture headache**. *Anesthesiology* 2006, **105**(3):613-618.

659. Wu LC, Weng PW, Chen CH, Huang YY, Tsuang YH, Chiang CJ: **Literature Review and Meta-Analysis of Transcutaneous Electrical Nerve Stimulation in Treating Chronic Back Pain**. *Reg Anesth Pain Med* 2018, **43**(4):425-433.

660. Wu MC, Liao TY, Lee EM, Chen YS, Hsu WT, Lee MG, Tsou PY, Chen SC, Lee CC: **Administration of Hypertonic Solutions for Hemorrhagic Shock: A Systematic Review and Meta-analysis of Clinical Trials**. *Anesth Analg* 2017, **125**(5):1549-1557.

661. Wu SY, Ling Q, Cao LH, Wang J, Xu MX, Zeng WA: **Real-time two-dimensional ultrasound guidance for central venous cannulation: a meta-analysis**. *Anesthesiology* 2013, **118**(2):361-375.

662. Xie J, Zhang X, Xu J, Zhang Z, Klingensmith NJ, Liu S, Pan C, Yang Y, Qiu H: **Effect of Remote Ischemic Preconditioning on Outcomes in Adult Cardiac Surgery: A Systematic Review and Meta-analysis of Randomized Controlled Studies**. *Anesth Analg* 2018, **127**(1):30-38.

663. Xu J, Yang J, Lin P, Rosenquist E, Cheng J: **Intravenous Therapies for Complex Regional Pain Syndrome: A Systematic Review**. *Anesth Analg* 2016, **122**(3):843-856.

664. Xu JY, Chen QH, Liu SQ, Pan C, Xu XP, Han JB, Xie JF, Huang YZ, Guo FM, Yang Y *et al*: **The Effect of Early Goal-Directed Therapy on Outcome in Adult Severe Sepsis and Septic Shock Patients: A Meta-Analysis of Randomized Clinical Trials**. *Anesth Analg* 2016, **123**(2):371-381.

665. Xu R, Zhu Y, Fan Q, Shen X, Li WX: **Comparison Between the Cobra Perilaryngeal Airway and Laryngeal Mask Airways Under General Anesthesia: A Systematic Review and Meta-analysis**. *Anesth Analg* 2017, **125**(3):958-966.

666. Yang LY, Wang XL, Zhou L, Fu Q: **A systematic review and meta-analysis of randomized controlled trials of unilateral versus bilateral kyphoplasty for osteoporotic vertebral compression fractures**. *Pain Physician* 2013, **16**(4):277-290.

667. Yousaf F, Seet E, Venkatraghavan L, Abrishami A, Chung F: **Efficacy and safety of melatonin as an anxiolytic and analgesic in the perioperative period: a qualitative systematic review of randomized trials**. *Anesthesiology* 2010, **113**(4):968-976.

668. Youssef N, Orlov D, Alie T, Chong M, Cheng J, Thabane L, Paul J: **What epidural opioid results in the best analgesia outcomes and fewest side effects after surgery?: a meta-analysis of randomized controlled trials**. *Anesth Analg* 2014, **119**(4):965-977.

669. Yu SK, Tait G, Karkouti K, Wijeysundera D, McCluskey S, Beattie WS: **The safety of perioperative esmolol: a systematic review and meta-analysis of randomized controlled trials**. *Anesth Analg* 2011, **112**(2):267-281.

670. Yung EM, Brull R, Albrecht E, Joshi GP, Abdallah FW: **Evidence Basis for Regional Anesthesia in Ambulatory Anterior Cruciate Ligament Reconstruction: Part III: Local Instillation Analgesia-A Systematic Review and Meta-analysis**. *Anesth Analg* 2019, **128**(3):426-437.

671. Zeng AM, Nami NF, Wu CL, Murphy JD: **The Analgesic Efficacy of Nonsteroidal Anti-inflammatory Agents (NSAIDs) in Patients Undergoing Cesarean Deliveries: A Meta-Analysis**. *Reg Anesth Pain Med* 2016, **41**(6):763-772.

672. Zhang J, Ho KY, Wang Y: **Efficacy of pregabalin in acute postoperative pain: a meta-analysis**. *Br J Anaesth* 2011, **106**(4):454-462.

673. Zheng F, Sheinberg R, Yee MS, Ono M, Zheng Y, Hogue CW: **Cerebral near-infrared spectroscopy monitoring and neurologic outcomes in adult cardiac surgery patients: a systematic review**. *Anesth Analg* 2013, **116**(3):663-676.

674. Zhou X, Zhang C, Wang Y, Yu L, Yan M: **Preoperative Acute Normovolemic Hemodilution for Minimizing Allogeneic Blood Transfusion: A Meta-Analysis**. *Anesth Analg* 2015, **121**(6):1443-1455.

675. Zhu A, Benzon HA, Anderson TA: **Evidence for the Efficacy of Systemic Opioid-Sparing Analgesics in Pediatric Surgical Populations: A Systematic Review**. *Anesth Analg* 2017, **125**(5):1569-1587.

676. Zorrilla-Vaca A, Grant MC, Mathur V, Li J, Wu CL: **The Impact of Neuraxial Versus General Anesthesia on the Incidence of Postoperative Surgical Site Infections Following Knee or Hip Arthroplasty: A Meta-Analysis**. *Reg Anesth Pain Med* 2016, **41**(5):555-563.

677. Zorrilla-Vaca A, Mathur V, Wu CL, Grant MC: **The Impact of Spinal Needle Selection on Postdural Puncture Headache: A Meta-Analysis and Metaregression of Randomized Studies**. *Reg Anesth Pain Med* 2018, **43**(5):502-508.

678. Zou J, Mei X, Zhu X, Shi Q, Yang H: **The long-term incidence of subsequent vertebral body fracture after vertebral augmentation therapy: a systemic review and meta-analysis**. *Pain Physician* 2012, **15**(4):E515-522.
