# Supplementary material for: Methodological tools and sensitivity analysis for assessing quality or risk of bias used in systematic reviews published in the high-impact anesthesiology journals
Source: BMC Med Res Methodol. 2020 May 18;20:121. doi: 10.1186/s12874-020-00966-4 (PMC7236513; doi:10.1186/s12874-020-00966-4)
Supplement: Supplementary file 3 — Additional file 3. Quality threshold for sensitivity analysis as described in the included studies. Threshold description includes all relevant information for sensitivity analysis from Methods, Results or Discussion, regardless of the part of manuscript where they were described. [file 12874_2020_966_MOESM3_ESM.docx]

**Supplementary file 3. Quality threshold for sensitivity analysis as described in the included studies.**

Threshold description includes all relevant information for sensitivity analysis from Methods, Results or Discussion, regardless of the part of manuscript where they were described.

|  | **Study** | **Threshold description** | **Threshold clearly explained?** | **Comment** |
| --- | --- | --- | --- | --- |
|  | Abadgurumeta 2015 | We classified trial bias risk as high if any domain bias was judged as unclear or high. | Yes | Clear threshold description |
|  | Abbas 2008 | Sensitivity analysis was performed by including and excluding the poor quality studies | No | Not reported what is poor quality |
|  | Adesope 2016 | A sensitivity analysis was performed for the primary outcomes excluding studies that had a high risk of bias for any of the domains assessed | Yes | Clear threshold description |
|  | Allen 2017 | Sensitivity analysis for studies with high risk of bias, defined as high risk of bias for one or more key domains | No | The authors did not report what they consider to be key domains |
|  | Aya 2013 | A correction for quality using the Jadad score, with a score ˃3 classified as a higher quality study | Yes | Clear threshold description |
|  | Belleti 2015 | Sensitivity analysis was conducted for studies studies judged to carry a low risk of bias | No | Not reported what is considered a low risk of bias |
|  | Bolton 2006 | A sensitivity analysis was performed which excluded trials with methodological flaws such as a significant loss-to-follow-up, which was defined as a loss-to-follow-up/protocol violation rate of more than 20% | Yes | Clear threshold description |
|  | Braz 2017 | The assessment of risk of bias included a sensitivity analysis that addressed patients lost to follow-up | No | Threshold for losses to follow-up (attrition bias) not reported |
|  | Cai 2013 | Sensitivity analysis was applied by excluding studies with high risk of bias because of incomplete outcome data of pain or single blinding of participants and personnel | Yes | Clear threshold description |
|  | Choi 2014 | Excluding the study at highest risk of bias | No | Not defined what is the highest risk of bias |
|  | Cohen 2018 | Sensitivity analysis excluded studies at hig risk of bias, with risk of bias separately evaluated using objective criteria and the investigators’ judgement. An overall risk of bias was evaluated as low or high using two distinct approaches: first, using strict criteria according to which any single domain rated as having either high or unclear risk of bias was sufficient to rank the study as having an overall high risk of bias. Second, using the investigators’ clinical judgment and perception of the potential effect of every domain on each study’s actual risk of bias. | No | Overall risk of bias on study level not clearly reported (unclear how investigators’ judgment was made) |
|  | Corcoran 2012 | Sensitivity analyses were conducted by restricting the analysis to trials that had both double-blind and adequate allocation concealment | Yes | Clear threshold description |
|  | de Waal 2015 | Sensitivity analyses were performed to estimate differences of excluding studies with unclear, low or high risk of bias. Risk of bias levels were defined with a Cochrane tool | No | Not reported what is considered study at unclear, low or high risk of bias |
|  | Doleman 2015a (br. 104 u tbl) | We performed sensitivity analyses by removing studies at high risk of bias | No | Not reported what is considered study at high risk of bias |
|  | Doleman 2015b (br. 105 u tbl) | Sensitivity analysis was conducted by excluding studies at high risk of bias | No | Not reported what is considered study at high risk of bias |
|  | Dvirnik 2018 | Sensitivity analysis was conducted after including only high-quality studies, based on the absence of one or less methodological quality measures which we had a priori planned. The quality of individual trials was assessed based on methodological completeness, focusing on appropriate randomisation, double blinding, and adequate follow up (Jadad and colleagues). | No | Threshold for high-quality not reported |
|  | Elmi-Sarabi 2017 | Sensitivity analysis was performed by removing studies classifed at high risk of bias | No | Not reported what is considered high risk of bias |
|  | Fayad 2016 | When applicable, we had planned to undertake sensitivity analyses by study risk of bias. For each outcome of interest, we assessed study risk of bias using the Quality In Prognosis Studies tool. The overall study risk of bias was categorized as high, moderate, or low. | No | Not reported how different levels of bias were defined. |
|  | Fletcher 2014 | Sensitivity analyses were performed to estimate differences of excluding studies with unclear, low or high risk of bias. Risk of bias levels were defined with a Cochrane tool | No | Not reported how different levels of bias were defined. |
|  | Fominskiy 2015 | The methodological quality of individual studies(including description of randomization, allocation concealment,blinded assessor, and intention-to-treat data analysis) was as-sessed. We rated the risk of bias by applying a rating of‘Yes’,‘No’or‘Unclear’to denote whether adequate measures weretaken to protect against each potential source of bias in eachstudy. The overall risk of bias was expressed as low, moderate,or high. | No | Not reported how different levels of bias were defined. |
|  | Gillespie 2014 | We estimated patient outcome measures after excluding studies with lower methodological quality to check whether the results had changed. | No | Not defined what is lower methodological quality |
|  | Gopal 2009 | Sensitivity analysis was performed by excluding the studies that had low Jadad scores | No | Not defined what is low Jadad scores |
|  | Grant 2016 | Sensitivity analysis was performed excluding high risk of bias (Jadad score <4) studies | Yes | Clear threshold description |
|  | Greco 2015 | Sensitivity analysis was conducted with low risk of bias trials | No | Not defined what is low risk of bias trials |
|  | Hamilton 2011 | A correction for quality using the Jadad score, with a score 3 classified as a higher quality study | Yes | Clear threshold description |
|  | Hattler 2016 | A sensitivity analysis for blinding of the FHR tracing outcome assessor was planned a priori; that is, one analysis included and another analysis excluded studies in which the FHR outcome assessor was not blinded to group allocation. | Yes | Clear threshold description |
|  | Hauser 2009 | Sensitivity analysis between studies with moderate and high methodological quality | No | Not defined what is moderate and high methodological quality |
|  | Hauser 2011 | Sensitivity analysis for studies with a low (1 to 2) and moderate (3 to 5) Jadad score | Yes | Clear threshold description |
|  | Heesen 2017 | We performed meta-analyses including only trials that we had assessed as low risk of bias and presented these results as our principal results. As a sensitivity analysis, we also performed meta-analyses including all eligible trials independent of risk of bias. We considered a trial to be at low risk of bias when there was adequate random sequence generation, adequate allocation concealment and outcome assessment was adequately blinded. | Yes | Clear threshold description |
|  | Higgins 2018 | Sensitivity analyses were undertaken with risk of bias in individual studies as a moderator variable. Risk of bias within studies was undertaken at study level, rather than outcome level, and assessed using instruments designed by the NIH. All studies were identified as being of either ‘good’ or ‘fair’ quality; none were excluded because of being of ‘poor’ quality. | No | Not reported what is good, fair or poor quality |
|  | Hillyard 2011 | Sensitivity analysis was performed by excluding trials that did not have double blinding, adequate allocation concealment, or a loss to follow-up of ˃10%. | Yes | Clear threshold description |
|  | Jelting 2017 | We performed sensitivity analyses to assess the robustness of the pooled estimates focusing on risk of bias (impact of studies with high risk of selection bias, performance and detection bias, attrition bias) | No | Not defined what is high risk in these domains |
|  | Johnson 2007 | A sensitivity analysis was performed by limiting the analysis to those studies with a Jadad score of at least 4 | Yes | Clear threshold description |
|  | Karam 2016 | Sensitivity analysis assessing the impact of bias assessment | No | Not defined what aspect of bias assessment was used in sensitivity analysis |
|  | Kawakami 2017 | Sensitivity analyses were performed for the primary outcomes according to the risk of bias (low versus high). The risk of bias was classified into 3 categories: “low,” “high,” or “unclear.” Trials with 1 or more Cochrane risk of bias domain that was unclear or at high  risk of bias were considered to be trials at high risk of bias. | Yes | Clear threshold description |
|  | Khan 2016 | A sensitivity analysis on the primary outcome was also planned in which trials that had one or more categories at high risk of bias on the Cochrane risk of bias tool were excluded | Yes | Clear threshold description |
|  | Kim 2014a (#426 u tbl) | Sensitivity analysis was performed on the studies classified as low risk both in the risk of bias domains and applicability concerns domains | No | Not clearly reported what is low or risk of bias |
|  | Kim 2014b (#427 u tbl) | We conducted sensitivity analysis based on risk of bias (low risk vs. high/unclear risk in flow and timing domain according to the modified QUADAS 2) | No | Not reporting what was used as a threshold to discriminate between low, high and unclear risk on the tool |
|  | Kuratani 2008 | Sensitivity analysis was performed by recalculating the pooled OR using data with a study quality rating greater than 4; quality tool which was used was not specified | No | Quality tool which was used not specified |
|  | Kuriyama 2018 | Sensitivity analysis was also performed by excluding trials at high or unclear risk of bias in terms of sequence generation, allocation concealment, blinding of participants and outcome assessors | Yes | Clear threshold description |
|  | Kuriyama 2019 | We also conducted sensitivity analysis by excluding trials of high or unclear risk of bias in sequence generation, allocation concealment and blinding of participants and outcome assessor | Yes | Clear threshold description |
|  | Landoni 2013 | Sensitivity meta-analyses were performed by analysing data from studies with low risk of bias | No | Not defined what is low risk of bias |
|  | Landoni 2015 | Sensitivity pooled analysis were performed including only randomized trials with low risk of bias, and then repeated with studies with low and moderate risk of bias | No | Not defined what is low risk of bias |
|  | Leong 2011 | For studies that were judged to be at higher risk of bias, a sensitivity analysis was performed to assess whether the inclusion of these studies significantly biased the result | No | Not defined what is low risk of bias |
|  | Lewis 2017 | We performed sensitivity analyses to explore the impact of decisions made during risk of bias assessment. Sensitivity analysis was conducted for studies We considered the impact of our risk of bias assessments on our primary outcome of failed intubation. Removing studies which had an unclear or high risk of selection bias and removing studies which had a high risk of attrition bias | Yes | Clear threshold description |
|  | Liu 2018 | We performed sensitivity analyses to exclude studies with high or unclear risk of selection bias to explore the impact on our results | Yes | Clear threshold description |
|  | Martinez 2015 | Sensitivity analysis of trial quality described in results (high vs low risk of bias studies) | No | Not defined what is low and high risk of bias |
|  | Martinez 2017 | We planned a sensitivity analysis excluding studies with a high or unclear risk of bias | No | Not defined what is high and unclear risk of bias |
|  | Maund 2011 | Sensitivity analyses based on trial quality, in terms of whether or not there was adequate blinding | Yes | Clear threshold description |
|  | McIlroy 2009 | We assessed the impact of study quality on outcomes by repeating the analysis following sequential exclusion of studies with less than adequate allocation concealment or inadequate blinding | Yes | Clear threshold description |
|  | McNicol 2011 | Sensitivity analyses were performed to investigate the effect of study characteristics on the primary efficacy outcome: non-blinded studies were included then removed. | Yes | Clear threshold description |
|  | Meng 2017 | We performed a sensitivity analysis by removing the 1 trial with a high risk of bias. A decision to classify “overall bias” as low, unclear, or high was made by the reviewers using the following method: High: any trial with a high risk of bias listed on 3 or more domains. Unclear: any trial with a high risk of bias listed on more than 1 but less than 3 domains. Low: any trial with a high risk of bias on none or 1 domain and with no significant methodologic concerns that may have affected the study results. | Yes | Clear threshold description |
|  | Meylan 2009 | We performed sensitivity analyses to test the impact of the quality of data reporting (i.e. the modified Oxford scale) | No | Threshold not described |
|  | Mihara 2013 | Sensitivity analyses was performed for risk of bias (low versus high or unclear) | No | Not defined what is low, unclear and high risk of bias |
|  | Mihara 2014 | Sensitivity analyses were performed by excluding studies with a high risk of bias | No | Not defined what is high risk of bias |
|  | Mihara 2015 | Sensitivity analyses were performed to confirm whether the overall results would change when restricting the data to studies with high methodological quality. We assessed the risk of bias in sequence generation, allocation sequence concealment, the blinding of  patients, the blinding of healthcare providers, the blinding of data collectors, the blinding of outcome assessors, incomplete outcome data, selective outcome reporting and other biases. We also summarised the risk of bias. | No | Not reported how risk of bias was summarized |
|  | Mihara 2017 | We conducted sensitivity analyses after restricting to high-quality studies (i.e. studies with low risk of bias on both the sequence generation and allocation concealment domains). | Yes | Clear threshold description |
|  | Mishriky 2012 | sensitivity analysis was performed according to the quality of the included studies by restricting the analysis to studies with a modified Oxford score of 4 or higher | Yes | Clear threshold description |
|  | Mishriky 2014 | Sensitivity analysis was performed by excluding studies with high risk of bias for any of the risk of bias parameters assessed. Cochrane levels of bias were used. | Yes | Clear threshold description |
|  | Mishriky 2015 | We performed a sensitivity analysis for the primary outcomes after removing papers with an unclear or high risk of bias | No | Not defined what is unclear and high risk of bias |
|  | Morrison 2013 | Sensitivity analysis was undertaken according to the methodological quality of the included trials [studies with low quality (Jadad score ≤3) vs studies with high quality (Jadad score ˃3)] | Yes | Clear threshold description |
|  | Nagappa 2017 | Sensitivity analysis was done for studies with good, or poor-moderate score based on the Newcastle-Ottawa Scale quality checking. Study was considered good when assigned score was ≥8 of 9. | Yes | Clear threshold description |
|  | Passos 2008 | In order to control for bias of including studies of poor quality, we performed a sensitivity analysis | No | Not defined what is poor quality |
|  | Peerdeman 2016 | Sensitivity analyses assessed the stability of the overall effect size in relation to the risk of bias within studies (by removing studies for which at least 1 item was judged to involve a high risk of bias) | Yes | Clear threshold description |
|  | Raiman 2016 | Sensitivity analyses for all outcomes were also conducted by removing high bias studies (Jadad score <3) from each outcome metaanalysis. | Yes | Clear threshold description |
|  | Sanfilippo 2017 | We planned sensitivity analyses of the primary outcome conducted by excluding studies with intermediate or high risk of bias. As all of the studies had a low risk of bias according to the Newcastle-Ottawa Scale tool (score ranging between 6 and 9), we did not perform a further sensitivity analysis according to the quality of study design | Yes | Clear threshold description |
|  | Schaeffer 2016 | A sensitivity analysis was performed for overall PONV, excluding studies with a high risk of overall bias. Studies were scored for risk of bias using The Cochrane Collaboration ‘Risk Of Bias’ Assessment Tool, which judges risk of bias in six domains: random sequence generation and allocation concealment (selection bias), blinding of participants and study personnel (performance bias), blinding of outcome assessment (detection bias), incomplete outcome data (attrition bias) and selective reporting (reporting bias). Additionally, we estimated the overall risk of bias for each study. | No | Definition of overall risk of bias not reported |
|  | Schnabel 2013a | Sensitivity analysis according to the data quality (high-quality trials [modified Oxford scale > 4] vs low-quality trials [modified Oxford scale ≤ 4) | Yes | Clear threshold description |
|  | Schnabel 2013b | The sensitivity analysis focusing on differences between high-quality trials (modified Oxford scale ˃4) and low-quality trials (modified Oxford scale ≤4) | Yes | Clear threshold description |
|  | Schnabel 2011 | A sensitivity analysis was applied with respect to different methodical quality of the included trials (studies with low quality vs studies with high quality). Only one study scored 2 Oxford points and was rated as low-quality study | Yes | Clear threshold description |
|  | Schnabel 2010 | Sensitivity analysis was applied with respect to different methodical quality of the included trials (studies with low quality vs studies with high quality). According to the result of the Oxford scale, the studies were rated as high (Oxford scale ≥3) or low (Oxford scale ˃3) quality studies. | Yes | Clear threshold description |
|  | Schnabel 2012 | A sensitivity analysis investigated the influence of study quality (‘high quality’: Oxford scale >3 versus ‘low quality’: Oxford scale 3 points) | Yes | Clear threshold description |
|  | Sultan 2016 | Sensitivity analysis comparing studies blinded to allocation to those that were not. | Yes | Clear threshold description |
|  | Sun 2008a | Sensitivity analysis was performed for adequate trial blinding, in which two points were assigned to a blinding item of the validity scale | Yes | Clear threshold description |
|  | Sun 2008b | We also performed sensitivity analyses by restricting the analysis to high-quality RCTs as defined as those trials with validity score of 5 or greater and to RCTs in which both patients and assessor blinding was described and the method of blinding deemed adequate. | Yes | Clear threshold description |
|  | Suppan 2015 | Sensitivity analyses were conducted to check for the robustness of the data by removing each study one by one, excluding lower quality studies (Oxford score <4) | Yes | Clear threshold description |
|  | Terkawi 2016 | We performed sensitivity analyses for the primary outcomes by evaluating the difference in the outcome direction, magnitude, and significance when we removed the studies that scored “unclear” in more than three of the Cochrane risk of bias assessment. | Yes | Clear threshold description |
|  | Terkawi 2017 | Our sensitivity analyses consisted of excluding studies with an overall high risk of bias. We classified studies to be overall at high risk of bias only when they also demonstrated a high risk of bias in at least one other domain of Cochrane RoB tool | Yes | Clear threshold description |
|  | Tesarz 2012 | Sensitivity analyses were performed to determine the effect of low quality studies (by excluding studies with high risk of bias) | No | Not defined what is low quality of high risk of bias |
|  | Togioka 2012 | Sensitivity analyses were performed to evaluate the effect of the lowest quality study | No | Not defined what is lowest quality study |
|  | Toner 2017 | Sensitivity analyses were performed using high-quality trials only (Jadad scale score, 4 to 5). | Yes | Clear threshold description |
|  | Uppal 2017 | Sensitivity analysis was conducted by excluding studies that were judged “high” RoB | No | Not reported what was considered as high RoB |
|  | Vanlinthout 2014 | Sensitivity analysis evaluated effect of allocation concealment and blinding the assessor | No | Threshold criteria not defined |
|  | Verhagen 2013 | Sensitivity analysis was based on risk of bias | No | Threshold criteria not defined |
|  | Wang 2009 | Sensitivity analysis was planned for study quality (Jadad score ≥3 vs Jadad score≤ 3) and for blinded versus nonblinded trials | Yes | Clear threshold description |
|  | Wang 2016 | Sensitivity analyses were conducted to check the robustness of the data by excluding studies with a high risk of bias. The quality of each study included in the meta-analysis was reviewed with the Cochrane Collaboration’s tool for assessing the risk of bias.27 The quality assessment methodology included the evaluation of random sequence generation, allocation concealment, the blinding of participants and personnel, the blinding of the outcome assessment, incomplete outcome data, selective reporting, and other bias. The overall quality was graded as a low risk of bias, a high risk of bias, or an unclear risk of bias for each domain via the use of a standardized tool.27 | No | Not described what was low, high or unclear risk of bias on study level |
|  | Waterschoot 2014 | To analyze the influence of lack of blinding of care providers on the judgment of methodological quality, we performed a sensitivity analysis | Yes | Clear threshold description |
|  | Weibel 2016 | We excluded all trials which were identified as high risk of bias (= judged as high risk at least in one risk of bias domain or as unclear risk of bias in all domains) and performed a sensitivity meta-analysis to test robustness of the results | Yes | Clear threshold description |
|  | Wong 2013 | The maximum score of this checklist is 26. The cutoff point for distinguishing a high-quality study is 50% of maximum score [10]. A study with higher scores indicates that the study has lower risk of bias. Because the cutoff point was arbitrary, sensitivity analyses using 60% and 70% cutoff points were conducted to test the robustness of these cutoff points. | Yes | Clear threshold description |
|  | Wong 2014 | Sensitivity analyses would be used to evaluate the robustness of a particular result by repeating primary analyses with respect to study quality | No | Not defined what is quality threshold |
